# Supplementary material for: Month of birth and risk of autism spectrum disorder: a retrospective cohort of male children born in Israel
Source: BMJ Open. 2017 Nov 16;7(11):e014606. doi: 10.1136/bmjopen-2016-014606 (PMC5702026; doi:10.1136/bmjopen-2016-014606)
Supplement: Supplementary data 1 [file bmjopen-2016-014606supp001.pdf]

Appendix

Table a: Crude and adjusted odd ratio (OR) and 95% confidence interval from multivariable logistic regression of autistic spectrum disorder (ASD) for first month of second trimester, by socioeconomic status (SES)

Month of second trimester	OR crude	95%CI		OR adjusted*	95%CI		OR crude	95%CI		OR adjusted*	95%CI	
**Jan**	1 (ref.)			1 (ref.			1 (ref.)			1 (ref.		
**Feb**	.83	.51	1.35	.83	.51	1.36	1.03	.67	1.57	1.03	.67	1.57
**Mar**	1.08	.69	1.69	1.08	.69	1.69	.87	.57	1.35	.86	.56	1.33
**Apr**	.89	.55	1.43	.89	.55	1.43	1.12	.74	1.69	1.11	.73	1.67
**May**	.68	.41	1.12	.68	.41	1.13	.99	.65	1.51	.98	.64	1.49
**Jun**	.87	.53	1.41	.87	.54	1.41	1.09	.72	1.64	1.07	.71	1.63
**Jul**	1.00	.62	1.59	.98	.61	1.56	1.02	.67	1.56	1.04	.68	1.59
**Aug**	.74	.44	1.23	.72	.43	1.20	.97	.63	1.49	.97	.63	1.49
**Sep**	.94	.58	1.54	.93	.57	1.51	.86	.55	1.35	.87	.55	1.36
**Oct**	.84	.51	1.37	.83	.51	1.37	1.10	.73	1.66	1.11	.73	1.67
**Nov**	.84	.51	1.40	.83	.50	1.38	1.25	.84	1.87	1.24	.83	1.86
**Dec**	.83	.51	1.36	.83	.51	1.36	1.10	.73	1.65	1.09	.72	1.64

*Adjusted for child's age, father's age, mother's age, district, birth weight, age with imputation (see text for details).

Table b: Crude and adjusted odd ratio (OR) and 95% confidence interval from multivariable logistic regression of autistic spectrum disorder (ASD) for first month of third trimester, by socioeconomic status (SES)

**Month of third trimester**	**OR crude**	**95%CI**		**OR adjusted***	**95%CI**		**OR crude**	**95%CI**		**OR adjusted***	**95%CI**	
Jan	1 (ref.)			1 (ref.			1 (ref.)			1 (ref.		
Feb	1.04	0.61	1.77	1.04	0.61	1.78	1.22	0.81	1.82	1.21	0.81	1.81
Mar	0.97	0.57	1.63	0.97	0.58	1.64	0.96	0.63	1.46	0.95	0.63	1.45
Apr	1.19	0.73	1.95	1.20	0.74	1.97	1.13	0.75	1.68	1.12	0.75	1.68
May	0.86	0.51	1.46	0.87	0.52	1.48	0.86	0.56	1.32	0.86	0.56	1.31
Jun	1.26	0.77	2.04	1.27	0.78	2.06	0.84	0.55	1.30	0.83	0.54	1.27
Jul	0.99	0.60	1.65	1.01	0.61	1.67	1.07	0.71	1.61	1.05	0.70	1.58
Aug	0.78	0.45	1.33	0.79	0.46	1.36	0.99	0.66	1.51	0.98	0.64	1.48
Sep	1.12	0.68	1.86	1.12	0.68	1.86	1.12	0.75	1.69	1.12	0.74	1.69
Oct	0.84	0.49	1.44	0.83	0.48	1.42	0.83	0.53	1.29	0.83	0.54	1.30
Nov	0.92	0.53	1.57	0.91	0.53	1.56	1.00	0.65	1.53	1.00	0.65	1.54
Dec	1.07	0.64	1.80	1.08	0.64	1.81	0.81	0.52	1.26	0.81	0.52	1.26

*Adjusted for child's age, father's age, mother's age, district, birth weight, age with imputation (see text for details).

Table c: Crude and adjusted odd ratio (OR) and 95% confidence interval from multivariable logistic regression of autistic spectrum disorder (ASD) for season of birth, by socioeconomic status (SES)

	SES median-high						SES median-low					
**Season of birth**	**OR crude**	**95%CI**		**OR adjusted***	**95%CI**		**OR crude**	**95%CI**		**OR adjusted***	**95%CI**	
Winter	1 (ref.)			1 (ref.			1 (ref.)			1 (ref.		
Spring	1.12	0.84	1.50	1.12	0.84	1.50	1.06	0.83	1.34	1.06	0.83	1.34
Summer	1.24	0.94	1.63	1.23	0.93	1.63	1.08	0.85	1.37	1.08	0.85	1.37
Fall	1.14	0.86	1.51	1.14	0.86	1.51	1.05	0.82	1.33	1.04	0.82	1.32

*Adjusted for child's age, father’s age, mother’s age, district, birth weight, gestational age with imputation

Table d: Crude and adjusted odd ratio (OR) and 95% confidence interval from multivariable logistic regression of autistic spectrum disorder (ASD) for season of conception, by socioeconomic status (SES)

	SES median-high						SES median-low					
**Season of conception**	**OR crude**	**95%CI**		**OR adjusted***	**95%CI**		**OR crude**	**95%CI**		**OR adjusted***	**95%CI**	
**Winter**	1 (ref.)			1 (ref.			1 (ref.)			1 (ref.		
**Spring**	1.00	0.75	1.34	0.99	0.74	1.33	0.96	0.75	1.22	0.96	0.75	1.23
**Summer**	1.00	0.74	1.35	0.99	0.73	1.33	0.96	0.75	1.22	0.97	0.76	1.24
**Fall**	1.09	0.82	1.45	1.08	0.82	1.44	1.05	0.83	1.33	1.06	0.83	1.34

*Adjusted for child's age, father's age, mother's age, district, birth weight, gestational age with imputation

Table e: Crude and adjusted odd ratio (OR) and 95% confidence interval from multivariable logistic regression of autistic spectrum disorder (ASD) for month of conception, by socioeconomic status (SES)

SES 1-6 SES 7-10

Month of conception	OR crude	95%CI		OR adjusted*	95%CI		OR crude	95%CI		OR adjusted*	95%CI	
Jan	1 (ref.)			1 (ref.			1 (ref.)			1 (ref.		
Feb	.89	.52	1.50	.89	.52	1.51	1.09	.72	1.65	1.08	.72	1.63
Mar	1.13	.70	1.85	1.14	.70	1.86	.84	.55	1.30	.84	.54	1.29
Apr	.90	.53	1.53	.89	.53	1.52	1.06	.71	1.60	1.09	.72	1.64
May	1.05	.63	1.75	1.03	.62	1.71	.94	.62	1.44	.95	.62	1.45
Jun	1.19	.72	1.96	1.17	.71	1.93	.74	.46	1.16	.75	.47	1.18
Jul	.79	.45	1.38	.79	.45	1.38	.96	.63	1.45	.97	.64	1.47
Aug	1.11	.67	1.83	1.09	.66	1.81	1.13	.76	1.68	1.13	.76	1.68
Sep	1.29	.79	2.10	1.28	.78	2.08	1.09	.73	1.64	1.10	.73	1.65
Oct	.95	.57	1.58	.96	.58	1.59	.88	.58	1.34	.89	.58	1.35
Nov	1.14	.70	1.86	1.14	.70	1.86	1.16	.78	1.72	1.16	.78	1.72
Dec	1.18	.74	1.91	1.18	.74	1.91	.90	.59	1.36	.89	.59	1.36

*Adjusted for child's age, father’s age, mother’s age, district, birth weight, age with imputation (see text for details).
