# Supplementary material for: Month of birth and risk of autism spectrum disorder: a retrospective cohort of male children born in Israel
Source: BMJ Open. 2017 Nov 16;7(11):e014606. doi: 10.1136/bmjopen-2016-014606 (PMC5702026; doi:10.1136/bmjopen-2016-014606)
Supplement: Supplementary data 2 [file bmjopen-2016-014606supp002.docx]

Appendix

Table a: Crude and adjusted odd ratio (OR) and 95% confidence interval from multivariable logistic regression of autistic spectrum disorder (ASD) for first month of second trimester, by socioeconomic status (SES)

| **Month of second trimester** | **OR crude** | **95%CI** | | **OR** | **95%CI** | | **OR crude** | **95%CI** | | **OR** | **95%CI** | |
| --- | --- | --- | --- | --- | --- | --- | --- | --- | --- | --- | --- | --- |
|  |  |  |  | **adjusted*** |  |  |  |  |  | **adjusted*** |  |  |
| **Jan** | 1 (ref.) |  |  | 1 (ref.) |  | | 1 (ref.) |  |  | 1 (ref.) |  | |
| **Feb** | .83 | .51 | 1.35 | .83 | .51 | 1.36 | 1.03 | .67 | 1.57 | 1.03 | .67 | 1.57 |
| **Mar** | 1.08 | .69 | 1.69 | 1.08 | .69 | 1.69 | .87 | .57 | 1.35 | .86 | .56 | 1.33 |
| **Apr** | .89 | .55 | 1.43 | .89 | .55 | 1.43 | 1.12 | .74 | 1.69 | 1.11 | .73 | 1.67 |
| **May** | .68 | .41 | 1.12 | .68 | .41 | 1.13 | .99 | .65 | 1.51 | .98 | .64 | 1.49 |
| **Jun** | .87 | .53 | 1.41 | .87 | .54 | 1.41 | 1.09 | .72 | 1.64 | 1.07 | .71 | 1.63 |
| **Jul** | 1.00 | .62 | 1.59 | .98 | .61 | 1.56 | 1.02 | .67 | 1.56 | 1.04 | .68 | 1.59 |
| **Aug** | .74 | .44 | 1.23 | .72 | .43 | 1.20 | .97 | .63 | 1.49 | .97 | .63 | 1.49 |
| **Sep** | .94 | .58 | 1.54 | .93 | .57 | 1.51 | .86 | .55 | 1.35 | .87 | .55 | 1.36 |
| **Oct** | .84 | .51 | 1.37 | .83 | .51 | 1.37 | 1.10 | .73 | 1.66 | 1.11 | .73 | 1.67 |
| **Nov** | .84 | .51 | 1.40 | .83 | .50 | 1.38 | 1.25 | .84 | 1.87 | 1.24 | .83 | 1.86 |
| **Dec** | .83 | .51 | 1.36 | .83 | .51 | 1.36 | 1.10 | .73 | 1.65 | 1.09 | .72 | 1.64 |
| *Adjusted for child's age, father’s age, mother’s age, district, birth weight, age with imputation (see text for details). | | | | | | | | | |  |  |  |

Table b: Crude and adjusted odd ratio (OR) and 95% confidence interval from multivariable logistic regression of autistic spectrum disorder (ASD) for first month of third trimester, by socioeconomic status (SES)

| **Month of third trimester** | **OR crude** | **95%CI** | | **OR** | **95%CI** | | **OR crude** | **95%CI** | | **OR** | **95%CI** | |
| --- | --- | --- | --- | --- | --- | --- | --- | --- | --- | --- | --- | --- |
|  |  |  |  | **adjusted*** |  |  |  |  |  | **adjusted*** |  |  |
| Jan | 1 (ref.) |  |  | 1 (ref.) |  | | 1 (ref.) |  |  | 1 (ref.) |  | |
| Feb | 1.04 | 0.61 | 1.77 | 1.04 | 0.61 | 1.78 | 1.22 | 0.81 | 1.82 | 1.21 | 0.81 | 1.81 |
| Mar | 0.97 | 0.57 | 1.63 | 0.97 | 0.58 | 1.64 | 0.96 | 0.63 | 1.46 | 0.95 | 0.63 | 1.45 |
| Apr | 1.19 | 0.73 | 1.95 | 1.20 | 0.74 | 1.97 | 1.13 | 0.75 | 1.68 | 1.12 | 0.75 | 1.68 |
| May | 0.86 | 0.51 | 1.46 | 0.87 | 0.52 | 1.48 | 0.86 | 0.56 | 1.32 | 0.86 | 0.56 | 1.31 |
| Jun | 1.26 | 0.77 | 2.04 | 1.27 | 0.78 | 2.06 | 0.84 | 0.55 | 1.30 | 0.83 | 0.54 | 1.27 |
| Jul | 0.99 | 0.60 | 1.65 | 1.01 | 0.61 | 1.67 | 1.07 | 0.71 | 1.61 | 1.05 | 0.70 | 1.58 |
| Aug | 0.78 | 0.45 | 1.33 | 0.79 | 0.46 | 1.36 | 0.99 | 0.66 | 1.51 | 0.98 | 0.64 | 1.48 |
| Sep | 1.12 | 0.68 | 1.86 | 1.12 | 0.68 | 1.86 | 1.12 | 0.75 | 1.69 | 1.12 | 0.74 | 1.69 |
| Oct | 0.84 | 0.49 | 1.44 | 0.83 | 0.48 | 1.42 | 0.83 | 0.53 | 1.29 | 0.83 | 0.54 | 1.30 |
| Nov | 0.92 | 0.53 | 1.57 | 0.91 | 0.53 | 1.56 | 1.00 | 0.65 | 1.53 | 1.00 | 0.65 | 1.54 |
| Dec | 1.07 | 0.64 | 1.80 | 1.08 | 0.64 | 1.81 | 0.81 | 0.52 | 1.26 | 0.81 | 0.52 | 1.26 |

*Adjusted for child's age, father’s age, mother’s age, district, birth weight, age with imputation (see text for details).

Table c: Crude and adjusted odd ratio (OR) and 95% confidence interval from multivariable logistic regression of autistic spectrum disorder (ASD) for season of birth, by socioeconomic status (SES)

|  | SES median-high | | | | SES median - low | | | |
| --- | --- | --- | --- | --- | --- | --- | --- | --- |
| **Season of birth** | **OR crude** | **95%**  **CI** | **OR adjusted** | **95%**  **CI** | **OR crude** | **95% CI** | **OR adjusted** | **95% CI** |
| Winter | 1 (ref.) |  | 1 (ref.) |  | 1 (ref.) |  | 1 (ref.) | 1 (ref.) |
| Spring | 1.12 | 0.84 1.50 | 1.12 | 0.84 1.50 | 1.06 | 0.83 1.34 | 1.06 | 0.83 1.34 |
| Summer | 1.24 | 0.94 1.63 | 1.23 | 0.93 1.63 | 1.08 | 0.85 1.37 | 1.08 | 0.85 1.37 |
| Fall | 1.14 | 0.86 1.51 | 1.14 | 0.86 1.51 | 1.05 | 0.82 1.33 | 1.04 | 0.82 1.32 |

*Adjusted for child's age, father’s age, mother’s age, district, birth weight, gestational age with imputation.

Table d: Crude and adjusted odd ratio (OR) and 95% confidence interval from multivariable logistic regression of autistic spectrum disorder (ASD) for season of conception, by socioeconomic status (SES)

|  | SES median-high | | | | | | SES median-low | | | | | |
| --- | --- | --- | --- | --- | --- | --- | --- | --- | --- | --- | --- | --- |
| **Season of conception** | **OR crude** | **95%CI** | | **OR** | **95%CI** | | **OR crude** | **95%CI** | | **OR** | **95%CI** | |
|  |  |  |  | **adjusted*** |  |  |  |  |  | **adjusted*** |  |  |
| **Winter** | 1 (ref.) |  |  | 1 (ref. |  | | 1 (ref.) |  |  | 1 (ref. |  | |
| **Spring** | 1.00 | 0.75 | 1.34 | 0.99 | 0.74 | 1.33 | 0.96 | 0.75 | 1.22 | 0.96 | 0.75 | 1.23 |
| **Summer** | 1.00 | 0.74 | 1.35 | 0.99 | 0.73 | 1.33 | 0.96 | 0.75 | 1.22 | 0.97 | 0.76 | 1.24 |
| **Fall** | 1.09 | 0.82 | 1.45 | 1.08 | 0.82 | 1.44 | 1.05 | 0.83 | 1.33 | 1.06 | 0.83 | 1.34 |

*Adjusted for child's age, father’s age, mother’s age, district, birth weight, gestational age with imputation

Table e: Crude and adjusted odd ratio (OR) and 95% confidence interval from multivariable logistic regression of autistic spectrum disorder (ASD) for month of conception, by socioeconomic status (SES)

|  | SES 1-6 | | | | | | SES 7-10 | | | | | |
| --- | --- | --- | --- | --- | --- | --- | --- | --- | --- | --- | --- | --- |
| **Month of conception** | **OR crude** | **95%CI** | | **OR** | **95%CI** | | **OR crude** | **95%CI** | | **OR** | **95%CI** | |
|  |  |  |  | **adjusted*** |  |  |  |  |  | **adjusted*** |  |  |
| Jan | 1 (ref.) |  |  | 1 (ref. |  | | 1 (ref.) |  |  | 1 (ref. |  | |
| Feb | .89 | .52 | 1.50 | .89 | .52 | 1.51 | 1.09 | .72 | 1.65 | 1.08 | .72 | 1.63 |
| Mar | 1.13 | .70 | 1.85 | 1.14 | .70 | 1.86 | .84 | .55 | 1.30 | .84 | .54 | 1.29 |
| Apr | .90 | .53 | 1.53 | .89 | .53 | 1.52 | 1.06 | .71 | 1.60 | 1.09 | .72 | 1.64 |
| May | 1.05 | .63 | 1.75 | 1.03 | .62 | 1.71 | .94 | .62 | 1.44 | .95 | .62 | 1.45 |
| Jun | 1.19 | .72 | 1.96 | 1.17 | .71 | 1.93 | .74 | .46 | 1.16 | .75 | .47 | 1.18 |
| Jul | .79 | .45 | 1.38 | .79 | .45 | 1.38 | .96 | .63 | 1.45 | .97 | .64 | 1.47 |
| Aug | 1.11 | .67 | 1.83 | 1.09 | .66 | 1.81 | 1.13 | .76 | 1.68 | 1.13 | .76 | 1.68 |
| Sep | 1.29 | .79 | 2.10 | 1.28 | .78 | 2.08 | 1.09 | .73 | 1.64 | 1.10 | .73 | 1.65 |
| Oct | .95 | .57 | 1.58 | .96 | .58 | 1.59 | .88 | .58 | 1.34 | .89 | .58 | 1.35 |
| Nov | 1.14 | .70 | 1.86 | 1.14 | .70 | 1.86 | 1.16 | .78 | 1.72 | 1.16 | .78 | 1.72 |
| Dec | 1.18 | .74 | 1.91 | 1.18 | .74 | 1.91 | .90 | .59 | 1.36 | .89 | .59 | 1.36 |

*Adjusted for child's age, father’s age, mother’s age, district, birth weight, age with imputation (see text for details)
