# Supplementary material for: Month of birth and risk of autism spectrum disorder: a retrospective cohort of male children born in Israel
Source: BMJ Open. 2017 Nov 16;7(11):e014606. doi: 10.1136/bmjopen-2016-014606 (PMC5702026; doi:10.1136/bmjopen-2016-014606)
Supplement: Author's manuscript [file bmjopen-2016-014606.draft_revisions.pdf]

BMJ Open

Month of birth and risk of autism spectrum disorder

Journal:	*BMJ Open*
Manuscript ID	bmjopen-2016-014606
Article Type:	Research
Date Submitted by the Author:	13-Oct-2016
Complete List of Authors:	Shalev, Hila; Technion Israel Institute of Technology Ruth and Bruce Rappaport Faculty of Medicine, Medicine<br>Chodick, Gabriel ; Sackler School of Medicine Tel Aviv University, Ramat Aviv, Israel, Epidemiology and Database Research<br>Solt, Ido; Rambam Health Care Campus; Technion Israel Institute of Technology Ruth and Bruce Rappaport Faculty of Medicine, Medicine
**Primary Subject Heading**:	Epidemiology
Secondary Subject Heading:	Paediatrics, Public health
Keywords:	EPIDEMIOLOGY, Paediatric neurology < NEUROLOGY, Maternal medicine < OBSTETRICS, Developmental neurology & neurodisability < PAEDIATRICS

SCHOLARONE™
Manuscripts

For peer review only

Month of birth and risk of autism spectrum disorder

Hila Shalev BMD[1], Ido Solt MD[4,5], Gabriel Chodick PhD[2,3]

[1] Faculty of Medicine, Technion, Haifa, Israel

[2] Maccabi Healthcare Services, Tel Aviv, Israel,

[3] Sackler Faculty of Medicine, Tel Aviv University, Tel Aviv, Israel

[4] Rambam Health Care campus

[5] The Rappaport Faculty of Medicine, Technion

Corresponding author:

Gabriel Chodick PhD

Epidemiology & Database Research

Medical Division, Maccabi Healthcare Services, Tel-Aviv, Israel

Email. hodik_g@mac.org.il

Tel: 972.3795.2430

School of Public Health,

Faculty of Medicine,

Tel-Aviv Univerity

Tel-Aviv Israel

For peer review only

Abstract

Background: Increased incidence and prevalence of autism spectrum disorder (ASD) over the last two decades have prompted considerable efforts to investigate its etiological factors. We examined an association between month of birth and ASD incidence.

Methods: In a retrospective cohort of male children born from January 1999 to December 2008 in a large health organization in Israel (Maccabi Healthcare Services), ASD was followed from birth through December 2015.

Results: Of 108,548 boys, 975 cases of ASD were identified. The highest rates (1 per 1000 male live births) were recorded for children born in May and August, and the lowest rates for February (7.6 per 1000 male live births). Among lower socioeconomic status households, boys born in August were significantly more likely (OR=1.71; 95% confidence interval: 1.06 – 2.74) of being diagnosed with ASD than children born in January. Significantly higher rates were not observed for other months.

Conclusions: In a lower socioeconomic population, male children born in August were at increased likelihood of being diagnosed with ASD. This may suggest the involvement of a seasonal environmental factor in ASD development, particularly among lower socioeconomic populations.

For peer review only

Strengths of the current study include:

- Large and unselected population
- Cohort design
- The use of systematic data that were collected prospectively
- Validated source of information of ASD diagnosis
- Objective assessment of socioeconomic status.

Limitations of the study:

- We had no information about the ASD disease severity
- Data were missing in some cases for gestational week at birth
- Only males were included, contrasting with other studies that included both genders
- The information was lacking about families history and lack of information in our population on influenza infections and vaccines.
- SES assessment was conducted using data on current residential area, which is not necessarily the residential area during the time of pregnancy, nor necessarily identical to individual SES
- The children who were born at the end of the year were younger than those born earlier in the year, but this is negligible because they were all at the age of at least six years when the study was done and most autistic children are diagnosed by this age [48].

However, these limitations do not seem to be relevant to seasonality, and thus would not be expected to affect study findings.

For peer review only

Autism spectrum disorder (ASD) is a biologically-based neurodevelopmental condition characterized by a) deficits in social communication and social interaction, and b) restricted repetitive patterns of behavior, interests, and activities [1]. The prevalence of ASD has been rising [2] globally, and in Israel in particular [3].

Analyses of season of birth or conception can provide clues about causes of diseases with unknown etiology. Patterns of seasonal variation in births have been found in several disorders including schizophrenia, mood disorders, epilepsy, language disorders, attention deficit hyperactivity disorder (ADHD) and neurodevelopmental disabilities[4-9]. An association between season of birth or conception suggests periodicity of an environmental etiologic factor active during the prenatal, perinatal or early postnatal phase of development [10]. Seasonal effects observed in these disorders (especially in schizophrenia) prompted several researchers to consider whether the same would apply to autism spectrum disorders (ASD). The findings of such investigations are inconsistent. Inconsistencies in the literature could be due, in part, to a wide range of study populations, geographical areas, case definitions, comparison groups and analytical approaches

The objective of the present study was to examine a relationship between autism and seasonality in a large and unselected population in Israel. Previous Israeli studies [11] [12] reported conflicting results and included relatively small numbers of ASD cases. The male-to-female ratio among ASD patients is reported between 3:1 and 4:1 [13] [14] [15], reflecting a disorder of sexual differentiation and potential differences in mechanisms and etiology between the two sexes [16] . Therefore we limited this analysis to males only.

For peer review only - http://bmjopen.bmj.com/site/about/guidelines.xhtml

Methods

Settings

The 1994 National Health Insurance law mandates compulsory universal health coverage for all Israeli residents in a health maintenance organization (HMO) of their choice. Residents can choose one of four non-for profit HMOs operating across the country. Patient fees are equivalent across the four HMOs, and all provide equivalent medical services that are based on national health regulations. For this review, we examined data on ASD diagnosis from the computer database of the second-largest HMO, Maccabi Healthcare Services (MHS), which provides care to 2 million people (31% under 18 years of age) (Israeli Central Bureau of Statistics).

Study population

Using the computerized databases of MHS, we identified a total of 147,171 male children who were born between January 1999 and December 2008 (approximately 20% of all 736 thousand live male births in Israel during the same years). Children's parents were identified using data on household members in MHS. Children with missing paternal data (mostly due to previously belonging to a different health provider) were excluded from the study. Comparison of excluded (n=38,875) and eligible (n=110,093) children showed no statistically significant (P>0.05) differences in mean birth weight (3271 gram and 3315 gram, respectively), mean gestational week (39.0 and 39.1), or mother's age at birth (29.4 and 30.5). To avoid inaccurate paternal identification, an additional 1,545 children were excluded because more than one man was registered in the child's household. Hence, the total study population was 108,548 boys of 83,452 households.

Case identification and matching

A case was defined as a child with a physician–recorded diagnosis of autistic disorder, Asperger's syndrome, or PDD-NOS, based on the DSM-IV (American Psychiatric Association 1994). In MHS, children with suspected ASD are referred to child development centers for a multidisciplinary assessment for diagnosis validation. MHS' registry of ASD patients and validation processes were previously described[17].

For peer review only - http://bmjopen.bmj.com/site/about/guidelines.xhtml

Other study variables

Socioeconomic status (SES) was assessed according to the poverty index of the child's address enumeration area, as defined by 2008 national census[17] . The poverty index is based on several parameters including household income, educational qualifications, crowding, material conditions, and car ownership, census of population and housing [18]. SES levels range between 1 (lowest) to 10 (highest). In this study SES below median (6) was considered as low SES.

Statistical Analyses

First, bivariate analyses were conducted between month of birth and other covariates previously reported to be associated with increased risk of autism [19 20]. The covariates included preterm birth (defined as gestational age <37 weeks), birth weight, older maternal or paternal age (>35 years), and SES level above median (>6).

Multivariable logistic regression analyses were performed to determine the odds ratio (OR) and 95% confidence interval CI for ASD by month of birth, using January as the reference month. For the purpose of comparison with previous studies, we fitted alternative logistic regression models, with month of conception (rather than birth) as the main independent variable. The Cochran-Armitage test was used to assess a significant trend in the association between month of birth and ASD. A two-tailed P value of <0.05 was considered statistically significant. All data processing and statistical analyses were performed using IBM Corp. Released 2013. IBM SPSS Statistics for Windows, Version 22.0. Armonk, NY: IBM Corp.).

Results

The mean number of monthly births ranged from 8318 in February to 9654 in August. In bivariate analyses, number of births, maternal age, paternal age, birth weight, gestational length and SES were not associated with the month of birth (Table 1). Of the 108,548 boys born during the study period, 975 were diagnosed with ASD, representing a prevalence rate of 9.0 per 1000 live births. Mean age at ASD diagnosis was 3.0 years (SE=0.07years). The highest incidences of ASD were recorded among children born in

For peer review only - http://bmjopen.bmj.com/site/about/guidelines.xhtml

May and August: 10.3 and 10.2 per 1000, respectively, while the lowest incidences were in January and February (7.6 and 8.1 per 1000, respectively) (Table 2).

In a multivariable model, birth months of August and May were associated with a non-significantly (P=0.16 and P=0.14, respectively) increased odds ratio (OR) (Table 3). When the data were stratified by household socioeconomic level, the birth month of August in low SES was associated with an odds ratio of 1.71 (95% confidence interval: 1.06 – 2.74) of ASD. No other significant results were observed for other birth months or in children of higher SES (Figure I)

Discussion

In this retrospective cohort of males born in Israel during a period spanning ten years, children of low SES born in the month of August were at a modestly increased risk of being diagnosed with ASD. This is the one of the largest studies outside the US to describe the relationship between ASD incidence and month of birth.

The literature with respect to season of birth and risk of ASD is inconsistent. Of the 15 studies summarized in table 4 (all conducted in the northern hemisphere), comprising a total number of 31,777 cases, five studies (total n=23,355) reported an increased risk of ASD of individuals born in August. March was significantly associated with increased risk in eight studies (total n=3008), and three studies (total n=5839) found no significant association between month of birth and ASD occurrence. No other specific birth month was repeatedly reported to be associated with ASD. Birth in August was previously reported to be associated with increased risk of ASD in an Israeli study that comprised 188 cases[11],

There are several mechanisms by which season of birth may affect the developing brain, either directly on the fetus and newborn or indirectly through the mother during pregnancy or the postpartum period. Seasonal environmental factors that could be associated with ASD include viral or other infections[21, 22], nutritional factors[23], and vitamin deficiencies[24]. The outcome of exposure to prenatal viral infection depends on many factors: the variation in susceptibility of the maternal and fetal host, including the maternal immune status, the infecting virus, the strain of virus, the developmental stage of the fetus, the amount of virus reaching the fetus— particularly the CNS and immune system, the route of access, genetics, and probably other factors[25]. Maternal viral infection has been cited as the “principal non-genetic cause of autism” [26]. Maternal

For peer review only - http://bmjopen.bmj.com/site/about/guidelines.xhtml

infections (i.e. rubella, cytomegalovirus, influenza), prolonged fever and maternal inflammation cause changes in a variety of inflammatory cytokines that have been associated with ASD [27, 28]. Depending on the location of immune activation, persistent viral infection could lead to chronically elevated cytokine levels that can be produced directly in the brain or gain access to the CNS by crossing an immature blood-brain barrier (BBB), altering CNS development[29].

A few studies found influenza infection during pregnancy to be a factor that increases risk for infantile autism[21 30] especially in the first trimester [28], also on animal models[31]. We do not have data regarding influenza infections or influenza vaccines in our population. However, According to the Israeli Center for Disease Control[32], January and then February are the months with the highest percentage of influenza cases (nearly 85% of all cases). These are the critical months during which time CNS develops in children born in August. The CHARGE (CHildhood Autism Risks from Genetics and Environment) study found that fewer mothers of children with ASD reported receiving influenza vaccine than mothers of typical controls [33]. Moreover, lower SES has been found to be correlated with lower flu shot uptake among persons aged19–49 years [34-36]. This increases the likelihood of influenza during the first trimester. There is also evidence that low socioeconomic populations tend to be more hospitalized for influenza infections [37 38] and epidemiological studies found increased risk of ASD following maternal hospitalization due to infection [39 38]. The association between uptake of influenza vaccines in pregnancy and the risk of ASD in newborns warrants further investigation.

A role of vitamin D deficiency in the development of autism has been proposed [24]. Thus, seasonal fluctuations in vitamin D should also be considered. In the northern hemisphere, vitamin D levels are the lowest in winter time [40-42] ; however, a study of healthy volunteers from Israel found no differences in vitamin D deficiency rates between summer and winter. IMAJ 2010; 12: 751–756

In the current study, the association between month of birth and ASD risk was significant only among individuals of lower SES level. Low socioeconomic status has been previously related[43] to increased risk to exposure to damaging agents in the environment, including lead[44], asbestos, carbon dioxide, and industrial waste and pollutants[45 46 47]. A study that examined prenatal exposure to 35 hazardous air pollutants, found an association of exposure to pollutants including methylene chloride

For peer review only - http://bmjopen.bmj.com/site/about/guidelines.xhtml

(OR 1.4: 95% CI, 0.7-2.5), quinolone (OR, 1.4; 95% CI 1.0-2.2) and styrene (OR, 1.8; 95% CI, 1.0-3.1), with a higher risk for ASD.

Strengths of the current study include the large and unselected population, cohort design, the use of systematic data that were collected prospectively, validated source of information of ASD diagnosis, and objective assessment of socioeconomic status. Some limitations should be discussed. We had no information about the ASD disease severity; data were missing in some cases for gestational week at birth; only males were included, contrasting with other studies that included both genders; the information was lacking about families history and lack of information in our population on influenza infections and vaccines. However, these limitations do not seem to be relevant to seasonality, and thus would not be expected to affect study findings. In addition, SES assessment was conducted using data on current residential area, which is not necessarily the residential area during the time of pregnancy, nor necessarily identical to individual SES. The children who were born at the end of the year were younger than those born earlier in the year, but this is negligible because they were all at the age of at least six years when the study was done and most autistic children are diagnosed by this age [48].

CONCLUSIONS

We found a significant association between birth in August and ASD among children of lower SES levels. Increased risk of ASD among children born in August (or March) has been reported in several large cohorts, suggesting a true seasonal pattern. A deeper understanding of these findings with regard to the various and complex etiologic pathways leading to development of ASD will be of value to better understanding this disorder.

CONTRIBUTIONS OF AUTHORS:

HS and GC contributed substantially to the conception of the work, analysis, interpretation of data for the work, drafting the work and final approval of the version to be published. IS contributed substantially to the acquisition, interpretation of data for the work, revising it critically for important intellectual content and final approval of the version to be published.

For peer review only - http://bmjopen.bmj.com/site/about/guidelines.xhtml

COMPETING INTERESTS: None Declared.

FUNDING: None.

DATA SHARING STATEMENT: No additional data are available

ETHICS APPROVAL: This study was approved by Assuta Healthcare Systems IRB.

ACKNOWLEDGMENTS

This study is part of the MD thesis by Hila Shalev

For peer review only - http://bmjopen.bmj.com/site/about/guidelines.xhtml

Refrences

1. Autism spectrum disorder. In: Diagnostic and Statistical Manual of Mental Disorders. 2013.
2. Newschaffer CJ, Croen LA, Daniels J, Giarelli E, Grether JK, Levy SE, Mandell DS, Miller LA, Pinto-Martin J, Reaven J, Reynolds AM, Rice CE, Schendel D and Windham GC. The epidemiology of autism spectrum disorders. *Annu Rev Public Health*. 2007;28:235-58.
3. Senecky Y, Chodick G, Diamond G, Lobel D, Drachman R and Inbar D. Time trends in reported autistic spectrum disorders in Israel, 1972-2004. *Isr Med Assoc J*. 2009;11:30-3.
4. Torrey EF, Miller J, Rawlings R and Yolken RH. Seasonal birth patterns of neurological disorders. *Neuroepidemiology*. 2000;19:177-85.
5. Mortensen PB, Pedersen CB, Westergaard T, Wohlfahrt J, Ewald H, Mors O, Andersen PK and Melbye M. Effects of family history and place and season of birth on the risk of schizophrenia. *N Engl J Med*. 1999;340:603-8.
6. Torrey EF, Miller J, Rawlings R and Yolken RH. Seasonality of births in schizophrenia and bipolar disorder: a review of the literature. *Schizophr Res*. 1997;28:1-38.
7. Mick E, Biederman J and Faraone SV. Is season of birth a risk factor for attention-deficit hyperactivity disorder? *J Am Acad Child Adolesc Psychiatry*. 1996;35:1470-6.
8. Liederman J and Flannery KA. Fall conception increases the risk of neurodevelopmental disorder in offspring. *J Clin Exp Neuropsychol*. 1994;16:754-68.
9. Livingston R, Adam BS and Bracha HS. Season of birth and neurodevelopmental disorders: summer birth is associated with dyslexia. *J Am Acad Child Adolesc Psychiatry*. 1993;32:612-6.
10. Castrogiovanni P, Iapichino S, Pacchierotti C and Pieraccini F. Season of birth in psychiatry. A review. *Neuropsychobiology*. 1998;37:175-81.
11. Barak Y, Ring A, Sulkes J, Gabbay U and Elizur A. Season of birth and autistic disorder in Israel. *Am J Psychiatry*. 1995;152:798-800.
12. Kolevzon A, Weiser M, Gross R, Lubin G, Knobler HY, Schmeidler J, Silverman JM and Reichenberg A. Effects of season of birth on autism spectrum disorders: fact or fiction? *Am J Psychiatry*. 2006;163:1288-90.
13. Biederman J. Attention-deficit/hyperactivity disorder: a selective overview. *Biol Psychiatry*. 2005;57:1215-20.
14. Biederman J and Faraone SV. Attention-deficit hyperactivity disorder. *Lancet*. 2005;366:237-48.
15. Rappley MD. Clinical practice. Attention deficit-hyperactivity disorder. *N Engl J Med*. 2005;352:165-73.
16. Alaerts K, Swinnen SP and Wenderoth N. Sex differences in autism: a resting-state fMRI investigation of functional brain connectivity in males and females. *Soc Cogn Affect Neurosci*. 2016.
17. Davidovitch M, Hemo B, Manning-Courtney P and Fombonne E. Prevalence and incidence of autism spectrum disorder in an Israeli population. *J Autism Dev Disord*. 2013;43:785-93.
18. *Israel Central Bureau of Statistics*; 2011.
19. Roberts EM, English PB, Grether JK, Windham GC, Somberg L and Wolff C. Maternal residence near agricultural pesticide applications and autism spectrum

For peer review only - http://bmjopen.bmj.com/site/about/guidelines.xhtml

disorders among children in the California Central Valley. *Environ Health Perspect*. 2007;115:1482-9.
20. Croen LA, Grether JK and Selvin S. Descriptive epidemiology of autism in a California population: who is at risk? *J Autism Dev Disord*. 2002;32:217-24.
21. Deykin EY and MacMahon B. Viral exposure and autism. *Am J Epidemiol*. 1979;109:628-38.
22. Lee BK, Magnusson C, Gardner RM, Blomström Å, Newschaffer CJ, Burstyn I, Karlsson H and Dalman C. Maternal hospitalization with infection during pregnancy and risk of autism spectrum disorders. *Brain Behav Immun*. 2015;44:100-5.
23. Lyall K, Schmidt RJ and Hertz-Picciotto I. Maternal lifestyle and environmental risk factors for autism spectrum disorders. *Int J Epidemiol*. 2014;43:443-64.
24. Grant WB and Soles CM. Epidemiologic evidence supporting the role of maternal vitamin D deficiency as a risk factor for the development of infantile autism. *Dermatoendocrinol*. 2009;1:223-8.
25. Blattner RJ. The role of viruses in congenital defects. *Am J Dis Child*. 1974;128:781-6.
26. Ciaranello AL and Ciaranello RD. The neurobiology of infantile autism. *Annu Rev Neurosci*. 1995;18:101-28.
27. Ornoy A, Weinstein-Fudim L and Ergaz Z. Prenatal factors associated with autism spectrum disorder (ASD). *Reprod Toxicol*. 2015;56:155-69.
28. Atladóttir H, Henriksen TB, Schendel DE and Parner ET. Autism after infection, febrile episodes, and antibiotic use during pregnancy: an exploratory study. *Pediatrics*. 2012;130:e1447-54.
29. Muñoz-Fernández MA and Fresno M. The role of tumour necrosis factor, interleukin 6, interferon-gamma and inducible nitric oxide synthase in the development and pathology of the nervous system. *Prog Neurobiol*. 1998;56:307-40.
30. Zhang X, Lv CC, Tian J, Miao RJ, Xi W, Hertz-Picciotto I and Qi L. Prenatal and perinatal risk factors for autism in China. *J Autism Dev Disord*. 2010;40:1311-21.
31. Shi L, Fatemi SH, Sidwell RW and Patterson PH. Maternal influenza infection causes marked behavioral and pharmacological changes in the offspring. *J Neurosci*. 2003;23:297-302.
32. Israeli Center for Disease Control.
33. Zerbo O, Iosif AM, Walker C, Ozonoff S, Hansen RL and Hertz-Picciotto I. Is maternal influenza or fever during pregnancy associated with autism or developmental delays? Results from the CHARGE (CHildhood Autism Risks from Genetics and Environment) study. *J Autism Dev Disord*. 2013;43:25-33.
34. Peña-Rey I, Pérez-Farinós N and Sarría-Santamera A. Factors associated with influenza vaccination among elderly Spanish women. *Public Health*. 2004;118:582-7.
35. Lee KC, Han K, Kim JY, Nam GE, Han BD, Shin KE, Lee A and Ko BJ. Socioeconomic status and other related factors of seasonal influenza vaccination in the South Korean adult population based on a nationwide cross-sectional study. *PLoS One*. 2015;10:e0117305.
36. Damiani G, Federico B, Visca M, Agostini F and Ricciardi W. The impact of socioeconomic level on influenza vaccination among Italian adults and elderly: a cross-sectional study. *Prev Med*. 2007;45:373-9.
37. Tam K, Yousey-Hindes K and Hadler JL. Influenza-related hospitalization of adults associated with low census tract socioeconomic status and female sex in New Haven County, Connecticut, 2007-2011. *Influenza Other Respir Viruses*. 2014;8:274-81.

For peer review only - http://bmjopen.bmj.com/site/about/guidelines.xhtml

38. Sloan C, Chandrasekhar R, Mitchel E, Schaffner W and Lindegren ML. Socioeconomic Disparities and Influenza Hospitalizations, Tennessee, USA. *Emerg Infect Dis*. 2015;21:1602-10.
39. Atladóttir HO, Thorsen P, Østergaard L, Schendel DE, Lemcke S, Abdallah M and Parner ET. Maternal infection requiring hospitalization during pregnancy and autism spectrum disorders. *J Autism Dev Disord*. 2010;40:1423-30.
40. Kasahara AK, Singh RJ and Noymer A. Vitamin D (25OHD) Serum Seasonality in the United States. *PLoS One*. 2013;8:e65785.
41. Kashi Z, Saeedian F, Akha O, Gorgi M, Emadi S and Zakeri H. Vitamin D deficiency prevalence in summer compared to winter in a city with high humidity and a sultry climate. *Endokrynol Pol*. 2011;62:249-51.
42. Costanzo PR, Constanzo PR, Elías NO, Kleiman Rubinsztein J, García Basavilbaso NX, Piacentini R and Salerni HH. [Ultraviolet radiation impact on seasonal variations of serum 25-hydroxy-vitamin D in healthy young adults in Buenos Aires]. *Medicina (B Aires)*. 2011;71:336-42.
43. Pamuk E. Status and Health Chartbook 1998.
44. Pocock SJ SM, Baghurst P. Environmental lead and children's intelligence: A systematic review of the epidemiological evidence. 1994;309:1189–97.
45. Kalkbrenner AE, Daniels JL, Chen JC, Poole C, Emch M and Morrissey J. Perinatal exposure to hazardous air pollutants and autism spectrum disorders at age 8. *Epidemiology*. 2010;21:631-41.
46. Volk HE, Hertz-Picciotto I, Delwiche L, Lurmann F and McConnell R. Residential proximity to freeways and autism in the CHARGE study. *Environ Health Perspect*. 2011;119:873-7.
47. Becerra TA, Wilhelm M, Olsen J, Cockburn M and Ritz B. Ambient air pollution and autism in Los Angeles county, California. *Environ Health Perspect*. 2013;121:380-6.
48. Hertz-Picciotto I and Delwiche L. The rise in autism and the role of age at diagnosis. *Epidemiology*. 2009;20:84-90.
49. Zerbo O, Iosif AM, Delwiche L, Walker C and Hertz-Picciotto I. Month of conception and risk of autism. *Epidemiology*. 2011;22:469-75.
50. Mazumdar S, Liu KY, Susser E and Bearman P. The disappearing seasonality of autism conceptions in California. *PLoS One*. 2012;7:e41265.
51. Bolton P, Pickles A, Harrington R, Macdonald H and Rutter M. Season of birth: issues, approaches and findings for autism. *J Child Psychol Psychiatry*. 1992;33:509-30.
52. Lee LC, Newschaffer CJ, Lessler JT, Lee BK, Shah R and Zimmerman AW. Variation in season of birth in singleton and multiple births concordant for autism spectrum disorders. *Paediatr Perinat Epidemiol*. 2008;22:172-9.
53. Bartlik BD. Monthly variation in births of autistic children in North Carolina. *J Am Med Womens Assoc*. 1981;36:363-8.
54. Landau EC, Cicchetti DV, Klin A and Volkmar FR. Season of birth in autism: a fiction revisited. *J Autism Dev Disord*. 1999;29:385-93.
55. Mouridsen SE, Nielsen S, Rich B and Isager T. Season of birth in infantile autism and other types of childhood psychoses. *Child Psychiatry Hum Dev*. 1994;25:31-43.
56. Konstantareas MM, Hauser P, Lennox C and Homatidis S. Season of birth in infantile autism. *Child Psychiatry Hum Dev*. 1986;17:53-65.
57. Gillberg C. Do children with autism have March birthdays? *Acta Psychiatr Scand*. 1990;82:152-6.

For peer review only - http://bmjopen.bmj.com/site/about/guidelines.xhtml

58. Tanoue Y, Oda S, Asano F and Kawashima K. Epidemiology of infantile autism in southern Ibaraki, Japan: differences in prevalence in birth cohorts. *J Autism Dev Disord*. 1988;18:155-66.
59. Hebert KJ, Miller LL and Joinson CJ. Association of autistic spectrum disorder with season of birth and conception in a UK cohort. *Autism Res*. 2010;3:185-90.

For peer review only

For peer review only - http://bmjopen.bmj.com/site/about/guidelines.xhtml

Table 1 Study population characteristics by month of birth

**Month**	***Births***	**Maternal age Mean (SD)**	**Paternal a[illegible] Mean (SD)**	**Birth weigh[illegible] Mean (SD)**	**Gestationa[illegible] week (SD)**	**SES* 1 to 20 Mean (SD)**
Jan	*9367*	30.1 (4.9)	32.9 (5.7)	3296 (525)	39.1 (1.9)	6.1 (2.3)
Feb	*8318*	30.2 (4.9)	32.9 (5.7)	3296 (528)	39.0 (2.0)	6.2 (2.3)
Mar	*9038*	30.2 (4.9)	32.9 (5.6)	3305 (519)	39.0 (1.9)	6.1 (2.3)
Apr	*8465*	30.3 (4.9)	33.0 (5.6)	3310 (519)	39.1 (1.9)	6.2 (2.3)
May	*8711*	30.3 (4.9)	33.0 (5.6)	3300 (532)	39.1 (1.9)	6.3 (2.3)
Jun	*8523*	30.3 (5.0)	33.1 (5.6)	3306 (538)	39.0 (2.0)	6.2 (2.3)
Jul	*9330*	30.2 (4.9)	33.0 (5.7)	3304 (528)	39.0 (2.0)	6.1 (2.3)
Aug	*9654*	30.3 (4.9)	33.1 (5.7)	3312 (532)	39.0 (1.9)	6.1 (2.3)
Sep	*9270*	30.3 (4.9)	33.1 (5.7)	3291 (537)	39.0 (1.9)	6.1 (2.3)
Oct	*9521*	30.3 (5.0)	33.0 (5.7)	3302 (524)	39.1 (1.9)	6.1 (2.3)
Nov	*9069*	30.3 (5.0)	33.1 (5.7)	3302 (532)	39.0 (1.9)	6.1 (2.3)
Dec	*9282*	30.3 (5.0)	33.0 (5.7)	3288 (540)	39.0 (2.0)	6.1 (2.3)
**Total**	***108548***	**30.3 (5.0)**	**33.0 (5.7)**	**3301 (529.5**	**39.0 (2.0)**	**6.1 (2.3)**

- See text for details on definitions of SES

For peer review only - http://bmjopen.bmj.com/site/about/guidelines.xhtml

Table 2: Incidence rate and 95% confidence intervals (95%CI) per 1000 male live births of Autistic spectrum disorder (ASD), by month of birth

Month of birth	ASD cases	Rate (per 1000)	95% CI*	
Jan	76	8.1	6.44	10.09
Feb	63	7.6	5.87	9.62
Mar	74	8.2	6.48	10.21
Apr	74	8.7	6.92	10.90
May	90	10.3	8.36	12.62
Jun	78	9.2	7.29	11.35
Jul	89	9.5	7.71	11.67
Aug	98	10.2	8.29	12.30
Sep	80	8.6	6.89	10.67
Oct	85	8.9	7.18	10.97
Nov	84	9.3	7.44	11.40
Dec	84	9.0	7.27	11.14
Total	975	8.96	7.17	11.07

* see text for details

For peer review only

Table 3: Crude and adjusted odd ratio (OR) and 95% confidence interval of autistic spectrum disorder (ASD) for month of birth: multivariable logistic regression

**Month of birth**	**OR crude**	**95%CI**		**OR adjusted***	**95%CI**	
Jan	1 (ref.)			1 (ref.		
Feb	.933	.667	1.305	.923	.660	1.290
Mar	1.009	.732	1.392	1.004	.728	1.385
Apr	1.080	.783	1.487	1.062	.770	1.465
May	1.275	.938	1.734	1.244	.915	1.691
Jun	1.128	.822	1.551	1.104	.804	1.517
Jul	1.164	.855	1.601	1.154	.847	1.570
Aug	1.253	.928	1.694	1.237	.915	1.671
Sep	1.066	.777	1.459	1.054	.769	1.445
Oct	1.102	.808	1.503	1.097	.803	1.497
Nov	1.132	.828	1.561	1.123	.822	1.536
Dec	1.105	.808	1.525	1.097	.802	1.500

*Adjusted for child's age, father's age, mother's age, district, birth weight, gestational length.

For peer review only

For peer review only - http://bmjopen.bmj.com/site/about/guidelines.xhtml

Table 4: Summary of Findings in Previous Studies Investigating the Relationship Between Seasonal Variations of Birth and Autism

Ref.	Study period	**Place**	ASD cases	Months of increased risk of ASD
49	1990 – 2002	California	19,238	Aug, Sep, Oct, Nov, Dec
50	1992 – 2000	California	2,940	Aug*
50	1997 – 1999	California	3,897	No Association
51	1947 – 1980	UK	1631	No Association
52	1983 – 2002	Maryland	1068	Spring
53	1981***	N Carolina	810	Mar, Aug
54	1947-1992	Child dev. clinic	620	No Association**
55	1945 – 1980	Denmark	328	March
12	1980 – 1985	Israel	311	Association No
11	1964 – 1986	Israel	188	March and August
56	1980s	Ottario, Canada	179	Summer and Spring
	1977 – 1985	US	175	March
57	1962 – 1984	Sweden	174	March
58	1972 – 1978	Japan	132	Spring-Summer (Apr, May, Jun)
59	1990–1992	UK	86	Spring

- Conception in November (For years 1994 – 1996) ** (P<0.1 for middle of August for verbal ASD) *** year of publication

For peer review only

Figure I: Odds ratios and 95% confidence intervals of ASD incidence according to months of birth, relative to the month of January, by household socioeconomic status

For peer review only

For peer review only - http://bmjopen.bmj.com/site/about/guidelines.xhtml

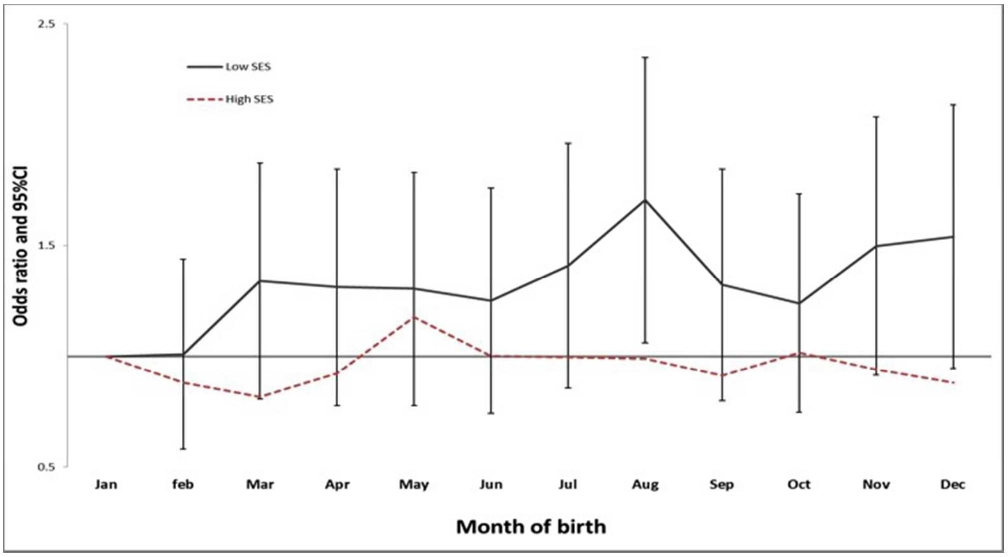

Odds ratios and 95% confidence intervals of ASD incidence according to months of birth, relative to the month of January, by household socioeconomic status

259x142mm (72 x 72 DPI)

review only

STROBE 2007 (v4) checklist of items to be included in reports of observational studies in epidemiology*

Checklist for cohort, case-control, and cross-sectional studies (combined)

Section/Topic	Item #	Recommendation	Reported on page #
**Title and abstract**	1	(*a*) Indicate the study's design with a commonly used term in the title or the abstract	1-2
		(*b*) Provide in the abstract an informative and balanced summary of what was done and what was found	1-2
**Introduction**			
Background/rationale	2	Explain the scientific background and rationale for the investigation being reported	4
Objectives	3	State specific objectives, including any pre-specified hypotheses	4
**Methods**			
Study design	4	Present key elements of study design early in the paper	2
Setting	5	Describe the setting, locations, and relevant dates, including periods of recruitment, exposure, follow-up, and data collection	2
Participants	6	(*a*) *Cohort study*—Give the eligibility criteria, and the sources and methods of selection of participants. Describe methods of follow-up<br>*Case-control study*—Give the eligibility criteria, and the sources and methods of case ascertainment and control selection. Give the rationale for the choice of cases and controls<br>*Cross-sectional study*—Give the eligibility criteria, and the sources and methods of selection of participants	5
		(*b*) *Cohort study*—For matched studies, give matching criteria and number of exposed and unexposed<br>*Case-control study*—For matched studies, give matching criteria and the number of controls per case	-
Variables	7	Clearly define all outcomes, exposures, predictors, potential confounders, and effect modifiers. Give diagnostic criteria, if applicable	8
Data sources/ measurement	8*	For each variable of interest, give sources of data and details of methods of assessment (measurement). Describe comparability of assessment methods if there is more than one group	*5*
Bias	9	Describe any efforts to address potential sources of bias	-
Study size	10	Explain how the study size was arrived at	5
Quantitative variables	11	Explain how quantitative variables were handled in the analyses. If applicable, describe which groupings were chosen and why	6
Statistical methods	12	(*a*) Describe all statistical methods, including those used to control for confounding	6
		(*b*) Describe any methods used to examine subgroups and interactions	6
		(*c*) Explain how missing data were addressed	5
		(*d*) *Cohort study*—If applicable, explain how loss to follow-up was addressed<br>*Case-control study*—If applicable, explain how matching of cases and controls was addressed	5

For peer review only

		Cross-sectional study—If applicable, describe analytical methods taking account of sampling strategy	
		(*e*) Describe any sensitivity analyses	-
**Results**			
Participants	13*	(a) Report numbers of individuals at each stage of study—eg numbers potentially eligible, examined for eligibility, confirmed eligible, included in the study, completing follow-up, and analysed	5
		(b) Give reasons for non-participation at each stage	5
		(c) Consider use of a flow diagram	-
Descriptive data	14*	(a) Give characteristics of study participants (eg demographic, clinical, social) and information on exposures and potential confounders	5
		(b) Indicate number of participants with missing data for each variable of interest	5
		(c) *Cohort study*—Summarise follow-up time (eg, average and total amount)	-
Outcome data	15*	*Cohort study*—Report numbers of outcome events or summary measures over time	-
		Case-control study—Report numbers in each exposure category, or summary measures of exposure	5
		Cross-sectional study—Report numbers of outcome events or summary measures	-
Main results	16	(*a*) Give unadjusted estimates and, if applicable, confounder-adjusted estimates and their precision (eg, 95% confidence interval). Make clear which confounders were adjusted for and why they were included	-
		(*b*) Report category boundaries when continuous variables were categorized	-
		(*c*) If relevant, consider translating estimates of relative risk into absolute risk for a meaningful time period	16
Other analyses	17	Report other analyses done—eg analyses of subgroups and interactions, and sensitivity analyses	6
**Discussion**			
Key results	18	Summarise key results with reference to study objectives	7+17
Limitations	19	Discuss limitations of the study, taking into account sources of potential bias or imprecision. Discuss both direction and magnitude of any potential bias	3+8
Interpretation	20	Give a cautious overall interpretation of results considering objectives, limitations, multiplicity of analyses, results from similar studies, and other relevant evidence	7-8
Generalisability	21	Discuss the generalisability (external validity) of the study results	7-8
**Other information**			
Funding	22	Give the source of funding and the role of the funders for the present study and, if applicable, for the original study on which the present article is based	Not Relevant

*Give information separately for cases and controls in case-control studies and, if applicable, for exposed and unexposed groups in cohort and cross-sectional studies.

For peer review only

Note: An Explanation and Elaboration article discusses each checklist item and gives methodological background and published examples of transparent reporting. The STROBE checklist is best used in conjunction with this article (freely available on the Web sites of PLoS Medicine at http://www.plosmedicine.org/, Annals of Internal Medicine at http://www.annals.org/, and Epidemiology at http://www.epidem.com/). Information on the STROBE Initiative is available at www.strobe-statement.org.

For peer review only

BMJ Open

Month of birth and risk of autism spectrum disorder, a retrospective cohort of male children born in Israel

Journal:	*BMJ Open*
Manuscript ID	bmjopen-2016-014606.R1
Article Type:	Research
Date Submitted by the Author:	26-Nov-2016
Complete List of Authors:	Shalev, Hila; Technion Israel Institute of Technology Ruth and Bruce Rappaport Faculty of Medicine, Medicine<br>Chodick, Gabriel ; Sackler School of Medicine Tel Aviv University, Ramat Aviv, Israel, Epidemiology and Database Research<br>Solt, Ido; Rambam Health Care Campus; Technion Israel Institute of Technology Ruth and Bruce Rappaport Faculty of Medicine, Medicine
**Primary Subject Heading**:	Epidemiology
Secondary Subject Heading:	Paediatrics, Public health
Keywords:	EPIDEMIOLOGY, Paediatric neurology < NEUROLOGY, Maternal medicine < OBSTETRICS, Developmental neurology & neurodisability < PAEDIATRICS

SCHOLARONE™
Manuscripts

For peer review only

Month of birth and risk of autism spectrum disorder, a retrospective cohort of male children born in Israel

Hila Shalev BMD[1], Ido Solt MD[4,5], Gabriel Chodick PhD[2,3]

[1] Faculty of Medicine, Technion, Haifa, Israel

[2] Maccabi Healthcare Services, Tel Aviv, Israel,

[3] Sackler Faculty of Medicine, Tel Aviv University, Tel Aviv, Israel

[4] Rambam Health Care campus

[5] The Rappaport Faculty of Medicine, Technion

Corresponding author:

Gabriel Chodick PhD

Epidemiology & Database Research
Medical Division, Maccabi Healthcare
Services, Tel-Aviv, Israel
Email. hodik_g@mac.org.il
Tel: 972.3795.2430

School of Public Health,
Faculty of Medicine,
Tel-Aviv Univerity
Tel-Aviv Israel

For peer review only - http://bmjopen.bmj.com/site/about/guidelines.xhtml

Abstract

Background: Increased incidence and prevalence of autism spectrum disorder (ASD) over the last two decades have prompted considerable efforts to investigate its etiological factors. We examined an association between month of birth and ASD incidence.

Methods: In a retrospective cohort of male children born from January 1999 to December 2008 in a large health organization in Israel (Maccabi Healthcare Services), ASD was followed from birth through December 2015.

Results: Of 108,548 boys, 975 cases of ASD were identified. The highest rates (10.3 and 10.2 per 1000 male live births) were recorded for children born in May and August, and the lowest rates for February (7.6 per 1000 male live births). Among lower socioeconomic status households, boys born in August were significantly more likely (OR=1.71; 95% confidence interval: 1.06 – 2.74) of being diagnosed with ASD than children born in January. Significantly higher rates were not observed for other months.

Conclusions: In a lower socioeconomic population, male children born in August were at increased likelihood of being diagnosed with ASD. This may suggest the involvement of a seasonal environmental factor in ASD development, particularly among lower socioeconomic populations.

For peer review only

For peer review only - http://bmjopen.bmj.com/site/about/guidelines.xhtml

Strengths of the current study include:

- Large population
- Cohort design
- The use of systematic data that were collected prospectively
- Validated source of information of ASD diagnosis
- Objective assessment of socioeconomic status.

Limitations of the study:

- No information was available regardin ASD disease severity
- Data were missing in some cases for gestational week at birth.
- Only males were included, contrasting with other studies that included both sexes.
- Lack of information in our population on influenza infections and vaccines.
- SES was assesed using data on current residential area, which is not necessarily the residential area during the time of pregnancy.

However, these limitations do not seem to be relevant to month of birth, and thus would not be expected to affect study findings.

For peer review only - http://bmjopen.bmj.com/site/about/guidelines.xhtml

Autism spectrum disorder (ASD) is a biologically-based neurodevelopmental condition characterized by a) deficits in social communication and social interaction, and b) restricted repetitive patterns of behavior, interests, and activities [1]. The prevalence of ASD has been rising [2] globally, and in Israel in particular [3].

Analyses of season of birth or conception can provide clues about causes of diseases with unknown etiology. Patterns of seasonal variation in births have been found in several disorders including schizophrenia, mood disorders, epilepsy, language disorders, attention deficit hyperactivity disorder (ADHD) and neurodevelopmental disabilities[4-9]. An association between season of birth or conception suggests periodicity of an environmental etiologic factor active during the prenatal, perinatal or early postnatal phase of development [10]. Seasonal effects observed in these disorders (especially in schizophrenia) prompted several researchers to consider whether the same would apply to autism spectrum disorders (ASD). The findings of such investigations are inconsistent. Inconsistencies in the literature could be due, in part, to a wide range of study populations, geographical areas, case definitions, comparison groups and analytical approaches.

The objective of the present study was to examine a relationship between autism and seasonality in a large population in Israel. Previous Israeli studies [11] [12] reported conflicting results and included relatively small numbers of ASD cases. The male to female ratio among ASD patients is reported between 3 to 1 and 4 to 1 [13, 14] [15], reflecting a disorder of potential differences in mechanisms and etiology between the two sexes[16]. In MHS, the male-to-female ratio among ASD patients is 5:1 [17] which would not allow sufficient statistical power to detect seasonal variations in ASD occurrence among females. Hence, the analysis was restricted to males.

For peer review only - http://bmjopen.bmj.com/site/about/guidelines.xhtml

Methods

Settings

The 1994 National Health Insurance law mandates compulsory universal health coverage for all Israeli residents in a health maintenance organization (HMO) of their choice. Residents can choose one of four non-for profit HMOs operating across the country. Patient fees are equivalent across the four HMOs, and all provide equivalent medical services that are based on national health regulations. For this review, we examined data on ASD diagnosis from the computer database of the second-largest HMO, Maccabi Healthcare Services (MHS), which provides care to 2 million people (31% under 18 years of age) (Israeli Central Bureau of Statistics).

Study population

Using the computerized databases of MHS, we identified a total of 147,171 male children who were born between January 1999 and December 2008 (approximately 20% of all 736 thousand live male births in Israel during the same years). Children's parents were identified using data on household members in MHS. Children with missing paternal data (mostly due to previously belonging to a different health provider) were excluded from the study. Comparison of excluded (n=38,875) and eligible (n=110,093) children showed no statistically significant (P>0.05) differences in mean birth weight (3271 gram and 3315 gram, respectively), mean gestational week (39.0 and 39.1), or mother's age at birth (29.4 and 30.5) and a similar distribution of socioeconomic status (SES). . To avoid inaccurate paternal identification, an additional 1,545 children were excluded because more than one man was registered in the child's household. Hence, the total study population was 108,548 boys of 83,452 households.

For peer review only - http://bmjopen.bmj.com/site/about/guidelines.xhtml

Case identification and matching

A case was defined as a child with a physician–recorded diagnosis of autistic disorder, Asperger's syndrome, or PDD-NOS, based on the DSM-IV (American Psychiatric Association 1994). In MHS, children with suspected ASD are referred to child development centers for a multidisciplinary assessment for diagnosis validation. MHS' registry of ASD patients and validation processes were previously described[17].

Other study variables

Socioeconomic status (SES) was assessed according to the poverty index of the child's address enumeration area, as defined by 2008 national census[18] . The poverty index is based on several parameters including household income, educational qualifications, crowding, material conditions, and car ownership, census of population and housing. SES levels range between 1 (lowest) to 10 (highest). In this study SES below median (6) was considered as low SES. Month of conception was estimated based on the child's date of birth and the length of gestation as reported in the birth certificate. We categorized season of birth (and conception) as winter comprising December, January and February; spring, March, April and May; summer, June, July and August; and fall, September, October and November.

Statistical Analyses

First, bivariate analyses were conducted between month of birth and other covariates previously reported to be associated with increased risk of autism [19 20]. The covariates included gestational age in week as a continuous variable [21], birth weight, older maternal or paternal age (>35 years), and SES level above median (>6). Gestational age was available for 97264 individuals (90% of study population). Missing gestational age were imputed using multiple imputation (5 imputations) with birth weight as a covariate.

For peer review only - http://bmjopen.bmj.com/site/about/guidelines.xhtml

The Cochran-Armitage test was used to assess a significant trend in the association between month of birth and ASD.

Multivariable logistic regression analyses were performed to determine the odds ratio (OR) and 95% confidence interval CI for ASD by month of birth, using January as the reference month. For the purpose of comparison with previous studies, we fitted alternative logistic regression models, with month of conception (rather than birth) as the main independent variable. To evaluate the potential effect modification by socio-economic level, the study population was divided into two groups in further analyses: low-medium (1-6) and medium-high (7-10). Effect modification by month of birth and SES was investigated by including a cross-product interaction term between the exposure variable (birth in August) and the SES group.

A two-tailed P value of <0.05 was considered statistically significant. All data processing and statistical analyses were performed using IBM Corp. Released 2013. IBM SPSS Statistics for Windows, Version 22.0. Armonk, NY: IBM Corp.).

Results

The mean number of monthly births ranged from 8318 in February to 9654 in August. In bivariate analyses, number of births, maternal age, paternal age, birth weight, gestational length and SES were not associated with the month of birth (Table 1). Of the 108,548 boys born during the study period, 975 were diagnosed with ASD, representing a prevalence rate of 9.0 per 1000 live births. Mean age at ASD diagnosis was 3.0 years (SE=0.07years). The highest incidences of ASD were recorded among children born in May and August: 10.3 and 10.2 per 1000, respectively, while the lowest incidences were in January and February (7.6 and 8.1 per 1000, respectively) (Table 2). The results by month of conception are shown on Table 3.

For peer review only - http://bmjopen.bmj.com/site/about/guidelines.xhtml

In a multivariable model, birth months of August and May were associated with a non-significantly (P=0.16 and P=0.14, respectively) increased odds ratio (OR) (Table 4). When the data were stratified by household socioeconomic level, the birth month of August in low SES was associated with an odds ratio of 1.71 (95% confidence interval: 1.06 – 2.74) of ASD. No other significant results were observed for other birth months or in children of higher SES (Table 5 and appendics) . There was a borderline effect modification between birth in August and SES level (P=0.07).

Discussion

In this retrospective cohort of males born in Israel during a period spanning ten years, children of low SES born in the month of August were at a modestly increased risk of being diagnosed with ASD. This is the one of the largest studies outside the US to describe the relationship between ASD incidence and month of birth.

The literature with respect to season of birth and risk of ASD is inconsistent. Of the 15 studies summarized in table 6 (all conducted in the northern hemisphere), comprising a total number of 31,777 cases, five studies (total n=23,355) reported an increased risk of ASD of individuals born in August. March was significantly associated with increased risk in eight studies (total n=3008), and three studies (total n=5839) found no significant association between month of birth and ASD occurrence. No other specific birth month was repeatedly reported to be associated with ASD. Birth in August was previously reported to be associated with increased risk of ASD in an Israeli study that comprised 188 cases[11],

There are several mechanisms by which season of birth may affect the developing brain, either directly on the fetus and newborn or indirectly through the mother during pregnancy or the postpartum period. Seasonal environmental factors that could be

For peer review only - http://bmjopen.bmj.com/site/about/guidelines.xhtml

associated with ASD include viral or other infections[22, 23], nutritional factors[24], and vitamin deficiencies[25]. The outcome of exposure to prenatal viral infection depends on many factors: the variation in susceptibility of the maternal and fetal host, including the maternal immune status, the infecting virus, the strain of virus, the developmental stage of the fetus, the amount of virus reaching the fetus— particularly the CNS and immune system, the route of access, genetics, and probably other factors[26]. Maternal viral infection has been cited as the "principal non-genetic cause of autism" [27]. Maternal infections (i.e. rubella, cytomegalovirus, influenza), prolonged fever and maternal inflammation cause changes in a variety of inflammatory cytokines that have been associated with ASD [28, 29]. Depending on the location of immune activation, persistent viral infection could lead to chronically elevated cytokine levels that can be produced directly in the brain or gain access to the CNS by crossing an immature blood-brain barrier (BBB), altering CNS development[30].

A few studies found influenza infection during pregnancy to be a factor that increases risk for infantile autism[22 31] especially in the first trimester [29].

There is evidence that low socioeconomic populations tend to be more hospitalized for influenza infections among lower SES populations[32 33] and epidemiological studies found increased risk of ASD following maternal hospitalization due to infection[34]. We do not have data regarding influenza infections in our population. However, according to the Israeli center of disease control[35] , January and then February are the months with the highest percentage of influenza cases (nearly 85% of all cases). These are the critical months during which time CNS develops in children born in August. Thus, the seasonality of birth of ASD cases born to women with lower SES level could be related to their higher hospitalozations due to Influenza compared to women of high SES level.

For peer review only - http://bmjopen.bmj.com/site/about/guidelines.xhtml

A role of vitamin D deficiency in the development of autism has been proposed [25]. Thus, seasonal fluctuations in vitamin D should also be considered. In the northern hemisphere, vitamin D levels are the lowest in winter time [36-38] ; however, a study of healthy volunteers from Israel found no differences in vitamin D deficiency rates between summer and winter[39] .

Strengths of the current study include the large population, cohort design, the use of systematic data that were collected prospectively, validated source of information of ASD diagnosis, and objective assessment of socioeconomic status. Some limitations should be discussed. We had no information about the ASD disease severity; data were missing in some cases for gestational week at birth; only males were included, contrasting with other studies that included both sexes; the information was lacking about family history and lack of information in our population on influenza infections and vaccines. However, these limitations do not seem to be relevant to month of birth, and thus would not be expected to affect study findings. In addition, SES assessment was conducted using data on current residential area, which is not necessarily the residential area during the time of pregnancy, nor necessarily identical to individual SES. The children who were born at the end of the year were younger than those born earlier in the year, but this is negligible because they were all at the age of at least six years when the study was done and most autistic children are diagnosed by this age [40].

For peer review only

For peer review only - http://bmjopen.bmj.com/site/about/guidelines.xhtml

CONCLUSIONS

We found an association between birth in August and ASD among children of lower SES levels. Increased risk of ASD among children born in August (or March) has been reported in several large cohorts, suggesting a true seasonal pattern. A deeper understanding of these findings with regard to the various and complex etiologic pathways leading to development of ASD will be of value to better understanding this disorder.

CONTRIBUTIONS OF AUTHORS:

HS and GC contributed substantially to the conception of the work, analysis, interpretation of data for the work, drafting the work and final approval of the version to be published. IS contributed substantially to the acquisition, interpretation of data for the work, revising it critically for important intellectual content and final approval of the version to be published.

COMPETING INTERESTS: None Declared.

FUNDING: None.

DATA SHARING STATEMENT: No additional data are available

ETHICS APPROVAL: This study was approved by Assuta Healthcare Systems IRB.

ACKNOWLEDGMENTS

This study is part of the MD thesis by Hila Shalev

For peer review only - http://bmjopen.bmj.com/site/about/guidelines.xhtml

Refrences

1. American Psychiatric Association. *Diagnostic and statistical manual of mental disorders*. Arlington, VA: American Psychiatric Association; 2013.
2. Newschaffer CJ, Croen LA, Daniels J, Giarelli E, Grether JK, Levy SE, Mandell DS, Miller LA, Pinto-Martin J, Reaven J, Reynolds AM, Rice CE, Schendel D and Windham GC. The epidemiology of autism spectrum disorders. *Annual review of public health*. 2007;28:235-58.
3. Senecky Y, Chodick G, Diamond G, Lobel D, Drachman R and Inbar D. Time trends in reported autistic spectrum disorders in Israel, 1972-2004. *Isr Med Assoc J*. 2009;11:30-3.
4. Torrey EF, Miller J, Rawlings R and Yolken RH. Seasonal birth patterns of neurological disorders. *Neuroepidemiology*. 2000;19:177-85.
5. Mortensen PB, Pedersen CB, Westergaard T, Wohlfahrt J, Ewald H, Mors O, Andersen PK and Melbye M. Effects of family history and place and season of birth on the risk of schizophrenia. *N Engl J Med*. 1999;340:603-8.
6. Torrey EF, Miller J, Rawlings R and Yolken RH. Seasonality of births in schizophrenia and bipolar disorder: a review of the literature. *Schizophr Res*. 1997;28:1-38.
7. Mick E, Biederman J and Faraone SV. Is season of birth a risk factor for attention-deficit hyperactivity disorder? *J Am Acad Child Adolesc Psychiatry*. 1996;35:1470-6.
8. Liederman J and Flannery KA. Fall conception increases the risk of neurodevelopmental disorder in offspring. *J Clin Exp Neuropsychol*. 1994;16:754-68.
9. Livingston R, Adam BS and Bracha HS. Season of birth and neurodevelopmental disorders: summer birth is associated with dyslexia. *J Am Acad Child Adolesc Psychiatry*. 1993;32:612-6.
10. Castrogiovanni P, Iapichino S, Pacchierotti C and Pieraccini F. Season of birth in psychiatry. A review. *Neuropsychobiology*. 1998;37:175-81.
11. Barak Y, Ring A, Sulkes J, Gabbay U and Elizur A. Season of birth and autistic disorder in Israel. *The American journal of psychiatry*. 1995;152:798-800.
12. Kolevzon A, Weiser M, Gross R, Lubin G, Knobler HY, Schmeidler J, Silverman JM and Reichenberg A. Effects of season of birth on autism spectrum disorders: fact or fiction? *The American journal of psychiatry*. 2006;163:1288-90.
13. Biederman J. Attention-deficit/hyperactivity disorder: a selective overview. *Biol Psychiatry*. 2005;57:1215-20.
14. Biederman J and Faraone SV. Attention-deficit hyperactivity disorder. *Lancet*. 2005;366:237-48.
15. Rappley MD. Clinical practice. Attention deficit-hyperactivity disorder. *N Engl J Med*. 2005;352:165-73.
16. Alaerts K, Swinnen SP and Wenderoth N. Sex differences in autism: a resting-state fMRI investigation of functional brain connectivity in males and females. *Soc Cogn Affect Neurosci*. 2016.
17. Davidovitch M, Hemo B, Manning-Courtney P and Fombonne E. Prevalence and incidence of autism spectrum disorder in an Israeli population. *J Autism Dev Disord*. 2013;43:785-93.
18. Israel Central Bureau of Statistics. *Statistical Abstracts of Israel 2011: Census of Population and Housing Publication*. Jerusalem: Israel Bureau of Statistics; 2011.

For peer review only - http://bmjopen.bmj.com/site/about/guidelines.xhtml

19. Roberts EM, English PB, Grether JK, Windham GC, Somberg L and Wolff C. Maternal residence near agricultural pesticide applications and autism spectrum disorders among children in the California Central Valley. *Environ Health Perspect*. 2007;115:1482-9.
20. Croen LA, Grether JK and Selvin S. Descriptive epidemiology of autism in a California population: who is at risk? *Journal of autism and developmental disorders*. 2002;32:217-24.
21. Leavey A, Zwaigenbaum L, Heavner K and Burstyn I. Gestational age at birth and risk of autism spectrum disorders in Alberta, Canada. *The Journal of pediatrics*. 2013;162:361-8.
22. Deykin EY and MacMahon B. Viral exposure and autism. *Am J Epidemiol*. 1979;109:628-38.
23. Lee BK, Magnusson C, Gardner RM, Blomström Å, Newschaffer CJ, Burstyn I, Karlsson H and Dalman C. Maternal hospitalization with infection during pregnancy and risk of autism spectrum disorders. *Brain Behav Immun*. 2015;44:100-5.
24. Lyall K, Schmidt RJ and Hertz-Picciotto I. Maternal lifestyle and environmental risk factors for autism spectrum disorders. *Int J Epidemiol*. 2014;43:443-64.
25. Grant WB and Soles CM. Epidemiologic evidence supporting the role of maternal vitamin D deficiency as a risk factor for the development of infantile autism. *Dermatoendocrinol*. 2009;1:223-8.
26. Blattner RJ. The role of viruses in congenital defects. *Am J Dis Child*. 1974;128:781-6.
27. Ciaranello AL and Ciaranello RD. The neurobiology of infantile autism. *Annu Rev Neurosci*. 1995;18:101-28.
28. Ornoy A, Weinstein-Fudim L and Ergaz Z. Prenatal factors associated with autism spectrum disorder (ASD). *Reprod Toxicol*. 2015;56:155-69.
29. Atladóttir H, Henriksen TB, Schendel DE and Parner ET. Autism after infection, febrile episodes, and antibiotic use during pregnancy: an exploratory study. *Pediatrics*. 2012;130:e1447-54.
30. Muñoz-Fernández MA and Fresno M. The role of tumour necrosis factor, interleukin 6, interferon-gamma and inducible nitric oxide synthase in the development and pathology of the nervous system. *Prog Neurobiol*. 1998;56:307-40.
31. Zhang X, Lv CC, Tian J, Miao RJ, Xi W, Hertz-Picciotto I and Qi L. Prenatal and perinatal risk factors for autism in China. *Journal of autism and developmental disorders*. 2010;40:1311-21.
32. Tam K, Yousey-Hindes K and Hadler JL. Influenza-related hospitalization of adults associated with low census tract socioeconomic status and female sex in New Haven County, Connecticut, 2007-2011. *Influenza Other Respir Viruses*. 2014;8:274-81.
33. Sloan C, Chandrasekhar R, Mitchel E, Schaffner W and Lindegren ML. Socioeconomic Disparities and Influenza Hospitalizations, Tennessee, USA. *Emerg Infect Dis*. 2015;21:1602-10.
34. Atladóttir HO, Thorsen P, Østergaard L, Schendel DE, Lemcke S, Abdallah M and Parner ET. Maternal infection requiring hospitalization during pregnancy and autism spectrum disorders. *Journal of autism and developmental disorders*. 2010;40:1423-30.
35. Control. ICfD. Israel Center for Disease Control. Influenza Surveillance in the Winter Season in Israel. ·
36. Kasahara AK, Singh RJ and Noymer A. Vitamin D (25OHD) Serum Seasonality in the United States. *PLoS One*. 2013;8:e65785.
37. Kashi Z, Saeedian F, Akha O, Gorgi M, Emadi S and Zakeri H. Vitamin D deficiency prevalence in summer compared to winter in a city with high humidity and a sultry climate. *Endokrynol Pol*. 2011;62:249-51.

For peer review only

38. Costanzo PR, Constanzo PR, Elías NO, Kleiman Rubinsztein J, García Basavilbaso NX, Piacentini R and Salerni HH. [Ultraviolet radiation impact on seasonal variations of serum 25-hydroxy-vitamin D in healthy young adults in Buenos Aires]. *Medicina (B Aires)*. 2011;71:336-42.
39. Oren Y, Shapira Y, Agmon-Levin N, Kivity S, Zafrir Y, Altman A, Lerner A and Shoenfeld Y. Vitamin D insufficiency in a sunny environment: a demographic and seasonal analysis. *Isr Med Assoc J*. 2010;12:751-6.
40. Hertz-Picciotto I and Delwiche L. The rise in autism and the role of age at diagnosis. *Epidemiology*. 2009;20:84-90.
41. Zerbo O, Iosif AM, Delwiche L, Walker C and Hertz-Picciotto I. Month of conception and risk of autism. *Epidemiology*. 2011;22:469-75.
42. Mazumdar S, Liu KY, Susser E and Bearman P. The disappearing seasonality of autism conceptions in California. *PLoS One*. 2012;7:e41265.
43. Bolton P, Pickles A, Harrington R, Macdonald H and Rutter M. Season of birth: issues, approaches and findings for autism. *Journal of child psychology and psychiatry, and allied disciplines*. 1992;33:509-30.
44. Lee LC, Newschaffer CJ, Lessler JT, Lee BK, Shah R and Zimmerman AW. Variation in season of birth in singleton and multiple births concordant for autism spectrum disorders. *Paediatr Perinat Epidemiol*. 2008;22:172-9.
45. Bartlik BD. Monthly variation in births of autistic children in North Carolina. *Journal of the American Medical Women's Association (1972)*. 1981;36:363-8.
46. Landau EC, Cicchetti DV, Klin A and Volkmar FR. Season of birth in autism: a fiction revisited. *J Autism Dev Disord*. 1999;29:385-93.
47. Mouridsen SE, Nielsen S, Rich B and Isager T. Season of birth in infantile autism and other types of childhood psychoses. *Child psychiatry and human development*. 1994;25:31-43.
48. Konstantareas MM, Hauser P, Lennox C and Homatidis S. Season of birth in infantile autism. *Child psychiatry and human development*. 1986;17:53-65.
49. Stevens MC, Fein DH and Waterhouse LH. Season of birth effects in autism. *J Clin Exp Neuropsychol*. 2000;22:399-407.
50. Gillberg C. Do children with autism have March birthdays? *Acta Psychiatr Scand*. 1990;82:152-6.
51. Tanoue Y, Oda S, Asano F and Kawashima K. Epidemiology of infantile autism in southern Ibaraki, Japan: differences in prevalence in birth cohorts. *Journal of autism and developmental disorders*. 1988;18:155-66.
52. Hebert KJ, Miller LL and Joinson CJ. Association of autistic spectrum disorder with season of birth and conception in a UK cohort. *Autism research : official journal of the International Society for Autism Research*. 2010;3:185-90.

For peer review only

Table 1 Study population characteristics by month of birth

Month	Births	SES* 1 to 10				Gestational Age				Birth weight, g				Paternal age				Maternal age			
		75%	25%	Med	M (SD)	75%	25 %	Med	M (SD)	75%	25%	Med	M (SD)	75%	25%	Med	M (SD)	75%	25%	Med	M (SD)
Jan	*9367*	8	4	6	6.1 (2.3)	40	38	39	39.1 (1.9)	3630	3000	3320	3296 (525)	36	29	33	32.9 (5.7)	33	*27*	*30*	30.1 (4.9)
Feb	*8318*	8	5	6	6.2 (2.3)	40	38	39	39.0 (2.0)	3640	3000	3320	3296 (528)	36	29	33	32.9 (5.7)	34	*27*	*30*	30.2 (4.9)
Mar	*9038*	8	4	6	6.1 (2.3)	40	38	39	39.0 (1.9)	3650	3020	3340	3305 (519)	36	29	33	32.9 (5.6)	34	*27*	*30*	30.2 (4.9)
Apr	*8465*	8	5	6	6.2 (2.3)	40	38	39	39.1 (1.9)	3640	3030	3340	3310 (519)	37	29	33	33.0 (5.6)	34	*27*	*30*	30.3 (4.9)
May	*8711*	8	5	6	6.3 (2.3)	40	38	39	39.1 (1.9)	3640	3010	3330	3300 (532)	37	29	33	33.0 (5.6)	34	*27*	*30*	30.3 (4.9)
Jun	*8523*	8	5	6	6.2 (2.3)	40	38	39	39.0 (2.0)	3650	3010	3350	3306 (538)	37	29	33	33.1 (5.6)	34	*27*	*30*	30.3 (5.0)
Jul	*9330*	8	4	6	6.1 (2.3)	40	38	39	39.0 (2.0)	3650	3010	3330	3304 (528)	37	29	33	33.0 (5.7)	34	*27*	*30*	30.2 (4.9)
Aug	*9654*	8	4	6	6.1 (2.3)	40	38	39	39.0 (1.9)	3660	3020	3340	3312 (532)	37	29	33	33.1 (5.7)	34	*27*	*30*	30.3 (4.9)
Sep	*9270*	8	4	6	6.1 (2.3)	40	38	39	39.0 (1.9)	3640	3000	3320	3291 (537)	37	29	33	33.1 (5.7)	34	*27*	*30*	30.3 (4.9)
Oct	*9521*	8	4	6	6.1 (2.3)	40	38	39	39.1 (1.9)	3640	3020	3330	3302 (524)	37	29	33	33.0 (5.7)	34	*27*	*30*	30.3 (5.0)
Nov	*9069*	8	4	6	6.1 (2.3)	40	38	39	39.0 (1.9)	3640	3000	3330	3302 (532)	37	29	33	33.1 (5.7)	34	*27*	*30*	30.3 (5.0)
Dec	*9282*	8	4	6	6.1 (2.3)	40	38	39	39.0 (2.0)	3630	3000	3320	3288 (540)	37	29	33	33.0 (5.7)	34	*27*	*30*	30.3 (5.0)
**Total**	***108548***	**8**	**4.33**	**6**	**6.1 (2.3)**	**39.0 (2.0)**	**38**	**39**	**39.0 (2.0)**	**3301 (529 .5)**	**3010**	**3330.8**	**3301 (529.5)**	**33.0 (5.7)**	**29**	**33**	**33.0 (5.7)**	**33.9**	***27***	***30***	**30.3 (5)**

- See text for details on definitions of SES

For peer review only

Table 2: Incidence rate and 95% confidence intervals (95%CI) per 1000 male live births of Autistic spectrum disorder (ASD), by month of birth

Month of birth	ASD cases	Rate (per 1000)	95% CI*	
Jan	76	8.1	6.44	10.09
Feb	63	7.6	5.87	9.62
Mar	74	8.2	6.48	10.21
Apr	74	8.7	6.92	10.90
May	90	10.3	8.36	12.62
Jun	78	9.2	7.29	11.35
Jul	89	9.5	7.71	11.67
Aug	98	10.2	8.29	12.30
Sep	80	8.6	6.89	10.67
Oct	85	8.9	7.18	10.97
Nov	84	9.3	7.44	11.40
Dec	84	9.0	7.27	11.14
Total	975	8.96	7.17	11.07

* see text for details

Table 3: Incidence rate and 95% confidence intervals (95%CI) per 1000 male live births of Autistic spectrum disorder (ASD), by month of conception

Month of conception	ASD cases	Rate (per 1000)	95% CI*	
Jan	87	9.2	7.4	11.3
Feb	74	8.7	6.9	11
Mar	77	8.3	6.6	10.4
Apr	72	8.0	6.3	10.1
May	77	8.4	6.7	10.5
Jun	68	7.8	6.1	9.9
Jul	72	8.2	6.4	10.3
Aug	89	10.2	8.2	12.5
Sep	91	10.9	8.8	13.4
Oct	79	8.4	6.6	10.4
Nov	99	10.8	8.8	13.1
Dec	87	9.1	7.3	11.2
Total	972	9.0	8.4	9.9

* see text for details

For peer review only

For peer review only - http://bmjopen.bmj.com/site/about/guidelines.xhtml

Table 4: Crude and adjusted odd ratio (OR) and 95% confidence interval of autistic spectrum disorder (ASD) for month of birth: multivariable logistic regression

Month of birth	OR crude	95%CI		OR adjusted*	95%CI	
Jan	1 (ref.)			1 (ref.		
Feb	.93	.67	1.31	.92	.66	1.29
Mar	1.01	.73	1.39	1.00	.73	1.38
Apr	1.08	.78	1.49	1.08	.78	1.48
May	1.28	.94	1.73	1.26	.92	1.71
Jun	1.13	.82	1.55	1.11	.81	1.52
Jul	1.16	.86	1.58	1.15	.84	1.56
Aug	1.25	.93	1.69	1.24	.92	1.68
Sep	1.07	.78	1.46	1.05	.77	1.44
Oct	1.10	.81	1.50	1.09	.80	1.49
Nov	1.13	.83	1.55	1.12	.82	1.53
Dec	1.11	.81	1.51	1.09	.80	1.50

*Adjusted for child's age, father's age, mother's age, district, birth weight, gestational age with imputation (see text for details).

For peer review only

Table 5: Crude and adjusted odd ratio (OR) and 95% confidence interval from multivariable logistic regression of autistic spectrum disorder (ASD) for month of birth, by socioeconomic level

	SES 1-6						SES 7-10					
**Month of birth**	**OR crude**	**95%CI**		**OR adjusted***	**95%CI**		**OR crude**	**95%CI**		**OR adjusted***	**95%CI**	
Jan	1 (ref.)			1 (ref.)			1 (ref.)			1 (ref.)		
Feb	1.01	0.58	1.75	1.01	0.58	1.75	0.89	0.58	1.35	0.88	0.57	1.34
Mar	1.34	0.80	2.22	1.34	0.81	2.22	0.82	0.54	1.25	0.81	0.53	1.24
Apr	1.30	0.77	2.19	1.32	0.78	2.22	0.93	0.62	1.40	0.93	0.62	1.40
May	1.31	0.78	2.20	1.30	0.78	2.19	1.19	0.81	1.74	1.17	0.80	1.72
Jun	1.26	0.75	2.12	1.25	0.74	2.11	1.01	0.67	1.51	0.99	0.66	1.49
Jul	1.37	0.83	2.25	1.36	0.82	2.25	1.01	0.68	1.49	0.99	0.67	1.48
Aug	1.70	1.06	2.73	1.71	1.07	2.76	1.00	0.67	1.48	0.99	0.66	1.46
Sep	1.32	0.80	2.19	1.32	0.80	2.18	0.92	0.61	1.38	0.91	0.60	1.36
Oct	1.24	0.75	2.05	1.24	0.75	2.07	1.03	0.69	1.52	1.01	0.68	1.50
Nov	1.45	0.88	2.37	1.46	0.89	2.40	0.96	0.64	1.44	0.93	0.62	1.40
Dec	1.49	0.91	2.43	1.51	0.92	2.46	0.89	0.59	1.34	0.88	0.58	1.32

*Adjusted for child's age, father's age, mother's age, district, birth weight, age with imputation (see text for details).

For peer review only

For peer review only - http://bmjopen.bmj.com/site/about/guidelines.xhtml

Table 6: Summary of Findings in Previous Studies Investigating the Relationship Between Seasonal Variations of Birth and Autism

Ref.	Study period	Place	ASD cases	Months of increased risk of ASD
41	1990 – 2002	California	19,238	Aug, Sep, Oct, Nov, Dec
42	1992 – 2000	California	2,940	Aug*
42	1997 – 1999	California	3,897	No Association
43	1947 – 1980	UK	1631	No Association
44	1983 – 2002	Maryland	1068	Spring
45	1981***	N Carolina	810	Mar, Aug
46	1947-1992	Child dev. clinic	620	No Association**
47	1945 – 1980	Denmark	328	March
12	1980 – 1985	Israel	311	No Association
11	1964 – 1986	Israel	188	March and August
48	1980s	Ottario, Canada	179	Summer and Spring
49	1977 – 1985	US	175	March
50	1962 – 1984	Sweden	174	March
51	1972 – 1978	Japan	132	Spring-Summer (Apr, May, Jun)
52	1990–1992	UK	86	Spring

- Conception in November (For years 1994 – 1996) ** (P<0.1 for middle of August for verbal ASD) *** year of publication

For peer review only

For peer review only

For peer review only - http://bmjopen.bmj.com/site/about/guidelines.xhtml

Appendix

Table a: Crude and adjusted odd ratio (OR) and 95% confidence interval from multivariable logistic regression of autistic spectrum disorder (ASD) for month of conception, by socioeconomic status (SES)

	SES 1-6						SES 7-10					
**Month of conception**	**OR crude**	**95%CI**		**OR adjusted***	**95%CI**		**OR crude**	**95%CI**		**OR adjusted***	**95%CI**	
Jan	1 (ref.)			1 (ref.			1 (ref.)			1 (ref.		
Feb	.89	.52	1.50	.89	.52	1.51	1.09	.72	1.65	1.08	.72	1.63
Mar	1.13	.70	1.85	1.14	.70	1.86	.84	.55	1.30	.84	.54	1.29
Apr	.90	.53	1.53	.89	.53	1.52	1.06	.71	1.60	1.09	.72	1.64
May	1.05	.63	1.75	1.03	.62	1.71	.94	.62	1.44	.95	.62	1.45
Jun	1.19	.72	1.96	1.17	.71	1.93	.74	.46	1.16	.75	.47	1.18
Jul	.79	.45	1.38	.79	.45	1.38	.96	.63	1.45	.97	.64	1.47
Aug	1.11	.67	1.83	1.09	.66	1.81	1.13	.76	1.68	1.13	.76	1.68
Sep	1.29	.79	2.10	1.28	.78	2.08	1.09	.73	1.64	1.10	.73	1.65
Oct	.95	.57	1.58	.96	.58	1.59	.88	.58	1.34	.89	.58	1.35
Nov	1.14	.70	1.86	1.14	.70	1.86	1.16	.78	1.72	1.16	.78	1.72
Dec	1.18	.74	1.91	1.18	.74	1.91	.90	.59	1.36	.89	.59	1.36

*Adjusted for child's age, father's age, mother's age, district, birth weight, age with imputation (see text for details).

For peer review only

Table b: Crude and adjusted odd ratio (OR) and 95% confidence interval from multivariable logistic regression of autistic spectrum disorder (ASD) for season of birth, by socioeconomic status (SES)

	SES median-high						SES median-low					
**Season of birth**	**OR crude**	**95%CI**		**OR adjusted***	**95%CI**		**OR crude**	**95%CI**		**OR adjusted***	**95%CI**	
Winter	1 (ref.)			1 (ref.			1 (ref.)			1 (ref.		
Spring	1.12	0.84	1.50	1.12	0.84	1.50	1.06	0.83	1.34	1.06	0.83	1.34
Summer	1.24	0.94	1.63	1.23	0.93	1.63	1.08	0.85	1.37	1.08	0.85	1.37
Fall	1.14	0.86	1.51	1.14	0.86	1.51	1.05	0.82	1.33	1.04	0.82	1.32

*Adjusted for child's age, father's age, mother's age, district, birth weight, gestational age with imputation

For peer review only

Table c: Crude and adjusted odd ratio (OR) and 95% confidence interval from multivariable logistic regression of autistic spectrum disorder (ASD) for season of conception, by socioeconomic status (SES)

	SES median-high						SES median-low					
**Season of conception**	**OR crude**	**95%CI**		**OR adjusted***	**95%CI**		**OR crude**	**95%CI**		**OR adjusted***	**95%CI**	
**Winter**	1 (ref.)			1 (ref.			1 (ref.)			1 (ref.		
**Spring**	1.00	0.75	1.34	0.99	0.74	1.33	0.96	0.75	1.22	0.96	0.75	1.23
**Summer**	1.00	0.74	1.35	0.99	0.73	1.33	0.96	0.75	1.22	0.97	0.76	1.24
**Fall**	1.09	0.82	1.45	1.08	0.82	1.44	1.05	0.83	1.33	1.06	0.83	1.34

*Adjusted for child's age, father's age, mother's age, district, birth weight, gestational age with imputation

For peer review only

Table d: Crude and adjusted odd ratio (OR) and 95% confidence interval from multivariable logistic regression of autistic spectrum disorder (ASD) for first month of second trimester, by socioeconomic status (SES)

Month of second trimester	OR crude	95%CI		OR adjusted*	95%CI		OR crude	95%CI		OR adjusted*	95%CI	
**Jan**	1 (ref.)			1 (ref.			1 (ref.)			1 (ref.		
**Feb**	.83	.51	1.35	.83	.51	1.36	1.03	.67	1.57	1.03	.67	1.57
**Mar**	1.08	.69	1.69	1.08	.69	1.69	.87	.57	1.35	.86	.56	1.33
**Apr**	.89	.55	1.43	.89	.55	1.43	1.12	.74	1.69	1.11	.73	1.67
**May**	.68	.41	1.12	.68	.41	1.13	.99	.65	1.51	.98	.64	1.49
**Jun**	.87	.53	1.41	.87	.54	1.41	1.09	.72	1.64	1.07	.71	1.63
**Jul**	1.00	.62	1.59	.98	.61	1.56	1.02	.67	1.56	1.04	.68	1.59
**Aug**	.74	.44	1.23	.72	.43	1.20	.97	.63	1.49	.97	.63	1.49
**Sep**	.94	.58	1.54	.93	.57	1.51	.86	.55	1.35	.87	.55	1.36
**Oct**	.84	.51	1.37	.83	.51	1.37	1.10	.73	1.66	1.11	.73	1.67
**Nov**	.84	.51	1.40	.83	.50	1.38	1.25	.84	1.87	1.24	.83	1.86
**Dec**	.83	.51	1.36	.83	.51	1.36	1.10	.73	1.65	1.09	.72	1.64

*Adjusted for child's age, father's age, mother's age, district, birth weight, age with imputation (see text for details).

For peer review only

Table d: Crude and adjusted odd ratio (OR) and 95% confidence interval from multivariable logistic regression of autistic spectrum disorder (ASD) for first month of third trimester, by socioeconomic status (SES)

Month of third trimester	OR crude	95%CI		OR adjusted*	95%CI		OR crude	95%CI		OR adjusted*	95%CI	
Jan	1 (ref.)			1 (ref.			1 (ref.)			1 (ref.		
Feb	1.04	0.61	1.77	1.04	0.61	1.78	1.22	0.81	1.82	1.21	0.81	1.81
Mar	0.97	0.57	1.63	0.97	0.58	1.64	0.96	0.63	1.46	0.95	0.63	1.45
Apr	1.19	0.73	1.95	1.20	0.74	1.97	1.13	0.75	1.68	1.12	0.75	1.68
May	0.86	0.51	1.46	0.87	0.52	1.48	0.86	0.56	1.32	0.86	0.56	1.31
Jun	1.26	0.77	2.04	1.27	0.78	2.06	0.84	0.55	1.30	0.83	0.54	1.27
Jul	0.99	0.60	1.65	1.01	0.61	1.67	1.07	0.71	1.61	1.05	0.70	1.58
Aug	0.78	0.45	1.33	0.79	0.46	1.36	0.99	0.66	1.51	0.98	0.64	1.48
Sep	1.12	0.68	1.86	1.12	0.68	1.86	1.12	0.75	1.69	1.12	0.74	1.69
Oct	0.84	0.49	1.44	0.83	0.48	1.42	0.83	0.53	1.29	0.83	0.54	1.30
Nov	0.92	0.53	1.57	0.91	0.53	1.56	1.00	0.65	1.53	1.00	0.65	1.54
Dec	1.07	0.64	1.80	1.08	0.64	1.81	0.81	0.52	1.26	0.81	0.52	1.26

*Adjusted for child's age, father's age, mother's age, district, birth weight, age with imputation (see text for details).

For peer review only

STROBE 2007 (v4) checklist of items to be included in reports of observational studies in epidemiology*
Checklist for cohort, case-control, and cross-sectional studies (combined)

Section/Topic	Item #	Recommendation	Reported on page #
**Title and abstract**	1	(*a*) Indicate the study's design with a commonly used term in the title or the abstract	1-2
		(*b*) Provide in the abstract an informative and balanced summary of what was done and what was found	1-2
**Introduction**			
Background/rationale	2	Explain the scientific background and rationale for the investigation being reported	4
Objectives	3	State specific objectives, including any pre-specified hypotheses	4
**Methods**			
Study design	4	Present key elements of study design early in the paper	2
Setting	5	Describe the setting, locations, and relevant dates, including periods of recruitment, exposure, follow-up, and data collection	2
Participants	6	(*a*) *Cohort study*—Give the eligibility criteria, and the sources and methods of selection of participants. Describe methods of follow-up<br>*Case-control study*—Give the eligibility criteria, and the sources and methods of case ascertainment and control selection. Give the rationale for the choice of cases and controls<br>*Cross-sectional study*—Give the eligibility criteria, and the sources and methods of selection of participants	5
		(*b*) *Cohort study*—For matched studies, give matching criteria and number of exposed and unexposed<br>*Case-control study*—For matched studies, give matching criteria and the number of controls per case	-
Variables	7	Clearly define all outcomes, exposures, predictors, potential confounders, and effect modifiers. Give diagnostic criteria, if applicable	8-7
Data sources/ measurement	8*	For each variable of interest, give sources of data and details of methods of assessment (measurement). Describe comparability of assessment methods if there is more than one group	5
Bias	9	Describe any efforts to address potential sources of bias	-
Study size	10	Explain how the study size was arrived at	5
Quantitative variables	11	Explain how quantitative variables were handled in the analyses. If applicable, describe which groupings were chosen and why	6
Statistical methods	12	(*a*) Describe all statistical methods, including those used to control for confounding	6
		(*b*) Describe any methods used to examine subgroups and interactions	6
		(*c*) Explain how missing data were addressed	5
		(*d*) *Cohort study*—If applicable, explain how loss to follow-up was addressed<br>*Case-control study*—If applicable, explain how matching of cases and controls was addressed	5

For peer review only

		Cross-sectional study—If applicable, describe analytical methods taking account of sampling strategy	
		(*e*) Describe any sensitivity analyses	-
**Results**			
Participants	13*	(a) Report numbers of individuals at each stage of study—eg numbers potentially eligible, examined for eligibility, confirmed eligible, included in the study, completing follow-up, and analysed	5
		(b) Give reasons for non-participation at each stage	5
		(c) Consider use of a flow diagram	-
Descriptive data	14*	(a) Give characteristics of study participants (eg demographic, clinical, social) and information on exposures and potential confounders	5
		(b) Indicate number of participants with missing data for each variable of interest	5
		(c) *Cohort study*—Summarise follow-up time (eg, average and total amount)	-
Outcome data	15*	*Cohort study*—Report numbers of outcome events or summary measures over time	-
		Case-control study—Report numbers in each exposure category, or summary measures of exposure	5
		Cross-sectional study—Report numbers of outcome events or summary measures	-
Main results	16	(*a*) Give unadjusted estimates and, if applicable, confounder-adjusted estimates and their precision (eg, 95% confidence interval). Make clear which confounders were adjusted for and why they were included	-
		(*b*) Report category boundaries when continuous variables were categorized	-
		(*c*) If relevant, consider translating estimates of relative risk into absolute risk for a meaningful time period	18 ,17
Other analyses	17	Report other analyses done—eg analyses of subgroups and interactions, and sensitivity analyses	6
**Discussion**			
Key results	18	Summarise key results with reference to study objectives	7-8
Limitations	19	Discuss limitations of the study, taking into account sources of potential bias or imprecision. Discuss both direction and magnitude of any potential bias	3+10
Interpretation	20	Give a cautious overall interpretation of results considering objectives, limitations, multiplicity of analyses, results from similar studies, and other relevant evidence	7-8
Generalisability	21	Discuss the generalisability (external validity) of the study results	7-8
**Other information**			
Funding	22	Give the source of funding and the role of the funders for the present study and, if applicable, for the original study on which the present article is based	Not Relevant

*Give information separately for cases and controls in case-control studies and, if applicable, for exposed and unexposed groups in cohort and cross-sectional studies.

For peer review only

Note: An Explanation and Elaboration article discusses each checklist item and gives methodological background and published examples of transparent reporting. The STROBE checklist is best used in conjunction with this article (freely available on the Web sites of PLoS Medicine at http://www.plosmedicine.org/, Annals of Internal Medicine at http://www.annals.org/, and Epidemiology at http://www.epidem.com/). Information on the STROBE Initiative is available at www.strobe-statement.org.

For peer review only

BMJ Open

Month of birth and risk of autism spectrum disorder, a retrospective cohort of male children born in Israel

Journal:	*BMJ Open*
Manuscript ID	bmjopen-2016-014606.R2
Article Type:	Research
Date Submitted by the Author:	17-Feb-2017
Complete List of Authors:	Shalev, Hila; Technion Israel Institute of Technology Ruth and Bruce Rappaport Faculty of Medicine, Medicine<br>Chodick, Gabriel ; Sackler School of Medicine Tel Aviv University, Ramat Aviv, Israel, Epidemiology and Database Research<br>Solt, Ido; Rambam Health Care Campus; Technion Israel Institute of Technology Ruth and Bruce Rappaport Faculty of Medicine, Medicine
**Primary Subject Heading**:	Epidemiology
Secondary Subject Heading:	Paediatrics, Public health
Keywords:	EPIDEMIOLOGY, Paediatric neurology < NEUROLOGY, Maternal medicine < OBSTETRICS, Developmental neurology & neurodisability < PAEDIATRICS

SCHOLARONE™
Manuscripts

For peer review only

Month of birth and risk of autism spectrum disorder, a retrospective cohort of male children born in Israel

Hila Shalev BMD[1], Ido Solt MD[4,5], Gabriel Chodick PhD[2,3]

[1] Faculty of Medicine, Technion, Haifa, Israel

[2] Maccabi Healthcare Services, Tel Aviv, Israel,

[3] Sackler Faculty of Medicine, Tel Aviv University, Tel Aviv, Israel

[4] Rambam Health Care campus

[5] The Rappaport Faculty of Medicine, Technion

Corresponding author:

Gabriel Chodick PhD

Epidemiology & Database Research Medical Division, Maccabi Healthcare Services, Tel-Aviv, Israel

School of Public Health, Faculty of Medicine, Tel-Aviv University Tel-Aviv Israel

Email. hodik_g@mac.org.il

Tel: 972.3795.2430

For peer review only - http://bmjopen.bmj.com/site/about/guidelines.xhtml

Abstract

Background: Increased incidence and prevalence of autism spectrum disorder (ASD) over the last two decades have prompted considerable efforts to investigate its etiological factors. We examined an association between month of birth and ASD incidence.

Methods: In a retrospective cohort of male children born from January 1999 to December 2008 in a large health organization in Israel (Maccabi Healthcare Services), ASD was followed from birth through December 2015.

Results: Of 108,548 boys, 975 cases of ASD were identified. The highest rates (10.3 and 10.2 per 1000 male live births) were recorded for children born in May and August, and the lowest rates for February (7.6 per 1000 male live births). Among lower socioeconomic status households, boys born in August were more likely (OR=1.71; 95% confidence interval: 1.06 – 2.74) of being diagnosed with ASD than children born in January. Significantly higher rates were not observed for other months.

Conclusions: In line with several previous studies, we found modestly higher likelihood of autism occurrence among male children of lower socioeconomic levels born in August.

For peer review only - http://bmjopen.bmj.com/site/about/guidelines.xhtml

For peer review only

Strengths of the current study include:

- Large population
- Cohort design
- The use of systematic data that were collected prospectively
- Validated source of information of ASD diagnosis
- Objective assessment of socioeconomic status.

Limitations of the study:

- No information was available regarding ASD disease severity
- Data were missing in some cases for gestational week at birth.
- Only males were included, contrasting with other studies that included both sexes.
- Lack of information in our population on influenza infections and vaccines.
- SES was assessed using data on current residential area, which is not necessarily the residential area during the time of pregnancy.

However, these limitations do not seem to be relevant to month of birth, and thus would not be expected to affect study findings.

For peer review only

Introduction

Autism spectrum disorder (ASD) is a biologically-based neurodevelopmental condition characterized by a) deficits in social communication and social interaction, and b) restricted repetitive patterns of behavior, interests, and activities [1]. The prevalence of ASD has been rising [2] globally, and in Israel in particular [3].

Analyses of season of birth or conception can provide clues about causes of diseases with unknown etiology as in ASD. Patterns of seasonal variation in births have been found in several disorders including schizophrenia, mood disorders, epilepsy, language disorders, attention deficit hyperactivity disorder (ADHD) and neurodevelopmental disabilities[4-9]. An association between season of birth or conception suggests periodicity of an environmental etiologic factor active during the prenatal, perinatal or early postnatal phase of development [10]. Seasonal effects observed in these disorders (especially in schizophrenia) prompted several researchers to consider whether the same would apply to autism spectrum disorders (ASD). The findings of such investigations are inconsistent, most researchers found an association but not to particular months, and others claimed the did not find an association at all. Inconsistencies in the literature could be due, in part, to a wide range of study populations, geographical areas, case definitions, comparison groups and analytical approaches.

In one of the first studies on season of birth and ASD to be carried out in a region areas other than the US and western Europe, a study in Israel[11] found an increase in March and August births among 188 ASD cases when compared to the general population. The replication of seasonal patterns in a region with a climate and environment different from those of the countries previously studied supports the validity of the findings. However, a later examination of all ASD cases (N=211) among Jews in Israel over five

For peer review only - http://bmjopen.bmj.com/site/about/guidelines.xhtml

years revealed no seasonal patterns in birth period. Such inconsistency can be explained by methodological differences, selection of comparison group, lack of statistical power, and use of different diagnostic criteria.

The objective of the present study was to examine a relationship between autism and seasonality in a large population in Israel, using the computerized databases of a single health provider and its standardized coding system. Male to female ratio among ASD patients is reported between 3 to 1 and 4 to 1 [12, 13 14], reflecting a disorder of potential differences in mechanisms and etiology between the two sexes[15-18]. We excluded the females from this investigation as we were interested in controlling the impact of sex by restricting the sample to boys.

Methods

Settings

The 1994 National Health Insurance law mandates compulsory universal health coverage for all Israeli residents in a health maintenance organization (HMO) of their choice. Residents can choose one of four non-for profit HMOs operating across the country. Patient fees are equivalent across the four HMOs, and all provide equivalent medical services that are based on national health regulations. For this review, we examined data on ASD diagnosis from the computer database of the second-largest HMO, Maccabi Healthcare Services (MHS), which provides care to 2 million people (31% under 18 years of age) (Israeli Central Bureau of Statistics).

For peer review only - http://bmjopen.bmj.com/site/about/guidelines.xhtml

Study population

Using the computerized databases of MHS, we identified a total of 147,171 male children who were born between January 1999 and December 2008 (approximately 20% of all 736 thousand live male births in Israel during the same years). Children's parents were identified using data on household members in MHS. Children with missing paternal data (mostly due to previously belonging to a different health provider) were excluded from the study. Comparison of excluded (n=38,875) and eligible (n=110,093) children showed no statistically significant (P>0.05) differences in mean birth weight (3271 gram and 3315 gram, respectively), mean gestational week (39.0 and 39.1), or mean mother's age at birth (29.4 and 30.5) and a similar distribution of socioeconomic status (SES). To avoid inaccurate paternal identification, an additional 1,545 children were excluded because more than one man was registered in the child's household. Hence, the total study population was 108,548 boys of 83,452 households.

Case identification and matching

A case was defined as a child with a physician–recorded diagnosis of autistic disorder, Asperger's syndrome, or PDD-NOS, based on the DSM-IV (American Psychiatric Association 1994). In MHS, children with suspected ASD are referred to child development centers for a multidisciplinary assessment for diagnosis validation. The MHS registry of ASD patients and validation processes were previously described[19].

Other study variables

Socioeconomic status (SES) was assessed according to the poverty index of the child's address enumeration area, as defined by 2008 national census[20] . The poverty index is based on several parameters including household income, educational qualifications, crowding, material conditions, and car ownership, census of population

For peer review only - http://bmjopen.bmj.com/site/about/guidelines.xhtml

and housing. SES levels range between 1 (lowest) to 10 (highest). In this study SES below median (6) was considered as low SES. Calendar year and month of conception was estimated based on the child's date of birth and the length of gestation as reported in the birth certificate. We categorized season of birth (and conception) as winter comprising December, January and February; spring, March, April and May; summer, June, July and August; and fall, September, October and November. Month of conception was calculated by subtracting gestational week from the date of birth.

Statistical Analyses

First, bivariate analyses were conducted between month of birth and other covariates previously reported to be associated with increased risk of autism [21 22]. The covariates included gestational age in week as a continuous variable [23], birth weight, older maternal or paternal age (>35 years), and SES level above median (>6). Gestational age was available for 97264 individuals (90% of study population). Missing gestational ages were imputed using multiple imputation (5 imputations) with birth weight as a covariate. The Cochran-Armitage test was used to assess a significant trend in the association between month of birth and ASD.

Multivariable logistic regression analyses were performed to determine the odds ratio (OR) and 95% confidence interval CI for ASD by month of birth, using January as the reference month. For the purpose of comparison with previous studies, we fitted alternative logistic regression models, with season of birth, month and season of conception (rather than birth), and the month of the second and first trimester as the main independent variables. Additionally, to evaluate the potential effect modification by socio-economic level, the study population was divided into two groups in further analyses: low-medium (1-6) and medium-high (7-10). Effect modification by month of birth and SES was investigated by including a cross-product interaction term between

For peer review only - http://bmjopen.bmj.com/site/about/guidelines.xhtml

the exposure variable (birth in August X SES group). A *P*-value for interaction[24] was computed to determine the statistical significance of adjusted odds ratios related to birth in August between high and low SES level using WINPEPI statistical packag[25].

In a sensitivity analysis, using conception month and season, we excluded one calendar year at a time to identify the effect of one specific year, with no significant results.

A two-tailed P value of <0.05 was considered statistically significant. All data processing and statistical analyses were performed using IBM Corp. Released 2013. IBM SPSS Statistics for Windows, Version 22.0. Armonk, NY: IBM Corp.).

Results

The mean number of monthly births ranged from 8318 in February to 9654 in August. In bivariate analyses, number of births, maternal age, paternal age, birth weight, gestational length and SES were not associated with the month of birth (Table 1). Of the 108,548 boys born during the study period, 975 were diagnosed with ASD, representing a prevalence rate of 9.0 per 1000 live births. Mean age at ASD diagnosis was 3.0 years (SE=0.07years). The highest incidences of ASD were recorded among children born in May and August: 10.3 and 10.2 per 1000, respectively, while the lowest incidences were in January and February (7.6 and 8.1 per 1000, respectively) (Table 2). The results by month of conception are shown on Table 3.

In a multivariable model, birth months of August and May were associated with a non-significantly (P=0.16 and P=0.14, respectively) increased odds ratio (OR) (Table 4). When the data were stratified by household socioeconomic level, the birth month of August in low SES was associated with an odds ratio of 1.71 (95% confidence interval: 1.06 – 2.74) of ASD. No other significant results were observed for other birth months or

For peer review only - http://bmjopen.bmj.com/site/about/guidelines.xhtml

For peer review only

in children of higher SES (Table 5 and appendices). There was a borderline effect modification between birth in August and SES level (P=0.07).

Using similar multivariable analyses, no significant (P>0.5) association between ASD and season of birth, season or month of conception.

Discussion

In this retrospective cohort of males born in Israel during a period spanning ten years, children of low SES together with birth in August was associated with an increased likelihood of being diagnosed with ASD. This is the one of the largest studies outside the US to describe the relationship between ASD incidence and month of birth.

The literature with respect to season of birth and risk of ASD is inconsistent. Of the 15 studies (total of 31,777 births) summarized in table 6 (all conducted in the Northern Hemisphere) five studies (total n=23,355) reported an increased risk of ASD among individuals born in August. March was significantly associated with increased risk in eight studies (total n=3008), and three studies (total n=5839) found no significant association between month of birth and ASD occurrence. No other specific birth month was repeatedly reported to be associated with ASD.

There are several mechanisms by which season of birth may affect the developing brain, either directly on the fetus and newborn or indirectly through the mother during pregnancy or the postpartum period. Seasonal environmental factors that could be associated with ASD include viral or other infections[26, 27], nutritional factors[28], and vitamin deficiencies[29]. The outcome of exposure to prenatal viral infection depends on many factors: the variation in susceptibility of the maternal and fetal host, including the maternal immune status, the infecting virus, the strain of virus, the developmental stage of the fetus, the amount of virus reaching the fetus— particularly the CNS and immune

For peer review only - http://bmjopen.bmj.com/site/about/guidelines.xhtml

system, the route of access, genetics, and probably other factors[30]. Maternal viral infection has been cited as the "principal non-genetic cause of autism" [31]. Maternal infections (i.e. rubella, cytomegalovirus, influenza), prolonged fever and maternal inflammation cause changes in a variety of inflammatory cytokines that have been associated with ASD [32, 33]. Depending on the location of immune activation, persistent viral infection could lead to chronically elevated cytokine levels that can be produced directly in the brain or gain access to the CNS by crossing an immature blood-brain barrier (BBB), altering CNS development[34].

A few studies found influenza infection during pregnancy to be a factor that increases risk for infantile autism[26 35] especially in the first trimester [33]. Results from animal models also support an effect of maternal influenza infection on the neurodevelopment of the offspring[36 37]. In this analysis, ASD was related significantly with month of birth but not with month or season of conception and therefore our results do not support this potential etiology. This is in line with recently published results of a large cohort from the US[38].

Potential explanations can be proposed for the observed effect modification of increased risk of ASD among August born of lower SES. These may include higher likelihood of maternal exposure to infections or particulate matter (PM) air pollution that have been connected to increased risk of ASD[39] . A role of vitamin D deficiency in the development of autism has been proposed [29]. Thus, seasonal fluctuations in vitamin D should also be considered. In the northern hemisphere, vitamin D levels are the lowest in winter time [40-42] ; however, a study of healthy volunteers from Israel found no differences in vitamin D deficiency rates between summer and winter[43] .

Strengths of the current study include the investigation of a large and unselected population that is similar in its characteristics (mean maternal age, years of follow up

For peer review only - http://bmjopen.bmj.com/site/about/guidelines.xhtml

etc.) to a large cohort of a recently published US study that examined autism and seasonality[38], the cohort design, the use of systematic data that were collected prospectively, validated source of information of ASD diagnosis, the availability of data on prematurity, and the objective assessment of socioeconomic status. Some limitations should be discussed. We had no information about the ASD disease severity; In one previous report[44], high functioning children with ASD showed an increase in summer births compared with the low functioning children with ASD. In addition, SES assessment was conducted using data on current residential area, which is not necessarily the residential area during the time of pregnancy, nor necessarily identical to individual SES. The children who were born at the end of the year were younger than those born earlier in the year, but this is negligible because they were all at the age of at least six years when the study was done and most autistic children are diagnosed by this age [45]. Our analysis included only males and therefore generalizability to both sexes is limited. Data were unavailable regarding important risk factors such as family history, viral infections, and environmental factors. Further investigations on whether the observed seasonal patterns interplay with genetic and environmental risk factors in the development of ASD may provide clues regarding the etiology of these complex disorders.

For peer review only - http://bmjopen.bmj.com/site/about/guidelines.xhtml

CONCLUSIONS

In line with several previous studies, we found modestly higher likelihood of ASD occurrence among male children of lower SES levels born in August. Month of birth may indicate seasonal causal factors that could be associated with autism. The fact that these patterns appear to differ by socioeconomic status may also be etiologically significant.

For peer review only

CONTRIBUTIONS OF AUTHORS:

HS and GC contributed substantially to the conception of the work, analysis, interpretation of data for the work, drafting the work and final approval of the version to be published. IS contributed substantially to the acquisition, interpretation of data for the work, revising it critically for important intellectual content and final approval of the version to be published.

COMPETING INTERESTS: None Declared.

FUNDING: None.

DATA SHARING STATEMENT: No additional data are available

ETHICS APPROVAL: This study was approved by Assuta Healthcare Systems IRB.

ACKNOWLEDGMENTS

This study is part of the MD thesis by Hila Shalev

References

1. American Psychiatric Association. *Diagnostic and statistical manual of mental disorders*. Arlington, VA: American Psychiatric Association; 2013.
2. Newschaffer CJ, Croen LA, Daniels J, Giarelli E, Grether JK, Levy SE, Mandell DS, Miller LA, Pinto-Martin J, Reaven J, Reynolds AM, Rice CE, Schendel D and Windham GC. The epidemiology of autism spectrum disorders. *Annual review of public health*. 2007;28:235-58.
3. Senecky Y, Chodick G, Diamond G, Lobel D, Drachman R and Inbar D. Time trends in reported autistic spectrum disorders in Israel, 1972-2004. *Isr Med Assoc J*. 2009;11:30-3.
4. Torrey EF, Miller J, Rawlings R and Yolken RH. Seasonal birth patterns of neurological disorders. *Neuroepidemiology*. 2000;19:177-85.
5. Mortensen PB, Pedersen CB, Westergaard T, Wohlfahrt J, Ewald H, Mors O, Andersen PK and Melbye M. Effects of family history and place and season of birth on the risk of schizophrenia. *N Engl J Med*. 1999;340:603-8.
6. Torrey EF, Miller J, Rawlings R and Yolken RH. Seasonality of births in schizophrenia and bipolar disorder: a review of the literature. *Schizophr Res*. 1997;28:1-38.
7. Mick E, Biederman J and Faraone SV. Is season of birth a risk factor for attention-deficit hyperactivity disorder? *J Am Acad Child Adolesc Psychiatry*. 1996;35:1470-6.
8. Liederman J and Flannery KA. Fall conception increases the risk of neurodevelopmental disorder in offspring. *J Clin Exp Neuropsychol*. 1994;16:754-68.
9. Livingston R, Adam BS and Bracha HS. Season of birth and neurodevelopmental disorders: summer birth is associated with dyslexia. *J Am Acad Child Adolesc Psychiatry*. 1993;32:612-6.
10. Castrogiovanni P, Iapichino S, Pacchierotti C and Pieraccini F. Season of birth in psychiatry. A review. *Neuropsychobiology*. 1998;37:175-81.
11. Barak Y, Ring A, Sulkes J, Gabbay U and Elizur A. Season of birth and autistic disorder in Israel. *The American journal of psychiatry*. 1995;152:798-800.
12. Biederman J. Attention-deficit/hyperactivity disorder: a selective overview. *Biol Psychiatry*. 2005;57:1215-20.
13. Biederman J and Faraone SV. Attention-deficit hyperactivity disorder. *Lancet*. 2005;366:237-48.
14. Rappley MD. Clinical practice. Attention deficit-hyperactivity disorder. *N Engl J Med*. 2005;352:165-73.
15. Alaerts K, Swinnen SP and Wenderoth N. Sex differences in autism: a resting-state fMRI investigation of functional brain connectivity in males and females. *Soc Cogn Affect Neurosci*. 2016.
16. Werling DM and Geschwind DH. Sex differences in autism spectrum disorders. *Curr Opin Neurol*. 2013;26:146-53.
17. Werling DM. The role of sex-differential biology in risk for autism spectrum disorder. *Biol Sex Differ*. 2016;7:58.
18. Mitra I, Tsang K, Ladd-Acosta C, Croen LA, Aldinger KA, Hendren RL, Traglia M, Lavillaureix A, Zaitlen N, Oldham MC, Levitt P, Nelson S, Amaral DG, Herz-Picciotto I, Fallin MD and Weiss LA. Pleiotropic Mechanisms Indicated for Sex Differences in Autism. *PLoS Genet*. 2016;12:e1006425.

For peer review only - http://bmjopen.bmj.com/site/about/guidelines.xhtml

19. Davidovitch M, Hemo B, Manning-Courtney P and Fombonne E. Prevalence and incidence of autism spectrum disorder in an Israeli population. *J Autism Dev Disord*. 2013;43:785-93.
20. Israel Central Bureau of Statistics. *Statistical Abstracts of Israel 2011: Census of Population and Housing Publication*. Jerusalem: Israel Bureau of Statistics; 2011.
21. Roberts EM, English PB, Grether JK, Windham GC, Somberg L and Wolff C. Maternal residence near agricultural pesticide applications and autism spectrum disorders among children in the California Central Valley. *Environ Health Perspect*. 2007;115:1482-9.
22. Croen LA, Grether JK and Selvin S. Descriptive epidemiology of autism in a California population: who is at risk? *Journal of autism and developmental disorders*. 2002;32:217-24.
23. Leavey A, Zwaigenbaum L, Heavner K and Burstyn I. Gestational age at birth and risk of autism spectrum disorders in Alberta, Canada. *The Journal of pediatrics*. 2013;162:361-8.
24. Altman DG and Bland JM. Interaction revisited: the difference between two estimates. *BMJ*. 2003;326:219.
25. Abramson, J.H., WINPEPI updated: computer programs for epidemiologists, and their teaching potential. Epidemiologic Perspectives & Innovations [computer program]. Version 8:1; 2011.
26. Deykin EY and MacMahon B. Viral exposure and autism. *Am J Epidemiol*. 1979;109:628-38.
27. Lee BK, Magnusson C, Gardner RM, Blomström Å, Newschaffer CJ, Burstyn I, Karlsson H and Dalman C. Maternal hospitalization with infection during pregnancy and risk of autism spectrum disorders. *Brain Behav Immun*. 2015;44:100-5.
28. Lyall K, Schmidt RJ and Hertz-Picciotto I. Maternal lifestyle and environmental risk factors for autism spectrum disorders. *Int J Epidemiol*. 2014;43:443-64.
29. Grant WB and Soles CM. Epidemiologic evidence supporting the role of maternal vitamin D deficiency as a risk factor for the development of infantile autism. *Dermatoendocrinol*. 2009;1:223-8.
30. Blattner RJ. The role of viruses in congenital defects. *Am J Dis Child*. 1974;128:781-6.
31. Ciaranello AL and Ciaranello RD. The neurobiology of infantile autism. *Annu Rev Neurosci*. 1995;18:101-28.
32. Ornoy A, Weinstein-Fudim L and Ergaz Z. Prenatal factors associated with autism spectrum disorder (ASD). *Reprod Toxicol*. 2015;56:155-69.
33. Atladóttir H, Henriksen TB, Schendel DE and Parner ET. Autism after infection, febrile episodes, and antibiotic use during pregnancy: an exploratory study. *Pediatrics*. 2012;130:e1447-54.
34. Muñoz-Fernández MA and Fresno M. The role of tumour necrosis factor, interleukin 6, interferon-gamma and inducible nitric oxide synthase in the development and pathology of the nervous system. *Prog Neurobiol*. 1998;56:307-40.
35. Zhang X, Lv CC, Tian J, Miao RJ, Xi W, Hertz-Picciotto I and Qi L. Prenatal and perinatal risk factors for autism in China. *Journal of autism and developmental disorders*. 2010;40:1311-21.
36. Fatemi SH, Earle J, Kanodia R, Kist D, Emamian ES, Patterson PH, Shi L and Sidwell R. Prenatal viral infection leads to pyramidal cell atrophy and macrocephaly in adulthood: implications for genesis of autism and schizophrenia. *Cell Mol Neurobiol*. 2002;22:25-33.

For peer review only - http://bmjopen.bmj.com/site/about/guidelines.xhtml

37. Shi L, Fatemi SH, Sidwell RW and Patterson PH. Maternal influenza infection causes marked behavioral and pharmacological changes in the offspring. *J Neurosci*. 2003;23:297-302.
38. Zerbo O, Qian Y, Yoshida C, Fireman BH, Klein NP and Croen LA. Association Between Influenza Infection and Vaccination During Pregnancy and Risk of Autism Spectrum Disorder. *JAMA Pediatr*. 2017;171:e163609.
39. Raz R, Roberts AL, Lyall K, Hart JE, Just AC, Laden F and Weisskopf MG. Autism spectrum disorder and particulate matter air pollution before, during, and after pregnancy: a nested case-control analysis within the Nurses' Health Study II Cohort. *Environ Health Perspect*. 2015;123:264-70.
40. Kasahara AK, Singh RJ and Noymer A. Vitamin D (25OHD) Serum Seasonality in the United States. *PLoS One*. 2013;8:e65785.
41. Kashi Z, Saeedian F, Akha O, Gorgi M, Emadi S and Zakeri H. Vitamin D deficiency prevalence in summer compared to winter in a city with high humidity and a sultry climate. *Endokrynol Pol*. 2011;62:249-51.
42. Costanzo PR, Constanzo PR, Elías NO, Kleiman Rubinsztein J, García Basavilbaso NX, Piacentini R and Salerni HH. [Ultraviolet radiation impact on seasonal variations of serum 25-hydroxy-vitamin D in healthy young adults in Buenos Aires]. *Medicina (B Aires)*. 2011;71:336-42.
43. Oren Y, Shapira Y, Agmon-Levin N, Kivity S, Zafrir Y, Altman A, Lerner A and Shoenfeld Y. Vitamin D insufficiency in a sunny environment: a demographic and seasonal analysis. *Isr Med Assoc J*. 2010;12:751-6.
44. Marvin K. Season of Birth Effects in Autism Spectrum Disorders. *Honors Scholar Theses*. 2008;58.
45. Hertz-Picciotto I and Delwiche L. The rise in autism and the role of age at diagnosis. *Epidemiology*. 2009;20:84-90.
46. Zerbo O, Iosif AM, Delwiche L, Walker C and Hertz-Picciotto I. Month of conception and risk of autism. *Epidemiology*. 2011;22:469-75.
47. Mazumdar S, Liu KY, Susser E and Bearman P. The disappearing seasonality of autism conceptions in California. *PLoS One*. 2012;7:e41265.
48. Bolton P, Pickles A, Harrington R, Macdonald H and Rutter M. Season of birth: issues, approaches and findings for autism. *Journal of child psychology and psychiatry, and allied disciplines*. 1992;33:509-30.
49. Lee LC, Newschaffer CJ, Lessler JT, Lee BK, Shah R and Zimmerman AW. Variation in season of birth in singleton and multiple births concordant for autism spectrum disorders. *Paediatr Perinat Epidemiol*. 2008;22:172-9.
50. Bartlik BD. Monthly variation in births of autistic children in North Carolina. *Journal of the American Medical Women's Association (1972)*. 1981;36:363-8.
51. Landau EC, Cicchetti DV, Klin A and Volkmar FR. Season of birth in autism: a fiction revisited. *J Autism Dev Disord*. 1999;29:385-93.
52. Mouridsen SE, Nielsen S, Rich B and Isager T. Season of birth in infantile autism and other types of childhood psychoses. *Child psychiatry and human development*. 1994;25:31-43.
53. Kolevzon A, Weiser M, Gross R, Lubin G, Knobler HY, Schmeidler J, Silverman JM and Reichenberg A. Effects of season of birth on autism spectrum disorders: fact or fiction? *The American journal of psychiatry*. 2006;163:1288-90.
54. Konstantareas MM, Hauser P, Lennox C and Homatidis S. Season of birth in infantile autism. *Child psychiatry and human development*. 1986;17:53-65.

For peer review only - http://bmjopen.bmj.com/site/about/guidelines.xhtml

55. Stevens MC, Fein DH and Waterhouse LH. Season of birth effects in autism. *J Clin Exp Neuropsychol*. 2000;22:399-407.
56. Gillberg C. Do children with autism have March birthdays? *Acta Psychiatr Scand*. 1990;82:152-6.
57. Tanoue Y, Oda S, Asano F and Kawashima K. Epidemiology of infantile autism in southern Ibaraki, Japan: differences in prevalence in birth cohorts. *Journal of autism and developmental disorders*. 1988;18:155-66.
58. Hebert KJ, Miller LL and Joinson CJ. Association of autistic spectrum disorder with season of birth and conception in a UK cohort. *Autism research : official journal of the International Society for Autism Research*. 2010;3:185-90.

For peer review only

For peer review only - http://bmjopen.bmj.com/site/about/guidelines.xhtml

Table 1 Study population characteristics by month of birth

Month	Births	SES* 1 to 10				Gestational Age				Birth weight, g				Paternal age				Maternal age			
		75%	25%	Med	M (SD)	75%	25%	Med	M (SD)	75%	25%	Med	M (SD)	75%	25%	Med	M (SD)	75%	25%	Med	M (SD)
Jan	*9367*	8	4	6	6.1 (2.3)	40	38	39	39.1 (1.9)	3630	3000	3320	3296 (525)	36	29	33	32.9 (5.7)	33	*27*	*30*	30.1 (4.9)
Feb	*8318*	8	5	6	6.2 (2.3)	40	38	39	39.0 (2.0)	3640	3000	3320	3296 (528)	36	29	33	32.9 (5.7)	34	*27*	*30*	30.2 (4.9)
Mar	*9038*	8	4	6	6.1 (2.3)	40	38	39	39.0 (1.9)	3650	3020	3340	3305 (519)	36	29	33	32.9 (5.6)	34	*27*	*30*	30.2 (4.9)
Apr	*8465*	8	5	6	6.2 (2.3)	40	38	39	39.1 (1.9)	3640	3030	3340	3310 (519)	37	29	33	33.0 (5.6)	34	*27*	*30*	30.3 (4.9)
May	*8711*	8	5	6	6.3 (2.3)	40	38	39	39.1 (1.9)	3640	3010	3330	3300 (532)	37	29	33	33.0 (5.6)	34	*27*	*30*	30.3 (4.9)
Jun	*8523*	8	5	6	6.2 (2.3)	40	38	39	39.0 (2.0)	3650	3010	3350	3306 (538)	37	29	33	33.1 (5.6)	34	*27*	*30*	30.3 (5.0)
Jul	*9330*	8	4	6	6.1 (2.3)	40	38	39	39.0 (2.0)	3650	3010	3330	3304 (528)	37	29	33	33.0 (5.7)	34	*27*	*30*	30.2 (4.9)
Aug	*9654*	8	4	6	6.1 (2.3)	40	38	39	39.0 (1.9)	3660	3020	3340	3312 (532)	37	29	33	33.1 (5.7)	34	*27*	*30*	30.3 (4.9)
Sep	*9270*	8	4	6	6.1 (2.3)	40	38	39	39.0 (1.9)	3640	3000	3320	3291 (537)	37	29	33	33.1 (5.7)	34	*27*	*30*	30.3 (4.9)
Oct	*9521*	8	4	6	6.1 (2.3)	40	38	39	39.1 (1.9)	3640	3020	3330	3302 (524)	37	29	33	33.0 (5.7)	34	*27*	*30*	30.3 (5.0)
Nov	*9069*	8	4	6	6.1 (2.3)	40	38	39	39.0 (1.9)	3640	3000	3330	3302 (532)	37	29	33	33.1 (5.7)	34	*27*	*30*	30.3 (5.0)
Dec	*9282*	8	4	6	6.1 (2.3)	40	38	39	39.0 (2.0)	3630	3000	3320	3288 (540)	37	29	33	33.0 (5.7)	34	*27*	*30*	30.3 (5.0)
**Total**	***108548***	**8**	**4.33**	**6**	**6.1 (2.3)**	**39.0 (2.0)**	**38**	**39**	**39.0 (2.0)**	**3301 (529.5)**	**3010**	**3330.8**	**3301 (529.5)**	**33.0 (5.7)**	**29**	**33**	**33.0 (5.7)**	**33.9**	***27***	***30***	**30.3 (5)**

- See text for details on definitions of SES

For peer review only - http://bmjopen.bmj.com/site/about/guidelines.xhtml

Table 2: Incidence rate and 95% confidence intervals (95%CI) per 1000 male live births of Autistic spectrum disorder (ASD), by month of birth

Month of birth	ASD cases	Rate (per 1000)	95% CI*	
Jan	76	8.1	6.44	10.09
Feb	63	7.6	5.87	9.62
Mar	74	8.2	6.48	10.21
Apr	74	8.7	6.92	10.90
May	90	10.3	8.36	12.62
Jun	78	9.2	7.29	11.35
Jul	89	9.5	7.71	11.67
Aug	98	10.2	8.29	12.30
Sep	80	8.6	6.89	10.67
Oct	85	8.9	7.18	10.97
Nov	84	9.3	7.44	11.40
Dec	84	9.0	7.27	11.14
Total	975	8.96	7.17	11.07

* see text for details

For peer review only

For peer review only - http://bmjopen.bmj.com/site/about/guidelines.xhtml

Table 3: Incidence rate and 95% confidence intervals (95%CI) per 1000 male live births of Autistic spectrum disorder (ASD), by month of conception

Month of conception	ASD cases	Rate (per 1000)	95% CI*	
Jan	87	9.2	7.4	11.3
Feb	74	8.7	6.9	11
Mar	77	8.3	6.6	10.4
Apr	72	8.0	6.3	10.1
May	77	8.4	6.7	10.5
Jun	68	7.8	6.1	9.9
Jul	72	8.2	6.4	10.3
Aug	89	10.2	8.2	12.5
Sep	91	10.9	8.8	13.4
Oct	79	8.4	6.6	10.4
Nov	99	10.8	8.8	13.1
Dec	87	9.1	7.3	11.2
Total	972	9.0	8.4	9.9

* see text for details

For peer review only

For peer review only - http://bmjopen.bmj.com/site/about/guidelines.xhtml

Table 4: Crude and adjusted odd ratio (OR) and 95% confidence interval of autistic spectrum disorder (ASD) for month of birth: multivariable logistic regression

Month of birth	OR crude	95%CI		OR adjusted*	95%CI	
Jan	1 (ref.)			1 (ref.		
Feb	.93	.67	1.31	.92	.66	1.29
Mar	1.01	.73	1.39	1.00	.73	1.38
Apr	1.08	.78	1.49	1.08	.78	1.48
May	1.28	.94	1.73	1.26	.92	1.71
Jun	1.13	.82	1.55	1.11	.81	1.52
Jul	1.16	.86	1.58	1.15	.84	1.56
Aug	1.25	.93	1.69	1.24	.92	1.68
Sep	1.07	.78	1.46	1.05	.77	1.44
Oct	1.10	.81	1.50	1.09	.80	1.49
Nov	1.13	.83	1.55	1.12	.82	1.53
Dec	1.11	.81	1.51	1.09	.80	1.50

*Adjusted for child's age, father's age, mother's age, district, birth weight, gestational age with imputation (see text for details).

For peer review only

For peer review only - http://bmjopen.bmj.com/site/about/guidelines.xhtml

Table 5: Crude and adjusted odd ratio (OR) and 95% confidence interval from multivariable logistic regression of autistic spectrum disorder (ASD) for month of birth, by socioeconomic level

	SES 1-6						SES 7-10					
**Month of birth**	**OR crude**	**95%CI**		**OR adjusted***	**95%CI**		**OR crude**	**95%CI**		**OR adjusted***	**95%CI**	
Jan	1 (ref.)			1 (ref.)			1 (ref.)			1 (ref.)		
Feb	1.01	0.58	1.75	1.01	0.58	1.75	0.89	0.58	1.35	0.88	0.57	1.34
Mar	1.34	0.80	2.22	1.34	0.81	2.22	0.82	0.54	1.25	0.81	0.53	1.24
Apr	1.30	0.77	2.19	1.32	0.78	2.22	0.93	0.62	1.40	0.93	0.62	1.40
May	1.31	0.78	2.20	1.30	0.78	2.19	1.19	0.81	1.74	1.17	0.80	1.72
Jun	1.26	0.75	2.12	1.25	0.74	2.11	1.01	0.67	1.51	0.99	0.66	1.49
Jul	1.37	0.83	2.25	1.36	0.82	2.25	1.01	0.68	1.49	0.99	0.67	1.48
Aug	1.70	1.06	2.73	1.71	1.07	2.76	1.00	0.67	1.48	0.99	0.66	1.46
Sep	1.32	0.80	2.19	1.32	0.80	2.18	0.92	0.61	1.38	0.91	0.60	1.36
Oct	1.24	0.75	2.05	1.24	0.75	2.07	1.03	0.69	1.52	1.01	0.68	1.50
Nov	1.45	0.88	2.37	1.46	0.89	2.40	0.96	0.64	1.44	0.93	0.62	1.40
Dec	1.49	0.91	2.43	1.51	0.92	2.46	0.89	0.59	1.34	0.88	0.58	1.32

*Adjusted for child's age, father's age, mother's age, district, birth weight, age with imputation (see text for details).

For peer review only

For peer review only - http://bmjopen.bmj.com/site/about/guidelines.xhtml

Table 6: Summary of Findings in Previous Studies Investigating the Relationship Between Seasonal Variations of Birth and Autism

- Conception in November (For years 1994 – 1996) ** (P<0.1 for middle of August for verbal ASD) *** year of publication

Ref.	Study period	**Place**	ASD cases	Months of increased risk of ASD
46	1990 – 2002	California	19,238	Nov, Dec, Aug, Sep, Oct
47	1992 – 2000	California	2,940	Aug*
47	1997 – 1999	California	3,897	No Association
48	1947 – 1980	UK	1631	No Association
49	1983 – 2002	Maryland	1068	Spring
50	1981***	N Carolina	810	Mar ,Aug
51	1947-1992	Child dev. clinic	620	No Association**
52	1945 – 1980	Denmark	328	March
53	1980 – 1985	Israel	211	No Association
11	1964 – 1986	Israel	188	March and August
54	1980s	Ottario ,Canada	179	Summer and Spring
55	1977 – 1985	US	175	March
56	1962 – 1984	Sweden	174	March
57	1972 – 1978	Japan	132	Spring-Summer (Apr, May, Jun)
58	1990–1992	UK	86	Spring

For peer review only

For peer review only

For peer review only - http://bmjopen.bmj.com/site/about/guidelines.xhtml

Appendix

Table a: Crude and adjusted odd ratio (OR) and 95% confidence interval from multivariable logistic regression of autistic spectrum disorder (ASD) for month of conception, by socioeconomic status (SES)

	SES 1-6						SES 7-10					
**Month of conception**	**OR crude**	**95%CI**		**OR adjusted***	**95%CI**		**OR crude**	**95%CI**		**OR adjusted***	**95%CI**	
Jan	1 (ref.)			1 (ref.			1 (ref.)			1 (ref.		
Feb	.89	.52	1.50	.89	.52	1.51	1.09	.72	1.65	1.08	.72	1.63
Mar	1.13	.70	1.85	1.14	.70	1.86	.84	.55	1.30	.84	.54	1.29
Apr	.90	.53	1.53	.89	.53	1.52	1.06	.71	1.60	1.09	.72	1.64
May	1.05	.63	1.75	1.03	.62	1.71	.94	.62	1.44	.95	.62	1.45
Jun	1.19	.72	1.96	1.17	.71	1.93	.74	.46	1.16	.75	.47	1.18
Jul	.79	.45	1.38	.79	.45	1.38	.96	.63	1.45	.97	.64	1.47
Aug	1.11	.67	1.83	1.09	.66	1.81	1.13	.76	1.68	1.13	.76	1.68
Sep	1.29	.79	2.10	1.28	.78	2.08	1.09	.73	1.64	1.10	.73	1.65
Oct	.95	.57	1.58	.96	.58	1.59	.88	.58	1.34	.89	.58	1.35
Nov	1.14	.70	1.86	1.14	.70	1.86	1.16	.78	1.72	1.16	.78	1.72
Dec	1.18	.74	1.91	1.18	.74	1.91	.90	.59	1.36	.89	.59	1.36

*Adjusted for child's age, father's age, mother's age, district, birth weight, age with imputation (see text for details).

For peer review only

Table b: Crude and adjusted odd ratio (OR) and 95% confidence interval from multivariable logistic regression of autistic spectrum disorder (ASD) for season of birth, by socioeconomic status (SES)

	SES median-high						SES median-low					
**Season of birth**	**OR crude**	**95%CI**		**OR adjusted***	**95%CI**		**OR crude**	**95%CI**		**OR adjusted***	**95%CI**	
Winter	1 (ref.)			1 (ref.			1 (ref.)			1 (ref.		
Spring	1.12	0.84	1.50	1.12	0.84	1.50	1.06	0.83	1.34	1.06	0.83	1.34
Summer	1.24	0.94	1.63	1.23	0.93	1.63	1.08	0.85	1.37	1.08	0.85	1.37
Fall	1.14	0.86	1.51	1.14	0.86	1.51	1.05	0.82	1.33	1.04	0.82	1.32

*Adjusted for child's age, father's age, mother's age, district, birth weight, gestational age with imputation

For peer review only

Table c: Crude and adjusted odd ratio (OR) and 95% confidence interval from multivariable logistic regression of autistic spectrum disorder (ASD) for season of conception, by socioeconomic status (SES)

	SES median-high						SES median-low					
**Season of conception**	**OR crude**	**95%CI**		**OR adjusted***	**95%CI**		**OR crude**	**95%CI**		**OR adjusted***	**95%CI**	
**Winter**	1 (ref.)			1 (ref.			1 (ref.)			1 (ref.		
**Spring**	1.00	0.75	1.34	0.99	0.74	1.33	0.96	0.75	1.22	0.96	0.75	1.23
**Summer**	1.00	0.74	1.35	0.99	0.73	1.33	0.96	0.75	1.22	0.97	0.76	1.24
**Fall**	1.09	0.82	1.45	1.08	0.82	1.44	1.05	0.83	1.33	1.06	0.83	1.34

*Adjusted for child's age, father's age, mother's age, district, birth weight, gestational age with imputation

For peer review only

Table d: Crude and adjusted odd ratio (OR) and 95% confidence interval from multivariable logistic regression of autistic spectrum disorder (ASD) for first month of second trimester, by socioeconomic status (SES)

Month of second trimester	OR crude	95%CI		OR adjusted*	95%CI		OR crude	95%CI		OR adjusted*	95%CI	
**Jan**	1 (ref.)			1 (ref.			1 (ref.)			1 (ref.		
**Feb**	.83	.51	1.35	.83	.51	1.36	1.03	.67	1.57	1.03	.67	1.57
**Mar**	1.08	.69	1.69	1.08	.69	1.69	.87	.57	1.35	.86	.56	1.33
**Apr**	.89	.55	1.43	.89	.55	1.43	1.12	.74	1.69	1.11	.73	1.67
**May**	.68	.41	1.12	.68	.41	1.13	.99	.65	1.51	.98	.64	1.49
**Jun**	.87	.53	1.41	.87	.54	1.41	1.09	.72	1.64	1.07	.71	1.63
**Jul**	1.00	.62	1.59	.98	.61	1.56	1.02	.67	1.56	1.04	.68	1.59
**Aug**	.74	.44	1.23	.72	.43	1.20	.97	.63	1.49	.97	.63	1.49
**Sep**	.94	.58	1.54	.93	.57	1.51	.86	.55	1.35	.87	.55	1.36
**Oct**	.84	.51	1.37	.83	.51	1.37	1.10	.73	1.66	1.11	.73	1.67
**Nov**	.84	.51	1.40	.83	.50	1.38	1.25	.84	1.87	1.24	.83	1.86
**Dec**	.83	.51	1.36	.83	.51	1.36	1.10	.73	1.65	1.09	.72	1.64

*Adjusted for child's age, father's age, mother's age, district, birth weight, age with imputation (see text for details).

For peer review only

Table d: Crude and adjusted odd ratio (OR) and 95% confidence interval from multivariable logistic regression of autistic spectrum disorder (ASD) for first month of third trimester, by socioeconomic status (SES)

Month of third trimester	OR crude	95%CI		OR adjusted*	95%CI		OR crude	95%CI		OR adjusted*	95%CI	
Jan	1 (ref.)			1 (ref.			1 (ref.)			1 (ref.		
Feb	1.04	0.61	1.77	1.04	0.61	1.78	1.22	0.81	1.82	1.21	0.81	1.81
Mar	0.97	0.57	1.63	0.97	0.58	1.64	0.96	0.63	1.46	0.95	0.63	1.45
Apr	1.19	0.73	1.95	1.20	0.74	1.97	1.13	0.75	1.68	1.12	0.75	1.68
May	0.86	0.51	1.46	0.87	0.52	1.48	0.86	0.56	1.32	0.86	0.56	1.31
Jun	1.26	0.77	2.04	1.27	0.78	2.06	0.84	0.55	1.30	0.83	0.54	1.27
Jul	0.99	0.60	1.65	1.01	0.61	1.67	1.07	0.71	1.61	1.05	0.70	1.58
Aug	0.78	0.45	1.33	0.79	0.46	1.36	0.99	0.66	1.51	0.98	0.64	1.48
Sep	1.12	0.68	1.86	1.12	0.68	1.86	1.12	0.75	1.69	1.12	0.74	1.69
Oct	0.84	0.49	1.44	0.83	0.48	1.42	0.83	0.53	1.29	0.83	0.54	1.30
Nov	0.92	0.53	1.57	0.91	0.53	1.56	1.00	0.65	1.53	1.00	0.65	1.54
Dec	1.07	0.64	1.80	1.08	0.64	1.81	0.81	0.52	1.26	0.81	0.52	1.26

*Adjusted for child's age, father's age, mother's age, district, birth weight, age with imputation (see text for details).

For peer review only

STROBE 2007 (v4) checklist of items to be included in reports of observational studies in epidemiology*
Checklist for cohort, case-control, and cross-sectional studies (combined)

Section/Topic	Item #	Recommendation	Reported on page # (clean version)
**Title and abstract**	1	(*a*) Indicate the study's design with a commonly used term in the title or the abstract	2
		(*b*) Provide in the abstract an informative and balanced summary of what was done and what was found	2
**Introduction**			
Background/rationale	2	Explain the scientific background and rationale for the investigation being reported	4th page second and third paragraph, 5th page first paragraph
Objectives	3	State specific objectives, including any pre-specified hypotheses	4th page third paragraph
**Methods**			
Study design	4	Present key elements of study design early in the paper	5 last paragraph through page 6 last paragraph
Setting	5	Describe the setting, locations, and relevant dates, including periods of recruitment, exposure, follow-up, and data collection	6 first paragraph
Participants	6	(*a*) *Cohort study*—Give the eligibility criteria, and the sources and methods of selection of participants. Describe methods of follow-up<br>*Case-control study*—Give the eligibility criteria, and the sources and methods of case ascertainment and control selection. Give the rationale for the choice of cases and controls<br>*Cross-sectional study*—Give the eligibility criteria, and the sources and methods of selection of participants	5 last paragraph through page 6 second paragraph (including)
		(*b*) *Cohort study*—For matched studies, give matching criteria and number of exposed and unexposed<br>*Case-control study*—For matched studies, give matching criteria and the number of controls per case	-
Variables	7	Clearly define all outcomes, exposures, predictors, potential confounders, and effect modifiers. Give diagnostic criteria, if applicable	6 – study population and case identification and matching.
Data sources/ measurement	8*	For each variable of interest, give sources of data and details of methods of assessment (measurement). Describe comparability of assessment methods if there is more than one group	*6 last paragraph, 7 and first paragraph*

For peer review only

			of page 8
Bias	9	Describe any efforts to address potential sources of bias	7 last paragraph through 8 first paragraph (imputation for GA, multivariable analysis, stratification by SES, validation of ASD cases.)
Study size	10	Explain how the study size was arrived at	6
Quantitative variables	11	Explain how quantitative variables were handled in the analyses. If applicable, describe which groupings were chosen and why	6
Statistical methods	12	(*a*) Describe all statistical methods, including those used to control for confounding	6
		(*b*) Describe any methods used to examine subgroups and interactions	6
		(*c*) Explain how missing data were addressed	6 first paragraph
		(*d*) *Cohort study*—If applicable, explain how loss to follow-up was addressed<br>*Case-control study*—If applicable, explain how matching of cases and controls was addressed<br>*Cross-sectional study*—If applicable, describe analytical methods taking account of sampling strategy	6 2nd paragraph
		(*e*) Describe any sensitivity analyses	8 first paragraph (Using Conception month and season, excluding one calendar year at a time to see effect of one specific year)
**Results**			
Participants	13*	(a) Report numbers of individuals at each stage of study—eg numbers potentially eligible, examined for eligibility, confirmed eligible, included in the study, completing follow-up, and analysed	6 first paragraph
		(b) Give reasons for non-participation at each stage	6 first paragraph
		(c) Consider use of a flow diagram	-
Descriptive data	14*	(a) Give characteristics of study participants (eg demographic, clinical, social) and information on exposures and potential confounders	6 last paragraph

For peer review only

		(b) Indicate number of participants with missing data for each variable of interest	6 first paragraph
		(c) *Cohort study*—Summarise follow-up time (eg, average and total amount)	-
Outcome data	15*	*Cohort study*—Report numbers of outcome events or summary measures over time	-
		Case-control study—Report numbers in each exposure category, or summary measures of exposure	6 first paragraph
		Cross-sectional study—Report numbers of outcome events or summary measures	-
Main results	16	(a)Give unadjusted estimates and, if applicable, confounder-adjusted estimates and their precision (eg, 95% confidence interval). Make clear which confounders were adjusted for and why they were included	Tables 5 and 6 (pages 21 and 22)
		(*b*) Report category boundaries when continuous variables were categorized	6-7
		(*c*) If relevant, consider translating estimates of relative risk into absolute risk for a meaningful time period	Table 6 (page 22)
Other analyses	17	Report other analyses done—eg analyses of subgroups and interactions, and sensitivity analyses	7 through first paragraph of page 8
**Discussion**			
Key results	18	Summarise key results with reference to study objectives	8+table 5 (page 21)
Limitations	19	Discuss limitations of the study, taking into account sources of potential bias or imprecision. Discuss both direction and magnitude of any potential bias	11
Interpretation	20	Give a cautious overall interpretation of results considering objectives, limitations, multiplicity of analyses, results from similar studies, and other relevant evidence	9+table 6 (page 22)
Generalisability	21	Discuss the generalisability (external validity) of the study results	9 2nd paragraph (lines 13,14)- (Our results are similar to previous studies)
**Other information**			
Funding	22	Give the source of funding and the role of the funders for the present study and, if applicable, for the original study on which the present article is based	Not Relevant

*Give information separately for cases and controls in case-control studies and, if applicable, for exposed and unexposed groups in cohort and cross-sectional studies.

Note: An Explanation and Elaboration article discusses each checklist item and gives methodological background and published examples of transparent reporting. The STROBE checklist is best used in conjunction with this article (freely available on the Web sites of PLoS Medicine at http://www.plosmedicine.org/, Annals of Internal Medicine at http://www.annals.org/, and Epidemiology at http://www.epidem.com/). Information on the STROBE Initiative is available at www.strobe-statement.org.

For peer review only

BMJ Open

Month of birth and risk of autism spectrum disorder, a retrospective cohort of male children born in Israel

Journal:	*BMJ Open*
Manuscript ID	bmjopen-2016-014606.R3
Article Type:	Research
Date Submitted by the Author:	27-Mar-2017
Complete List of Authors:	Shalev, Hila; Technion Israel Institute of Technology Ruth and Bruce Rappaport Faculty of Medicine, Medicine<br>Solt, Ido; Technion Israel Institute of Technology Ruth and Bruce Rappaport Faculty of Medicine, Medicine; Rambam Health Care Campus<br>Chodick, Gabriel ; Sackler School of Medicine Tel Aviv University, Ramat Aviv, Israel, Epidemiology and Database Research
**Primary Subject Heading**:	Epidemiology
Secondary Subject Heading:	Paediatrics, Public health
Keywords:	EPIDEMIOLOGY, Paediatric neurology < NEUROLOGY, Maternal medicine < OBSTETRICS, Developmental neurology & neurodisability < PAEDIATRICS

SCHOLARONE™
Manuscripts

For peer review only

Development of a core outcome set for clinical trials in inflammatory bowel disease: study protocol for a systematic review of the literature and identification of a core outcome set using a Delphi survey

Authors:

Christopher Ma[1], Remo Panaccione[1], Richard N. Fedorak[2], Claire E. Parker[3], Reena Khanna[3,4], Barrett G. Levesque[3,5], William J. Sandborn[3,5], Brian G. Feagan[3,4,6], and Vipul Jairath[3,4,6]

Affiliations:

[1] Division of Gastroenterology and Hepatology, University of Calgary, Calgary, Alberta, Canada

[2] Division of Gastroenterology, University of Alberta, Edmonton, Alberta, Canada

[3] Robarts Clinical Trials, Western University, London, Ontario, Canada

[4] Department of Medicine, Western University, London, Ontario, Canada

[5] Division of Gastroenterology, University of California San Diego, La Jolla, California, United States

[6] Department of Epidemiology and Biostatistics, Western University, London, Ontario, Canada

For peer review only

Manuscript Word Count: 3716

Abstract Word Count: 282

Number of Tables: 0

Number of Figures: 0

Number of Supplemental Files: 2

Short Title: Development of a core outcome set for IBD clinical trials

Funding Support: None

Corresponding Author:
Dr. Vipul Jairath
Associate Professor of Medicine
Departments of Medicine and Epidemiology and Biostatistics
Western University
Robarts Clinical Trials
Suite 200, 100 Dundas Street
London, Ontario, Canada
N6A 5B6

Phone: 519-685-8500
Fax: 519-663-3658
Email: vipul.jairath@robartsinc.com

Version: April 24, 2017

For peer review only

ABSTRACT

Introduction:

Crohn's disease (CD) and ulcerative colitis (UC), the main forms of inflammatory bowel disease (IBD), are chronic, progressive, and disabling disorders of the gastrointestinal tract. Although data from randomized controlled trials (RCTs) provide the foundation of evidence that validates medical therapy for IBD, considerable heterogeneity exists in the measured outcomes used in these studies. Furthermore, in recent years, there has been a paradigm shift in IBD treatment targets, moving from symptom-based scoring to improvement or normalization of objective measures of inflammation such as endoscopic appearance, inflammatory biomarkers, and histologic and radiographic endpoints. The abundance of new treatment options and evolving endpoints poses opportunities and challenges for all stakeholders involved in drug development. Accordingly, there exists a need to harmonize measures used in clinical trials through development of a core outcome set (COS).

Methods and Analysis:

The development of an IBD-specific COS includes four steps. First, a systematic literature review is performed to identify outcomes previously used in IBD RCTs. Second, semi-structured qualitative interviews are conducted with key stakeholders, including patients, clinicians, researchers, pharmaceutical industry representatives, health care payers, and regulators to identify additional outcomes of importance. Using the outcomes generated from literature review and stakeholder interviews, an international two-round Delphi survey is conducted to prioritize outcomes for inclusion in

For peer review only

For peer review only - http://bmjopen.bmj.com/site/about/guidelines.xhtml

the COS. Finally, a consensus meeting is held to ratify the COS and disseminate findings for application in future IBD trials.

Ethics and Dissemination:

Given that over 30 novel therapeutic compounds are in development for IBD treatment, the design of robust clinical trials measuring relevant and standardized outcomes is crucial. Standardizing outcomes through a COS will reduce heterogeneity in trial reporting, facilitate valid comparisons of new therapies, and improve clinical trial quality.

Keywords:

Inflammatory bowel disease, Crohn's disease, ulcerative colitis, core outcome set, systematic review, consensus methods, Delphi

STRENGTHS AND LIMITATIONS

- This protocol outlines the first international consensus effort to develop a core outcome set (COS) for use in IBD clinical trials. With over 30 novel therapeutic compounds in development for IBD treatment and rapidly evolving treatment targets, the need to harmonize clinical trial efficacy and safety outcomes in a COS is exigent.
- The multistep process to develop the COS is rigorous and involves a detailed systematic literature review, semi-structured interviews with key stakeholder groups, two-round Delphi survey to prioritize key outcomes, and a consensus meeting to ratify the COS.

For peer review only

- To develop the COS, we will seek input from multiple stakeholders, including patients, clinicians, researchers, pharmaceutical industry representatives, health care payers, and regulators. This will generate diverse viewpoints reflecting clinical practices from around the world.
- Although the scope of this COS will be focused towards use in prospective clinical trials in IBD, the selected outcomes may not be relevant for open-label or retrospective studies of IBD treatment

For peer review only - http://bmjopen.bmj.com/site/about/guidelines.xhtml

INTRODUCTION

The inflammatory bowel diseases (IBD), Crohn's disease (CD) and ulcerative colitis (UC), are chronic, progressive, and often disabling disorders of the gastrointestinal tract with no cure. Worldwide, the incidence of IBD is increasing with the highest incidence in North America and Europe; however rapidly rising rates of disease in Asia[1] have recently been observed. Typical symptoms of these diseases, which include diarrhea, gastrointestinal bleeding, and abdominal pain, cause impaired quality of life, reduced work capacity, and social stigmatization.[2] Although the etiology of IBD is unknown, existing evidence implicates development of a dysregulated immune response in genetically susceptible individuals consequent to complex interactions between the intestinal microbiome and environmental exposures.[3] Both CD and UC are lifelong diseases without a cure that typically require continued medical therapy as well as surgery in a large proportion of patients. Additionally, the direct and indirect costs associated with IBD is estimated to exceed $30 billion annually in the United States alone.[4 5]

Treatment of CD and UC is focused on controlling inflammation with anti-inflammatory and immunosuppressive agents, with goals of induction and maintenance of remission. In particular, the adoption of biologic therapies over the past two decades has revolutionized IBD management, making sustained remission an achievable therapeutic target.[6] Approval of these new agents has relied upon data from robust randomized controlled trials (RCTs)[7-14] that in recent years have increased in size and sophistication. Advances in this field continue at an increasingly rapid pace with multiple

For peer review only

For peer review only - http://bmjopen.bmj.com/site/about/guidelines.xhtml

classes of agents in late phase development.[15 16] In parallel, a paradigm shift in treatment targets for IBD has occurred, with a move away from symptom-based scoring[17-19] to normalization of more objective measures of inflammation such as endoscopic appearance, inflammatory biomarkers, and histologic and radiographic endpoints.

Furthermore, recognizing the need to accurately measure the patient experience with IBD, the US Food and Drug Administration (FDA) has advocated for measurement of patient-reported outcomes (PROs) in clinical trials.[20] The utilization of PROs as a treatment endpoint in IBD trials poses unique challenges: importantly, symptom scoring is likely to remain a central component of IBD PROs, despite poor sensitivity and specificity for predicting mucosal inflammation.[21] Symptom scoring may also be confounded by psychological comorbidity and perceived stress,[22] resulting in disparities between PROs and objectively assessed endoscopic, radiographic, and histologic disease activity, especially in Crohn's disease. Thus, the adoption of PROs as a primary therapeutic target in clinical trials would require careful evaluation.

In addition to the shift in efficacy outcomes measured in IBD trials, the assessment of safety outcomes has also changed with the introduction of biologic and immunomodulator therapies, which are often used in combination. As novel treatments are developed to target different components of the immune response, short- and long-term safety evaluations are essential. These include the risks of bacterial infections (including tuberculosis), viral infections (including hepatitis B or herpes zoster virus

For peer review only

For peer review only - http://bmjopen.bmj.com/site/about/guidelines.xhtml

reactivation), malignancy, lymphoma, infusion and injection reactions, and development of anti-drug antibodies.[23]

These shifts in the research environment have led investigators and regulatory authorities to re-evaluate the key efficacy and safety outcomes measured in IBD clinical trials. The selection of appropriate outcomes is critical for several reasons. First, their operating properties determine trial efficiency and ultimately drive both our ability to accurately identify effective new therapies and the cost of dug development programs. Second, choice of outcomes can shape clinical practice if the selected endpoints are perceived to be relevant to both patients and health care professionals. Third, identification of standardized outcomes has potential to facilitate and improve the quality of systematic reviews and meta-analyses. Finally, outcome measures are critical components of the analyses used by payers to determine the safety and relative cost-effectiveness of competing treatments and significantly influence regulatory and formulary policy.[24]

It is apparent that insufficient attention has been paid to the standardized assessment of outcome measures for IBD trials. Notably, no formalized consensus exists regarding what to measure, how to measure, and when to measure selected efficacy and safety outcomes in IBD trials.[25] Given the evolving landscape of IBD treatment endpoints and the rapid development of new therapies, an international consensus agreement on core outcomes for use in future IBD trials is of critical importance.

For peer review only - http://bmjopen.bmj.com/site/about/guidelines.xhtml

A core outcome set (COS) is a consensus derived minimum set of outcomes that should be measured and reported in all clinical trials of a given disease.[24] The expectation is that core outcomes will always be collected and reported, but the COS is not restrictive such that investigators are still encouraged to explore other outcomes in addition to the COS. COS have been developed and utilized effectively in several specialties, most prominently in rheumatology through the Outcome Measures in Rheumatology (OMERACT) initiative.[26] Protocols have been proposed for COS development in other areas of health research[27-33] and to facilitate this activity the Core Outcome Measures in Effectiveness Trials (COMET) initiative has begun.[34] Implementation of a successful COS should reduce heterogeneity in outcome reporting, enhance the quality of evidence synthesis and systematic reviews, and increase the relevance of clinical research for multiple stakeholders.[35]

This protocol establishes the context and scope for COS development in IBD, outlines the methods to be adopted for each step of COS development, and increases awareness of this effort to encourage IBD researchers and other stakeholders from around the world to participate.

For peer review only - http://bmjopen.bmj.com/site/about/guidelines.xhtml

METHODS AND ANALYSIS

Our interest in developing this COS has been listed in the non-database list of the COMET initiative (www.comet-initiative.org). This project will use published recommendations[24] for the development of an international consensus IBD-specific COS in a multi-step process. Detailed methodology for each step of the process is provided in the relevant sections below.

1) Completion of a systematic review to identify efficacy and safety outcomes currently reported in IBD randomized controlled trials
2) Identification of additional outcomes important to key stakeholders, including IBD patients and patient advocacy groups, clinicians, researchers, pharmaceutical industry representatives, health care payers, regulators and policy makers through semi-structured stakeholder interviews
3) Prioritization of outcomes and generation of a consensus outcomes list using a two-round Delphi survey[36]
4) Ratification of the COS in a consensus meeting of global experts

Scope of the core outcome set

This COS is intended as the international standard for clinical trials examining the efficacy of treatments in adult patients (≥18 years) with IBD. Patients included within the scope of this COS include those with:

1) Crohn's disease – including both luminal and peri-anal fistulizing disease
2) Ulcerative colitis – including patients with pouchitis after colectomy

For peer review only - http://bmjopen.bmj.com/site/about/guidelines.xhtml

Health interventions included within the scope of this COS include trials of therapeutic compounds and treatment algorithms. Effectiveness of surgical interventions will not be evaluated in this COS.

Identifying existing knowledge

To our knowledge, two existing initiatives have potential conceptual overlaps with the development of a COS. However, both projects have differing aims and neither of these identified projects have the same scope as the COS:

1) The International Consortium for Health Outcomes Measurement (ICHOM) is developing a standardized outcome set for IBD.[37] The ICHOM initiative is centered on devising patient- and value-based health care outcomes, which is most relevant as a quality metric for healthcare payers, with a broader scope on healthcare provision rather than a specific focus on core outcomes for assessment in clinical trials.
2) The Selecting Therapeutic Targets in Inflammatory Bowel Disease (STRIDE) program was initiated by the International Organization for the Study of Inflammatory Bowel Diseases (IOIBD).[6] Their recommendations for clinical, endoscopic, histologic, imaging, biomarker, and patient-reported targets in CD and UC aim to guide clinical practice rather than drive endpoint selection for clinical trials and drug development.

Step 1: Systematic literature review

For peer review only

For peer review only - http://bmjopen.bmj.com/site/about/guidelines.xhtml

A literature review will be conducted to identify and compare outcomes reported in existing studies of interventions for adult IBD patients. No sources of financial support will be used for the systematic review.

Types of studies, participants, and interventions

RCTs and systematic reviews of RCTs (with or without meta-analysis) will be included. Studies not describing IBD treatment outcomes, conference proceedings/abstracts without complete trial description, or studies for which full-text is not available in English will be excluded. Trial participants will include all adult IBD patients (≥18 years), including specific subgroups of patients with peri-anal fistulizing CD and UC patients developing pouchitis after restorative proctocolectomy. Interventions will include trials of therapeutic compounds (including systemic and topical corticosteroids, anti-inflammatories and mesalamine compounds, immune modulating agents, pre- and probiotic therapies, biologic and biosimilar therapies, fecal microbiota transplantation, and small molecule therapy) and trials of management algorithms applied to IBD patients. Both effectiveness and safety outcomes will be assessed. Surgical interventions will be excluded.

Search methods for identification of studies and study eligibility

Full terms of a comprehensive, electronic search strategy developed in accordance with the Preferred Reporting Items for Systematic Reviews and Meta-Analyses (PRISMA) guidelines are detailed in Supplemental Files 1 and 2.[38] The search strategy will be applied to MEDLINE, PubMed, EMBASE, and the Cochrane Central Register of

For peer review only

For peer review only - http://bmjopen.bmj.com/site/about/guidelines.xhtml

Controlled Trials (CENTRAL). ClinicalTrials.gov will be searched for relevant projects currently underway and we will also screen abstracts from the American College of Gastroenterology Annual Scientific Meeting, Digestive Disease Week, United European Gastroenterology Week, and European Crohn's and Colitis Organization conference proceedings published from January 2007 through June 2016. The reference lists of relevant studies will be searched for additional studies not identified from the electronic database search. No language restrictions will be applied to the initial search strategy but studies without English-language full text will be excluded from the selection of relevant articles. Given the substantial changes in IBD trial design over the past two decades, we will restrict the search to studies published after 1998 to ensure selection of more contemporary and relevant outcomes. Two review authors (CM and CEP) will independently screen the abstracts returned from the search strategy and any studies not meeting inclusion criteria will be excluded. In cases of dispute, a third review author (VJ) will be consulted.

Assessment of methodologic quality

As the primary focus of the systematic review will be to generate a list of potential outcome measures, the methodologic quality of the reported outcomes in included studies will be assessed using four questions[39]:

1) Is the primary outcome clearly stated?
2) Is the primary outcome clearly defined so that another researcher would be able to reproduce its measurement (e.g. measurement tools, measurement timing)?
3) Are secondary outcomes clearly stated?

For peer review only - http://bmjopen.bmj.com/site/about/guidelines.xhtml

4) Are secondary outcomes clearly defined?

As the primary scope of this project evaluates outcome reporting, the overall methodological quality of the included studies from systematic reviews will not be evaluated.

Data extraction, analysis, and presentation

Independent data extraction will be performed by two review authors (CM and CEP) using a standardized extraction form for the following: author details and affiliation, year and journal of publication, study design, study population (CD, UC, peri-anal fistulizing CD and pouchitis), intervention(s) under review, primary and secondary effectiveness and safety outcome(s) reported, outcome definition(s), and outcome measurement tool(s). Disagreement will be resolved through discussion and if resolution is not possible, a third reviewer (VJ) will be consulted. Original study authors will be contacted if there is unclear/unavailable data. The data will be synthesized and presented in a descriptive table, with all reported outcome measures and the quality of outcome reporting. Efficacy outcomes will be stratified by category: clinical, endoscopic, histologic, radiologic, laboratory, patient-reported, and composite scales of multiple outcome measures. Safety outcomes will be stratified by adverse event type (e.g. infections, cardiac adverse events, malignancies, lymphoma, infusion/injection reactions, immunologic adverse events) and by severity (hospitalization, intervention discontinuation, death). These outcomes will then be condensed into a preliminary list for consideration in semi-structured interviews and the Delphi survey.

For peer review only - http://bmjopen.bmj.com/site/about/guidelines.xhtml

Records will be managed in EndNote™ reference software (Clarivate Analytics, Boston, MA).

Step 2: Stakeholder involvement

Outcomes measured in clinical trials must be meaningful to patients, health care providers, and health care systems who receive, deliver, and pay for care, respectively. Therefore, the input of multiple stakeholders affected by a COS for IBD trials will be sought. Semi-structured interviews will be conducted with the following aims:

1) Preliminary prioritization of the importance of efficacy and safety outcome measures generated through the systematic review
2) Augmentation of this list with additional items considered important to stakeholders but not captured in the literature

Stakeholder interview participants and recruitment

We will engage and conduct interviews with the following stakeholder groups: 1) patients with IBD; 2) specialists caring for patients with IBD, including gastroenterologists, surgeons, and specialist nurses; 3) representatives from patient advocacy groups; 4) representatives from the pharmaceutical industry and; (5) representatives from regulatory agencies (e.g. FDA, European Medicines Agency, Health Canada). Participants will be purposively sampled to obtain a comprehensive representation in demographics, patient clinical characteristics, treatment experiences, and professional expertise. Sample size will be estimated pragmatically to achieve

For peer review only - http://bmjopen.bmj.com/site/about/guidelines.xhtml

saturation of views represented in the qualitative data. An initial sample size of 30 interviews is estimated, or at theme saturation.

Data collection and analysis

Qualitative semi-structured interviews will be conducted, allowing all participants to raise issues considered of greatest importance. A topic guide will be provided to ensure all interviews address critical topics pertaining to COS development, including: 1) patient experiences of living with IBD and the benefits and harms of IBD-related treatment; 2) outcomes believed to be relevant and important to include in IBD trials and why; 3) measurement tools for use in IBD clinical trials that are effective, reliable, and practical; and 4) relative importance of outcomes identified from the systematic review. Face-to-face or telephone interviews lasting 30-60 minutes will be conducted by experts in qualitative methods and all interviews will be recorded and transcribed verbatim. Recordings will be imported into qualitative analysis software and narrative data will then be indexed and mapped to a thematic framework, providing a summary of participants' key points and priorities.[40]

Step 3: Delphi survey

An international Delphi survey, informed by literature review and semi-structured stakeholder interviews, will then be performed to achieve consensus on the outcomes for inclusion in the COS. The Delphi method allows panel members to anonymously derive consensus through multiple rounds of sequential questionnaires. After each round, the group responses are provided to panelists who can then reconsider their

For peer review only - http://bmjopen.bmj.com/site/about/guidelines.xhtml

position in light of other viewpoints. The anonymity of the Delphi method avoids the opinions of prominent personalities from dominating the consensus and also facilitates wide international participation.[36] The Delphi process will consist of two rounds of electronic-based questionnaire, response, and feedback. All electronic questionnaires will be pilot tested prior to distribution to ensure clarity.

Selection of panel members

For this study, the Delphi panel will include a minimum target sample size of 50 respondents. We aim to recruit a diverse participant pool, with involvement from each major stakeholder group, including patients, clinicians, researchers, and representatives from patient advocacy groups, industry, and research funding organizations. Selected participants will reflect a broad range of clinical experiences and geographical expertise, with representation from Canada, the United States, the United Kingdom, continental Europe, and the Asia-Pacific.

Researchers with extensive experience in IBD will be sought for the Delphi survey. During the systematic review, a list of authors with at least 25 publications in the field of IBD over the past 10 years (2006-2016), including at least two clinical trials or one systematic review of clinical trials on IBD will be compiled and invited to participate. The lead and corresponding authors of clinical trials or systematic reviews will be preferentially invited to participate. Clinicians experienced in managing IBD will be recruited through convenience sampling. Specifically, clinical medical and surgical leads of dedicated IBD centers from North America, Europe, and the Asia-Pacific will be

For peer review only - http://bmjopen.bmj.com/site/about/guidelines.xhtml

identified and recruited; this recruitment strategy has been previously used by other COS developers.[28 29]

Patients will be eligible for inclusion in the Delphi survey if they have a confirmed history of CD or UC, attendance of healthcare for IBD, and fluent understanding of written English. Patients will be identified through national and international patient advocacy groups and authors' connections. Strong collaborative partnerships between the authorship team and IBD centers in Europe and the Asia-Pacific will aim to incorporate multi-national patient representation. Representatives from the pharmaceutical industry will also be invited to participate; this group will comprise approximately 10% of Delphi survey participants.

All potential participants will be emailed an invitation letter outlining the aims and details of the study and the rationale and importance of completing the entire Delphi process. Respondents who agree to take part will be assigned a unique identification number. For each round of the process, participants will have three weeks to complete the survey with generic email reminders sent at the one and two week marks. All data will be stored against the unique identifier only; participants will be blinded to the other respondents in the study. Only the lead author (CM) and primary investigator (VJ) will have access to the complete list of Delphi survey panelists. For each round of the Delphi survey, response and attrition rates will be calculated.

Delphi round one

For peer review only - http://bmjopen.bmj.com/site/about/guidelines.xhtml

In the first round, participants will be asked to identify the stakeholder group to which they belong, and complete questions about their professional background and experience with clinical research relevant to IBD. They will then be presented with the complete list of efficacy and safety outcomes generated from the literature review and stakeholder interviews. Outcome order will be randomly assigned to mitigate the influence of display order on scoring. Participants will be asked to rank each outcome on a scale from 1 to 9, based on the Grading of Recommendations Assessment, Development, and Evaluation (GRADE) working group definitions.[41] Scores of 1-3 indicate an outcome that is not important for inclusion, scores of 4-6 indicate an outcome important but not critical for inclusion, and scores of 7-9 indicate an outcome felt critical for inclusion in the COS. An option to select “Unsure of significance” will also be available. Participants will be asked to focus on ranking the most important outcomes for inclusion highly and excluding outcomes felt to be of lesser importance; regardless of score, all outcomes will be carried to the second round. Finally, through free text entry, participants will have the option to clarify compelling arguments for and against inclusion of outcomes and to identify additional outcomes not included in the first round questionnaire.

Responses from round one will be analyzed and collated into a feedback report. Descriptive statistics will be used to summarize the number of participants scoring each outcome and the distribution of scores. Responses to open-ended questions will be reviewed by the authorship team to evaluate for substantial arguments and additional suggestions will be reviewed for uncaptured outcomes in the first round questionnaire.

For peer review only - http://bmjopen.bmj.com/site/about/guidelines.xhtml

Subgroup analysis will be conducted, stratifying scores by stakeholder group to evaluate for differences from other panelist responses. Panelists who do not complete the first round survey will not be invited to participate in round two.

Delphi round two

In round two, each participant will be provided with the number of respondents and distribution of scores for each efficacy and safety outcome from the first round, stratified by stakeholder group. They will then be shown their own score from round one and asked to rescore each outcome, with consideration based on insights from the group. Each outcome will be rescored on a scale from 1-9 as previously described and participants will be specifically asked whether each outcome should be included in the COS. Changes in score from round-to-round will be documented.

Responses from round two will be analyzed with descriptive statistics. Outcomes for which ≥70% of panelists scored it 7 to 9 and fewer than 15% of panelists scored it 1 to 3 will be decided *a priori* to have met consensus for inclusion.[24] Conversely, outcomes for which ≥70% of panelists scored it 1 to 3, and fewer than 15% of panelists scored it 7 to 9 will be defined to have met consensus for exclusion. Outcomes not meeting these definitions will be classified as lack of consensus. While these definitions are subjective, they have been recommended by previous COS authors [24] and avoid *post-hoc* definitions of consensus that may bias the results.

Step 4: Consensus meeting

For peer review only - http://bmjopen.bmj.com/site/about/guidelines.xhtml

A face-to-face consensus meeting with key stakeholders will be held after completion of the Delphi process. The meeting will be chaired by an independent facilitator with the objective of finalizing the outcomes for inclusion in the COS. Participants will be purposively sampled from panelists completing both rounds of the Delphi study; approximately 30 participants from diverse stakeholder groups will be invited to participate. The results from each round of the Delphi survey will be reviewed and participants will ratify the efficacy and safety outcomes that meet consensus criteria for inclusion and exclusion. Participants will then discuss the outcomes for which there was lack of agreement; based on the discussion, participants will then anonymously vote for each outcome for inclusion and exclusion in the finalized COS using a format similar to that of the Delphi survey.

For peer review only

For peer review only - http://bmjopen.bmj.com/site/about/guidelines.xhtml

ETHICS AND DISSEMINATION

Ethical Considerations

As with previous COS development projects, this project is considered a service evaluation not directly influencing patient care or safety.[27 42] All participants involved will be asked for their consent before participating in either stakeholder interviews or the Delphi survey, and all procedures will be conducted according to the Declaration of Helsinki.

Dissemination

With over 30 novel therapeutic compounds in various stages of clinical development[43], the adoption of an international consensus COS will be critical in ensuring future clinical trials report valid, meaningful, and standardized efficacy outcomes. This need is particularly exigent, commensurate with the transition from traditional symptom-based outcomes such as the Crohn's Disease Activity Index and Mayo Clinic score, to a diverse array of endoscopic, histologic, radiographic, and patient-reported endpoints. Additionally, with the increasing adoption of biologic therapies for IBD management, it is essential for clinical trials to identify unique safety considerations associated with novel therapies. Reporting of treatment-specific safety outcomes such as infectious, malignant, immune, surgical, and drug-related adverse events may promote the development of future preventative strategies for optimizing short- and long-term patient safety. Through this COS, we intend to reduce outcome reporting bias, reduce reporting heterogeneity, improve clinical trial quality in IBD, and facilitate more robust data synthesis of treatment interventions.

For peer review only - http://bmjopen.bmj.com/site/about/guidelines.xhtml

A finalized COS reporting guideline and explanatory document will be drafted, including all efficacy and safety outcomes and measurements as determined by the Delphi rounds and consensus meeting. These documents will be disseminated by high impact publication.

For peer review only

For peer review only - http://bmjopen.bmj.com/site/about/guidelines.xhtml

DECLARATIONS

Authorship Contributions

CM and VJ were involved in study conception and manuscript drafting and editing. RP, RNF, BGF, WJS and CEP were involved in study conception and manuscript editing. RK and BGL were involved in manuscript editing for important intellectual content. VJ is the guarantor of the article.

Data Sharing Statement

All data from the project will be available upon request from the corresponding author.

Competing interests

Christopher Ma has no conflicts of interest to declare

Remo Panaccione has received scientific advisory board fees from Abbott/AbbVie, Amgen, Janssen, Merck, Pfizer, Prometheus Laboratories, Salix Pharma, Shire, Takeda, Warner Chilcott; consulting fees from Abbott/AbbVie, Amgen, Aptalis, Astra Zeneca, Baxter, BMS, Centocor, Elan/Biogen, Eisai, Ferring, GSK, Janssen, Merck, Millennium, Pfizer, Proctor & Gamble, Prometheus Therapeutics and Diagnostics, Schering-Plough, Shire, Takeda, UCB Pharma, Warner Chilcott; research grants from Abbott/AbbVie, Amgen, Aptalis, Astra Zeneca, Baxter, BMS, Centocor, Eisai, Elan/Biogen, Ferring, GSK, Janssen, Merck, Millennium, Pfizer, Proctor & Gamble, Prometheus, Shire, Schering-Plough, Takeda, UCB Pharma, Warner Chilcott; and speaker's bureau fees from Abbott/AbbVie, Amgen, Aptalis, Astra Zeneca, Baxter, BMS, Centocor, Eisai, Elan/Biogen, Ferring, GSK, Janssen, Merck, Millennium, Pfizer, Proctor & Gamble, Prometheus, Schering-Plough, Shire, Takeda, UCB Pharma, Warner Chilcott

Richard Fedorak has received scientific advisory board fees from Abbott/AbbVie, Celltrion, Ferring, Janssen, Shire, VSL#3; consulting fees from Abbott/AbbVie, Celltrion, Ferring, Janssen, Shire, VSL#3; and research grant support from Abbott/AbbVie, Alba Therapeutics, BMS, Celltrion, Centocor, Genentech, GSK, Janssen, Merck, Millennium, Novartis, Pfizer, Proctor & Gamble, Roche, VSL#3

Claire Parker has no conflicts of interest to declare

For peer review only - http://bmjopen.bmj.com/site/about/guidelines.xhtml

Reena Khanna has received consulting fees from AbbVie, Takeda, and Janssen

Barrett Levesque has received consulting fees from AbbVie, Takeda, Nestle Health Sciences, and Prometheus Labs

William Sandborn has served as a consultant to: AbbVie Inc., ActoGeniX NV, AGI Therapeutics, Inc., Alba Therapeutics Corporation, Albireo, Alfa Wasserman, Amgen, AM-Pharma BV, Anaphore, Astellas Pharma, Athersys, Inc., Atlantic Healthcare Limited, Axcan Pharma (now Aptalis), BioBalance Corporation, Boehringer-Ingelheim Inc, Bristol Meyers Squibb, Celgene, Celek Pharmaceuticals, Cellerix SL, Cerimon Pharmaceuticals, ChemoCentryx, CoMentis, Cosmo Technologies, Coronado Biosciences, Cytokine Pharmasciences, Eagle Pharmaceuticals, Eisai Medical Research Inc., Elan Pharmaceuticals, EnGene, Inc., Eli Lilly, Enteromedics, Exagen Diagnostics, Inc., Ferring Pharmaceuticals, Flexion Therapeutics, Inc., Funxional Therapeutics Limited, Genzyme Corporation, Genentech (now Roche), Gilead Sciences, Given Imaging, Glaxo Smith Kline, Human Genome Sciences, Ironwood Pharmaceuticals (previously Microbia Inc.), Janssen (previously Centocor), KaloBios Pharmaceuticals, Inc., Lexicon Pharmaceuticals, Lycera Corporation, Meda Pharmaceuticals (previously Alaven Pharmaceuticals), Merck Research Laboratories, MerckSerono, Millennium Pharmaceuticals (subsequently merged with Takeda), Nisshin Kyorin Pharmaceuticals Co., Ltd., Novo Nordisk A/S, NPS Pharmaceuticals, Optimer Pharmaceuticals, Orexigen Therapeutics, Inc., PDL Biopharma, Pfizer, Procter and Gamble, Prometheus Laboratories, ProtAb Limited, Purgenesis Technologies, Inc., Receptos, Relypsa, Inc., Salient Pharmaceuticals, Salix Pharmaceuticals, Inc., Santarus, Schering Plough Corporation (acquired by Merck), Shire Pharmaceuticals, Sigmoid Pharma Limited, Sirtris Pharmaceuticals, Inc. (a GSK company), S.L.A. Pharma (UK) Limited, Targacept, Teva Pharmaceuticals, Therakos, Tillotts Pharma AG (acquired by Zeria Pharmaceutical Co., Ltd), TxCell SA, UCB Pharma, Viamet Pharmaceuticals, Vascular Biogenics Limited (VBL), Warner Chilcott UK Limited; has received speaker's fees from: AbbVie Inc., Bristol Meyers Squibb, and Janssen (previously Centocor); and financial support for research from: AbbVie Inc., Bristol Meyers Squibb, Genentech, Glaxo Smith Kline, Janssen (previously Centocor), Millennium Pharmaceuticals (now Takeda), Novartis, Pfizer, Procter and Gamble Pharmaceuticals, Shire Pharmaceuticals, and UCB Pharma.

Brian Feagan has received grant/research support from Millennium Pharmaceuticals, Merck, Tillotts Pharma AG, AbbVie, Novartis Pharmaceuticals, Centocor Inc., Elan/Biogen, UCB Pharma, Bristol-Myers Squibb, Genentech, ActoGenix, and Wyeth Pharmaceuticals Inc.; consulting fees from Millennium Pharmaceuticals, Merck, Centocor Inc., Elan/Biogen, Janssen-Ortho, Teva Pharmaceuticals, Bristol-Myers Squibb, Celgene, UCB Pharma, AbbVie, Astra Zeneca, Serono, Genentech, Tillotts Pharma AG, Unity Pharmaceuticals, Albireo Pharma, Given Imaging Inc., Salix Pharmaceuticals, Novonordisk, GSK, Actogenix, Prometheus Therapeutics and Diagnostics, Athersys, Axcan, Gilead, Pfizer, Shire, Wyeth, Zealand Pharma, Zyngenia, GiCare Pharma Inc., and Sigmoid Pharma; and speakers bureaux fees from UCB, AbbVie, and J&J/Janssen

For peer review only - http://bmjopen.bmj.com/site/about/guidelines.xhtml

554
555 Vipul Jairath has received scientific advisory board fees from AbbVie, Sandoz, Takeda,
556 Janssen; speakers fees from Takeda, Janssen, Shire, Ferring

For peer review only

For peer review only - http://bmjopen.bmj.com/site/about/guidelines.xhtml

Abbreviations

CD (Crohn's disease); CDAI (Crohn's Disease Activity Index); CENTRAL (Cochrane Central Register of Controlled Trials); COMET (Core Outcome Measures in Effectiveness Trials); COS (core outcome set); GRADE (Grading of Recommendations Assessment, Development, and Evaluation); IBD (inflammatory bowel disease); ICHOM (International Consortium for Health Outcomes Measurement); OMERACT (Outcome Measures in Rheumatology); PRISMA (Preferred Reporting Items for Systematic Reviews and Meta-Analyses); PRO (patient reported outcome); RCT (randomized controlled trial); UC (ulcerative colitis); STRIDE (Selecting Therapeutic Targets in Inflammatory Bowel Disease)

For peer review only

For peer review only - http://bmjopen.bmj.com/site/about/guidelines.xhtml

REFERENCES

1. Molodecky NA, Soon IS, Rabi DM, et al. Increasing incidence and prevalence of the inflammatory bowel diseases with time, based on systematic review. *Gastroenterology* 2012;142(1):46-54. doi: 10.1053/j.gastro.2011.10.001

2. Peyrin-Biroulet L, Loftus EV, Jr., Colombel JF, et al. The natural history of adult Crohn's disease in population-based cohorts. *Am J Gastroenterol* 2010;105(2):289-97. doi: 10.1038/ajg.2009.579

3. de Souza HS, Fiocchi C. Immunopathogenesis of IBD: current state of the art. *Nat Rev Gastroenterol Hepatol* 2016;13(1):13-27. doi: 10.1038/nrgastro.2015.186

4. Gibson TB, Ng E, Ozminkowski RJ, et al. The direct and indirect cost burden of Crohn's disease and ulcerative colitis. *J Occup Environ Med* 2008;50(11):1261-72. doi: 10.1097/JOM.0b013e318181b8ca

5. Kappelman MD, Rifas-Shiman SL, Porter CQ, et al. Direct health care costs of Crohn's disease and ulcerative colitis in US children and adults. *Gastroenterology* 2008;135(6):1907-13. doi: 10.1053/j.gastro.2008.09.012

6. Peyrin-Biroulet L, Sandborn W, Sands BE, et al. Selecting Therapeutic Targets in Inflammatory Bowel Disease (STRIDE): Determining Therapeutic Goals for Treat-to-Target. *Am J Gastroenterol* 2015;110(9):1324-38. doi: 10.1038/ajg.2015.233

7. Reinisch W, Sandborn WJ, Hommes DW, et al. Adalimumab for induction of clinical remission in moderately to severely active ulcerative colitis: results of a randomised controlled trial. *Gut* 2011;60(6):780-7. doi: 10.1136/gut.2010.221127

For peer review only - http://bmjopen.bmj.com/site/about/guidelines.xhtml

8. Sandborn WJ, van Assche G, Reinisch W, et al. Adalimumab induces and maintains clinical remission in patients with moderate-to-severe ulcerative colitis. *Gastroenterology* 2012;142(2):257-65 e1-3. doi: 10.1053/j.gastro.2011.10.032

9. Rutgeerts P, Sandborn WJ, Feagan BG, et al. Infliximab for induction and maintenance therapy for ulcerative colitis. *N Engl J Med* 2005;353(23):2462-76. doi: 10.1056/NEJMoa050516

10. Feagan BG, Rutgeerts P, Sands BE, et al. Vedolizumab as induction and maintenance therapy for ulcerative colitis. *N Engl J Med* 2013;369(8):699-710. doi: 10.1056/NEJMoa1215734

11. Hanauer SB, Feagan BG, Lichtenstein GR, et al. Maintenance infliximab for Crohn's disease: the ACCENT I randomised trial. *Lancet* 2002;359(9317):1541-9. doi: 10.1016/S0140-6736(02)08512-4

12. Hanauer SB, Sandborn WJ, Rutgeerts P, et al. Human anti-tumor necrosis factor monoclonal antibody (adalimumab) in Crohn's disease: the CLASSIC-I trial. *Gastroenterology* 2006;130(2):323-33; quiz 591. doi: 10.1053/j.gastro.2005.11.030

13. Colombel JF, Sandborn WJ, Rutgeerts P, et al. Adalimumab for maintenance of clinical response and remission in patients with Crohn's disease: the CHARM trial. *Gastroenterology* 2007;132(1):52-65. doi: 10.1053/j.gastro.2006.11.041

14. Sandborn WJ, Feagan BG, Rutgeerts P, et al. Vedolizumab as induction and maintenance therapy for Crohn's disease. *N Engl J Med* 2013;369(8):711-21. doi: 10.1056/NEJMoa1215739

For peer review only

For peer review only - http://bmjopen.bmj.com/site/about/guidelines.xhtml

15. Khanna R, Jairath V, Vande Casteele N, et al. Efficient Early Drug Development for Ulcerative Colitis. *Gastroenterology* 2016;150(5):1056-60. doi: 10.1053/j.gastro.2016.03.013

16. Jairath V, Levesque BG, Vande Casteele N, et al. Evolving Concepts in Phases I and II Drug Development for Crohn's Disease. *J Crohns Colitis* 2016 doi: 10.1093/ecco-jcc/jjw137

17. Hindryckx P, Baert F, Hart A, et al. Clinical trials in luminal Crohn's disease: a historical perspective. *J Crohns Colitis* 2014;8(11):1339-50. doi: 10.1016/j.crohns.2014.04.007

18. Best WR, Becktel JM, Singleton JW, et al. Development of a Crohn's disease activity index. National Cooperative Crohn's Disease Study. *Gastroenterology* 1976;70(3):439-44.

19. Schroeder KW, Tremaine WJ, Ilstrup DM. Coated oral 5-aminosalicylic acid therapy for mildly to moderately active ulcerative colitis. A randomized study. *N Engl J Med* 1987;317(26):1625-9. doi: 10.1056/NEJM198712243172603

20. Williet N, Sandborn WJ, Peyrin-Biroulet L. Patient-reported outcomes as primary end points in clinical trials of inflammatory bowel disease. *Clin Gastroenterol Hepatol* 2014;12(8):1246-56 e6. doi: 10.1016/j.cgh.2014.02.016

21. Targownik LE, Sexton KA, Bernstein MT, et al. The Relationship Among Perceived Stress, Symptoms, and Inflammation in Persons With Inflammatory Bowel Disease. *Am J Gastroenterol* 2015;110(7):1001-12; quiz 13. doi: 10.1038/ajg.2015.147

For peer review only

For peer review only - http://bmjopen.bmj.com/site/about/guidelines.xhtml

22. Gracie DJ, Williams CJ, Sood R, et al. Poor Correlation Between Clinical Disease Activity and Mucosal Inflammation, and the Role of Psychological Comorbidity, in Inflammatory Bowel Disease. *Am J Gastroenterol* 2016;111(4):541-51. doi: 10.1038/ajg.2016.59

23. Bonovas S, Fiorino G, Allocca M, et al. Biologic Therapies and Risk of Infection and Malignancy in Patients With Inflammatory Bowel Disease: A Systematic Review and Network Meta-analysis. *Clin Gastroenterol Hepatol* 2016;14(10):1385-97 e10. doi: 10.1016/j.cgh.2016.04.039

24. Williamson PR, Altman DG, Blazeby JM, et al. Developing core outcome sets for clinical trials: issues to consider. *Trials* 2012;13:132. doi: 10.1186/1745-6215-13-132

25. D'Haens G, Feagan B, Colombel JF, et al. Challenges to the design, execution, and analysis of randomized controlled trials for inflammatory bowel disease. *Gastroenterology* 2012;143(6):1461-9. doi: 10.1053/j.gastro.2012.09.031

26. Boers M, Kirwan JR, Wells G, et al. Developing core outcome measurement sets for clinical trials: OMERACT filter 2.0. *J Clin Epidemiol* 2014;67(7):745-53. doi: 10.1016/j.jclinepi.2013.11.013

27. Chiarotto A, Terwee CB, Deyo RA, et al. A core outcome set for clinical trials on non-specific low back pain: study protocol for the development of a core domain set. *Trials* 2014;15:511. doi: 10.1186/1745-6215-15-511

28. Egan AM, Smith V, Devane D, et al. Effectiveness of prepregnancy care for women with pregestational diabetes mellitus: protocol for a systematic review of the

For peer review only - http://bmjopen.bmj.com/site/about/guidelines.xhtml

literature and identification of a core outcomes set using a Delphi survey. *Trials* 2015;16:356. doi: 10.1186/s13063-015-0894-8

29. Harman NL, Bruce IA, Callery P, et al. MOMENT--Management of Otitis Media with Effusion in Cleft Palate: protocol for a systematic review of the literature and identification of a core outcome set using a Delphi survey. *Trials* 2013;14:70. doi: 10.1186/1745-6215-14-70

30. Iyengar S, Williamson PR, Schmitt J, et al. Development of a core outcome set for clinical trials in rosacea: study protocol for a systematic review of the literature and identification of a core outcome set using a Delphi survey. *Trials* 2016;17(1):429. doi: 10.1186/s13063-016-1554-3

31. Kelly LE, Jansson LM, Moulsdale W, et al. A core outcome set for neonatal abstinence syndrome: study protocol for a systematic review, parent interviews and a Delphi survey. *Trials* 2016;17(1):536. doi: 10.1186/s13063-016-1666-9

32. MacLennan S, Bekema HJ, Williamson PR, et al. A core outcome set for localised prostate cancer effectiveness trials: protocol for a systematic review of the literature and stakeholder involvement through interviews and a Delphi survey. *Trials* 2015;16:76. doi: 10.1186/s13063-015-0598-0

33. Tong A, Manns B, Hemmelgarn B, et al. Standardised outcomes in nephrology - Haemodialysis (SONG-HD): study protocol for establishing a core outcome set in haemodialysis. *Trials* 2015;16:364. doi: 10.1186/s13063-015-0895-7

34. Gargon E. The COMET (Core Outcome Measures in Effectiveness Trials) Initiative. *Maturitas* 2016;91:91-2. doi: 10.1016/j.maturitas.2016.06.007

For peer review only - http://bmjopen.bmj.com/site/about/guidelines.xhtml

35. Kirkham JJ, Gorst S, Altman DG, et al. COS-STAR: a reporting guideline for studies developing core outcome sets (protocol). *Trials* 2015;16:373. doi: 10.1186/s13063-015-0913-9

36. Sinha IP, Smyth RL, Williamson PR. Using the Delphi technique to determine which outcomes to measure in clinical trials: recommendations for the future based on a systematic review of existing studies. *PLoS Med* 2011;8(1):e1000393. doi: 10.1371/journal.pmed.1000393

37. The ICHOM Standard Set for Inflammatory Bowel Disease [Available from: http://www.ichom.org/medical-conditions/inflammatory-bowel-disease/2016.

38. Moher D, Liberati A, Tetzlaff J, et al. Preferred reporting items for systematic reviews and meta-analyses: the PRISMA statement. *J Clin Epidemiol* 2009;62(10):1006-12. doi: 10.1016/j.jclinepi.2009.06.005

39. Mokkink LB, Terwee CB, Knol DL, et al. The COSMIN checklist for evaluating the methodological quality of studies on measurement properties: a clarification of its content. *BMC Med Res Methodol* 2010;10:22. doi: 10.1186/1471-2288-10-22

40. Kuper A, Reeves S, Levinson W. An introduction to reading and appraising qualitative research. *BMJ* 2008;337:a288. doi: 10.1136/bmj.a288

41. Guyatt GH, Oxman AD, Kunz R, et al. GRADE guidelines: 2. Framing the question and deciding on important outcomes. *J Clin Epidemiol* 2011;64(4):395-400. doi: 10.1016/j.jclinepi.2010.09.012

42. Hirsch M, Duffy JM, Barker C, et al. Protocol for developing, disseminating and implementing a core outcome set for endometriosis. *BMJ Open* 2016;6(12):e013998. doi: 10.1136/bmjopen-2016-013998

For peer review only - http://bmjopen.bmj.com/site/about/guidelines.xhtml

43. Amiot A, Peyrin-Biroulet L. Current, new and future biological agents on the horizon for the treatment of inflammatory bowel diseases. *Therap Adv Gastroenterol* 2015;8(2):66-82. doi: 10.1177/1756283X14558193

For peer review only

For peer review only - http://bmjopen.bmj.com/site/about/guidelines.xhtml

Appendix

Table a: Crude and adjusted odd ratio (OR) and 95% confidence interval from multivariable logistic regression of autistic spectrum disorder (ASD) for first month of second trimester, by socioeconomic status (SES)

**Month of second trimester**	**OR crude**	**95%CI**		**OR adjusted***	**95%CI**		**OR crude**	**95%CI**		**OR adjusted***	**95%CI**	
**Jan**	1 (ref.)			1 (ref.			1 (ref.)			1 (ref.		
**Feb**	.83	.51	1.35	.83	.51	1.36	1.03	.67	1.57	1.03	.67	1.57
**Mar**	1.08	.69	1.69	1.08	.69	1.69	.87	.57	1.35	.86	.56	1.33
**Apr**	.89	.55	1.43	.89	.55	1.43	1.12	.74	1.69	1.11	.73	1.67
**May**	.68	.41	1.12	.68	.41	1.13	.99	.65	1.51	.98	.64	1.49
**Jun**	.87	.53	1.41	.87	.54	1.41	1.09	.72	1.64	1.07	.71	1.63
**Jul**	1.00	.62	1.59	.98	.61	1.56	1.02	.67	1.56	1.04	.68	1.59
**Aug**	.74	.44	1.23	.72	.43	1.20	.97	.63	1.49	.97	.63	1.49
**Sep**	.94	.58	1.54	.93	.57	1.51	.86	.55	1.35	.87	.55	1.36
**Oct**	.84	.51	1.37	.83	.51	1.37	1.10	.73	1.66	1.11	.73	1.67
**Nov**	.84	.51	1.40	.83	.50	1.38	1.25	.84	1.87	1.24	.83	1.86
**Dec**	.83	.51	1.36	.83	.51	1.36	1.10	.73	1.65	1.09	.72	1.64

*Adjusted for child's age, father's age, mother's age, district, birth weight, age with imputation (see text for details).

For peer review only

Table b: Crude and adjusted odd ratio (OR) and 95% confidence interval from multivariable logistic regression of autistic spectrum disorder (ASD) for first month of third trimester, by socioeconomic status (SES)

Month of third trimester	OR crude	95%CI		OR adjusted*	95%CI		OR crude	95%CI		OR adjusted*	95%CI	
Jan	1 (ref.)			1 (ref.			1 (ref.)			1 (ref.		
Feb	1.04	0.61	1.77	1.04	0.61	1.78	1.22	0.81	1.82	1.21	0.81	1.81
Mar	0.97	0.57	1.63	0.97	0.58	1.64	0.96	0.63	1.46	0.95	0.63	1.45
Apr	1.19	0.73	1.95	1.20	0.74	1.97	1.13	0.75	1.68	1.12	0.75	1.68
May	0.86	0.51	1.46	0.87	0.52	1.48	0.86	0.56	1.32	0.86	0.56	1.31
Jun	1.26	0.77	2.04	1.27	0.78	2.06	0.84	0.55	1.30	0.83	0.54	1.27
Jul	0.99	0.60	1.65	1.01	0.61	1.67	1.07	0.71	1.61	1.05	0.70	1.58
Aug	0.78	0.45	1.33	0.79	0.46	1.36	0.99	0.66	1.51	0.98	0.64	1.48
Sep	1.12	0.68	1.86	1.12	0.68	1.86	1.12	0.75	1.69	1.12	0.74	1.69
Oct	0.84	0.49	1.44	0.83	0.48	1.42	0.83	0.53	1.29	0.83	0.54	1.30
Nov	0.92	0.53	1.57	0.91	0.53	1.56	1.00	0.65	1.53	1.00	0.65	1.54
Dec	1.07	0.64	1.80	1.08	0.64	1.81	0.81	0.52	1.26	0.81	0.52	1.26

*Adjusted for child's age, father’s age, mother’s age, district, birth weight, age with imputation (see text for details).

Table c: Crude and adjusted odd ratio (OR) and 95% confidence interval from multivariable logistic regression of autistic spectrum disorder (ASD) for season of birth, by socioeconomic status (SES)

For peer review only - http://bmjopen.bmj.com/site/about/guidelines.xhtml

	SES median-high						SES median-low					
**Season of birth**	**OR crude**	**95%CI**		**OR adjusted***	**95%CI**		**OR crude**	**95%CI**		**OR adjusted***	**95%CI**	
Winter	1 (ref.)			1 (ref.			1 (ref.)			1 (ref.		
Spring	1.12	0.84	1.50	1.12	0.84	1.50	1.06	0.83	1.34	1.06	0.83	1.34
Summer	1.24	0.94	1.63	1.23	0.93	1.63	1.08	0.85	1.37	1.08	0.85	1.37
Fall	1.14	0.86	1.51	1.14	0.86	1.51	1.05	0.82	1.33	1.04	0.82	1.32

*Adjusted for child's age, father's age, mother's age, district, birth weight, gestational age with imputation

Table d: Crude and adjusted odd ratio (OR) and 95% confidence interval from multivariable logistic regression of autistic spectrum disorder (ASD) for season of conception, by socioeconomic status (SES)

For peer review only

	SES median-high						SES median-low					
**Season of conception**	**OR crude**	**95%CI**		**OR adjusted***	**95%CI**		**OR crude**	**95%CI**		**OR adjusted***	**95%CI**	
**Winter**	1 (ref.)			1 (ref.			1 (ref.)			1 (ref.		
**Spring**	1.00	0.75	1.34	0.99	0.74	1.33	0.96	0.75	1.22	0.96	0.75	1.23
**Summer**	1.00	0.74	1.35	0.99	0.73	1.33	0.96	0.75	1.22	0.97	0.76	1.24
**Fall**	1.09	0.82	1.45	1.08	0.82	1.44	1.05	0.83	1.33	1.06	0.83	1.34

*Adjusted for child's age, father's age, mother's age, district, birth weight, gestational age with imputation

For peer review only

Table e: Crude and adjusted odd ratio (OR) and 95% confidence interval from multivariable logistic regression of autistic spectrum disorder (ASD) for month of conception, by socioeconomic status (SES)

SES 1-6 SES 7-10

Month of conception	OR crude	95%CI		OR adjusted*	95%CI		OR crude	95%CI		OR adjusted*	95%CI	
Jan	1 (ref.)			1 (ref.			1 (ref.)			1 (ref.		
Feb	.89	.52	1.50	.89	.52	1.51	1.09	.72	1.65	1.08	.72	1.63
Mar	1.13	.70	1.85	1.14	.70	1.86	.84	.55	1.30	.84	.54	1.29
Apr	.90	.53	1.53	.89	.53	1.52	1.06	.71	1.60	1.09	.72	1.64
May	1.05	.63	1.75	1.03	.62	1.71	.94	.62	1.44	.95	.62	1.45
Jun	1.19	.72	1.96	1.17	.71	1.93	.74	.46	1.16	.75	.47	1.18
Jul	.79	.45	1.38	.79	.45	1.38	.96	.63	1.45	.97	.64	1.47
Aug	1.11	.67	1.83	1.09	.66	1.81	1.13	.76	1.68	1.13	.76	1.68
Sep	1.29	.79	2.10	1.28	.78	2.08	1.09	.73	1.64	1.10	.73	1.65
Oct	.95	.57	1.58	.96	.58	1.59	.88	.58	1.34	.89	.58	1.35
Nov	1.14	.70	1.86	1.14	.70	1.86	1.16	.78	1.72	1.16	.78	1.72
Dec	1.18	.74	1.91	1.18	.74	1.91	.90	.59	1.36	.89	.59	1.36

*Adjusted for child's age, father's age, mother's age, district, birth weight, age with imputation (see text for details).

For peer review only

For peer review only

For peer review only - http://bmjopen.bmj.com/site/about/guidelines.xhtml
